# Incretin-Based Drugs for Obesity: Common and Drug-Specific Reporting Patterns of Adverse Drug Reactions—A Comparative Disproportionality Analysis Using EudraVigilance Reports Integrating SmPC Data

**DOI:** 10.3390/ph19060876

**Published:** 2026-05-31

**Authors:** Ioana Rada Popa Ilie, Steliana Ghibu, Anca Butuca, Carmen Maximiliana Dobrea, Adina Frum, Calin Homorodean, Adriana Aurelia Chis, Claudiu Morgovan

**Affiliations:** 1 Endocrinology Department, Faculty of Medicine, “Iuliu Haţieganu” University of Medicine and Pharmacy, 3-5 Louis Pasteur Street, 400349 Cluj-Napoca, Romania; ioana.ilie@umfcluj.ro; 2 Department of Pharmacology, Physiology and Pathophysiology, Faculty of Pharmacy, “Iuliu Haţieganu” University of Medicine and Pharmacy, 6A Louis Pasteur Street, 400349 Cluj-Napoca, Romania; 3 Preclinical Department, Faculty of Medicine, “Lucian Blaga” University of Sibiu, 550169 Sibiu, Romania; anca.butuca@ulbsibiu.ro (A.B.); carmen.dobrea@ulbsibiu.ro (C.M.D.); adina.frum@ulbsibiu.ro (A.F.); claudiu.morgovan@ulbsibiu.ro (C.M.); 4 Internal Medicine Department, Medical Clinic No. 1, “Iuliu Hatieganu” University of Medicine and Pharmacy, 400006 Cluj-Napoca, Romania; chomorodean@yahoo.com; 5 Interventional Cardiology Department, Cluj County Emergency Hospital, 400006 Cluj-Napoca, Romania; 6 Association for Excellence in Pharmaceutical Education and Research, 550169 Sibiu, Romania; a.adriana.chis@gmail.com

**Keywords:** GLP-1 receptor agonists, semaglutide, tirzepatide, liraglutide, adverse drug reactions, pharmacovigilance, disproportionality analysis, Bayesian method, EudraVigilance

## Abstract

**Background:** With the increasing widespread use of GLP-1 RA and dual GIP/GLP-1 RAs in the treatment of obesity, their safety profile remains a concern for healthcare professionals (HPs). **Objective:** This study aimed to characterize and evaluate safety data from the EudraVigilance (EV) database for semaglutide (SEM), liraglutide (LIR), and tirzepatide (TIR). **Methods:** A hierarchical pharmacovigilance approach was applied, integrating SOC- and PT-level analyses with SmPC-based evaluation and both frequentist (ROR, 95% CI) and Bayesian (IC025) disproportionality methods. Within each molecule, reporter type–stratified analyses were performed, while across all molecules, disproportionality analyses were conducted separately in HP reports and in the full database to identify reporting patterns and potential safety signals, including those not described in the SmPCs. **Results:** Some ADRs, listed in the SmPC of only one or two of the three GLP1-RAs were also reported in the EV database for the other agents whose SmPCs do not specify these ADRs including optic ischemic neuropathy (TIR: 0.28% and LIR: 0.17%), alopecia (LIR: 0.81%), headache (TIR: 2.51%), intestinal obstruction (TIR: 1.55%), angioedema (LIR: 0.19%), hypersensitivity (SEM: 0.58% and LIR: 0.73%), etc. Pancreatitis, in particular, showed a significant but low-magnitude signal, being more frequently reported by HPs compared with non-HPs across all three GLP1-RAs. Additionally, statistically significant signals (IC025 > 0) were observed in both the HPs and full datasets. For example, for SEM vs. TIR, signals were identified for optic ischemic neuropathy (0.17; 0.13), gallbladder disorder (0.09; 0.11), and dysesthesia (0.42; 0.43), respectively. For TIR vs. SEM, signals were observed for injection site erythema (0.05; 0.11), injection site pruritus (0.01; 0.11), and injection site reaction (0.02; 0.08). **Conclusions:** These findings suggest potential safety signals beyond current SmPC information, emphasizing the need for continuous pharmacovigilance and cautious interpretation of reporting biases.

## 1. Introduction

Pharmacotherapy provides a nonsurgical option to reduce body weight for adults with obesity. Among the six drugs that have the treatment of obesity as one of the approved indications in the EU (liraglutide (LIR), semaglutide (SEM), tirzepatide (TIR), naltrexone/bupropion, orlistat, and setmelanotide), long-acting incretin-based therapies (LIR, SEM, and TIR) have shown greater efficacy than older anti-obesity medications [1,2].

Alongside their increasing use and popularity—reflected by Google Trends demonstrating rising search interest for terms such as SEM, TIR, and “weight loss” [3]—concerns regarding adverse drug reactions (ADRs) have also grown. A better understanding of their safety profiles—including similarities and differences between agents—may contribute to the prevention and early recognition of potential ADRs. A recent systematic review of clinical trials with anti-obesity drugs found higher rates of drug discontinuation due to ADRs with naltrexone/bupropion, GLP1-RAs, and orlistat than with other anti-obesity drugs [4,5].

While gastrointestinal symptoms remain the most frequently documented ADRs in clinical trials and post-marketing surveillance for all three GLP-1RAs, several reports and pharmacovigilance studies have described ADRs such as hair loss events [6,7,8], or non-arteritic anterior ischemic optic neuropathy (NAION), to name a few, in patients treated with SEM, LIR, and TIR, although these events are not consistently listed in the SmPC of all agents [9,10].

Although these events may be infrequent, some may be serious, and even when non-serious, they may raise concerns due to their potential impact on treatment adherence and patient quality of life. An important question is whether certain ADRs reflect potentially shared safety concerns across all agents or molecule specific reporting patterns [11]. This distinction may have clinical relevance, as it may contribute to improved awareness of potential safety concerns in patients with different comorbidity profiles.

Pharmacovigilance helps identify drug-related safety issues and is an essential component of clinical practice promoting safe medication use through the prevention, identification, analysis, management, and documentation of ADRs and other drug-related problems. It assists regulatory agencies or manufacturers in making decisions regarding withdrawal, restrictions on use, or changes to drug labels [12].

The aim of the present study was to analyze aggregated safety case data from the EudraVigilance (EV) database for GLP-1 RAs used to treat obesity (LIR, SEM, and TIR). In this respect, the ADRs reported for each drug in the Summary of Product Characteristics (SmPCs) were identified and compared to evaluate similarities and differences among the three molecules. First, a case-level assessment at the System Organ Class (SOC) level was performed, including Individual Case Safety Report (ICSR) analysis and evaluation of serious versus non-serious cases. The study also aimed to conduct a disproportionality analysis of PTs corresponding to SmPC-listed ADRs, stratified by reporter type. Furthermore, an additional objective was to explore potential similarities and differences in ADR reporting patterns among the three GLP-1 RAs. Thus, disproportionality analyses were conducted to detect potential safety signals associated predominantly with individual molecules, including signals not currently listed in the SmPCs of the other GLP-1 RAs. Therefore, by highlighting the distinct profiles of each agent, this study may contribute to the ongoing characterization of the safety profiles of GLP-1 RAs used in the management of obesity.

## 2. Results

### 2.1. Analysis of Summaries of Product Characteristics (SmPCs)

By analyzing SmPCs for the three drugs, a significant number of common ADRs were identified (e.g., anaphylactic reaction, dizziness, dysgeusia, fatigue, nausea, diarrhea, vomiting, constipation, gastroesophageal reflux disease, pancreatitis/acute pancreatitis, cholelithiasis, delayed gastric emptying, lipase increased, amylase increased, etc.). Additionally, an important number of LIR-specific ADRs are noticed (e.g., insomnia, dehydration, dry mouth, cutaneous amyloidosis, acute renal failure, renal impairment, tachycardia, etc.). For SEM, orthostatic hypotension, and NAION were listed in the SmPC, while for TIR, a single specific ADR was listed (blood calcitonin increased). On the other hand, the newest molecules (SEM and TIR) are associated with hair loss, dysesthesia, and hypotension. SEM and LIR share common ADRs, such as headache, gastritis, and intestinal obstruction, whereas TIR and LIR share only cholecystitis (Supplementary Table S2).

### 2.2. Descriptive Analysis of General Characteristics

**Figure 1:**
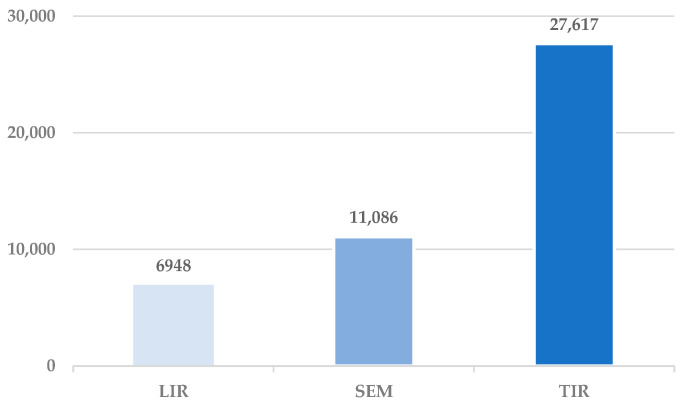
Total Individual Case Safety Reports from the EudraVigilance database submitted up to 8 February 2026.

Regarding the total number of reports, there is substantial dispersion among Saxenda^®^ (LIR) (n = 6948), Wegovy^®^ (SEM) (n = 11,086), and Mounjaro^®^ (TIR) (n = 27,617) (Figure 1). The higher number of reports recorded for TIR may be partly explained by the inclusion of ICSRs from both obesity and type 2 diabetes indications, whereas for SEM and LIR, only obesity-related ICSRs were considered.

In terms of the distribution of cases by age group, the highest number of reports was recorded in the 18–64 age group. In this age category, TIR had the highest number of ICSRs (n = 13,305, 48.20%), while LIR ranked first in terms of the proportion of reports (n = 3629, 52.23%). Reports involving patients aged 65–85 years accounted for a relatively small proportion, ranging from 5.77% (LIR) to 11.10% (TIR). In contrast, approximately 40% of reports—an unexpectedly high proportion—were categorized as “age not specified” across all three drugs (Table 1).

Regarding gender, a clear female predominance was observed for all three drugs, with the highest proportion of reported cases for LIR (78.91%, n = 5483) and the highest absolute number of cases for TIR (63.40%, n = 17,510) (Supplementary Figure S1). Regarding the origin of reports, a significant difference emerged. Specifically, the majority of reported cases involving TIR (68.20%) originated from countries outside the EEA, whereas for LIR (57.17%) and SEM (51.49%), the majority of reports came from the EEA (Supplementary Figure S2). On the other hand, HPs were the main reporter category for reporting ADRs for all three drugs (SEM—54.82%, LIR—57.87%, and TIR—61.10%) (Supplementary Figure S3).

### 2.3. The Assessment at the System Organ Class (SOC) Level

#### 2.3.1. Case Analysis by SOC

Across the five SOCs with the highest number of reported cases, a similar distribution pattern was observed, with TIR consistently reporting the highest number of cases, followed by SEM and LIR in each SOC: “Gastrointestinal disorders” (TIR: 13,074, SEM: 4904, and LIR: 3024), “General disorders and administration site conditions” (TIR: 5699, SEM: 2428, and LIR: 2026), “Injury, poisoning and procedural complications” (TIR: 3974, SEM: 1827, and LIR: 864), “Nervous system disorders” (TIR: 3495, SEM: 1684, and LIR: 924), and “Metabolism and nutrition disorder” (TIR: 3307, SEM: 1297, and LIR: 777) (Table 1).

#### 2.3.2. Analysis of Serious Cases by SOC

Although the above five mentioned SOCs accounted for the highest absolute number of reports, the proportion of serious cases within these SOCs was lower compared with other SOCs, such as “Hepatobiliary disorders” (e.g., TIR 97.4%, SEM 93.2%, LIR 90.8%), “Renal and urinary disorders” (TIR 94.5%, SEM 88.0%, LIR 83.1%), or “Blood and lymphatic system disorders” (TIR 89.9%, SEM 83.6%, LIR 80.5%). However, TIR remained the top-ranked molecule in these situations as well, followed by SEM and LIR (Table 2).

#### 2.3.3. Disproportionality Analysis of Serious Cases by SOC

The disproportionality indicators (ROR and IC) for serious versus non-serious cases are presented in Supplementary Table S4. Additionally, a heatmap (Table 3) was generated to facilitate the visualization of significant disproportionality signals.

Several significant disproportionality signals for serious cases associated with TIR compared with LIR were identified at the SOC level, including “Gastrointestinal disorders” (ROR 2.92, 95% CI: 2.69–3.17, IC025 0.04), “General disorders and administration site conditions” (ROR 3.14, 95% CI: 2.81–3.51, IC025 0.11), “Metabolism and nutrition disorders” (ROR 6.61, 95% CI: 5.58–7.83, IC025 0.07), “Nervous system disorders” (ROR 3.28, 95% CI: 2.82–3.81, IC025 0.03), and “Product issues” (ROR 14.41, 95% CI: 8.11–25.57, IC025 0.35). As can be observed, the SOC “Product issues” (IC025: 0.35) showed the highest disproportionality (ROR: 14.41) and the strongest signal robustness (IC025: 0.35) among the analyzed events, while the other SOCs demonstrated modest to low-intensity signals, with IC025 ≤ 0.11 (Supplementary Table S4).

In all other analyzed SOCs (cardiac, eye, hepatobiliary, psychiatric, renal and urinary, respiratory, thoracic and mediastinal, and skin and subcutaneous tissue disorders), IC025 values were below zero, indicating no statistically significant signal according to the Bayesian approach, despite ROR values exceeding 2 (Table 3). This discrepancy suggests that the observed disproportionality based on ROR values does not reach the robustness required for signal detection due to the negative values of IC025, which are associated with inhomogeneity, low non-serious cases, or reporting bias, such as under-reporting in the control group or geographical or temporal variability in reporting. However, these findings should be interpreted with caution.

Similar results were observed comparing TIR with SEM. While the SOC “Product issues” showed a significant disproportionality signal (ROR 14.33, 95% CI: 8.44–24.32, and IC025 0.55), other SOCs, including “Gastrointestinal disorders” (ROR 2.40, 95% CI: 2.23–2.57, IC025 0.05), “Metabolism and nutrition disorders” (ROR 4.65, 95% CI: 4.03–5.36, IC025 0.08), and “Nervous system disorders” (ROR 2.56, 95% CI: 2.26–2.90, IC025 0.04), demonstrated statistically significant but low-intensity signals. Additionally, a significant but low-intensity signal was also observed for “Skin and subcutaneous tissue disorders” (ROR 2.90, 95% CI: 2.46–3.43, IC025 0.09) (Supplementary Table S4).

Finally, the disproportionality analysis comparing SEM and LIR identified a single statistically significant, low-intensity signal for “General disorders and administration site conditions” (ROR 2.03, 95% CI: 1.78–2.30, IC025 0.09) (Supplementary Table S4). According to the established criteria, no disproportionality signals were observed by comparison of SEM with TIR, LIR with SEM, and LIR with TIR (Table 3).

### 2.4. Signal Detection at the Preferred Terms (PTs) Level

#### 2.4.1. Analysis of PTs Labeled into Summaries of Product Characteristics (SmPCs)

From the total PTs identified in the SmPCs (n = 101), 32 PTs were excluded because of a lack of reports. The three most frequently reported ADRs for all three GLP-1 RAs included (Supplementary Material—Table S3):

(1) nausea: TIR: 3660 (13.25%), SEM: 1833 (16.53%), and LIR: 1046 (15.05%),
(2) vomiting: TIR: 3315 (12.00%), SEM: 1315 (11.86%), and LIR: 611 (8.79%),
(3) diarrhea: TIR: 3194 (11.57%), SEM: 1017 (9.17%), and LIR: 501 (7.21%).

Regarding the specifically labeled ADRs, several observations can be made (Supplementary Material—Table S3):

Angioedema (n = 13, 0.19%), alopecia (n = 56, 0.81%), and hypotension (n = 27, 0.53%), which are specific ADRs for SEM and TIR, were also reported for LIR.

Optic ischemic neuropathy, an ADR SEM-specific, was also reported for TIR (n = 78, 0.28%) and LIR (n = 12, 0.17%).

Hypersensitivity, listed only in the SmPC of TIR, was also reported for SEM (n = 64, 0.58%) and LIR (n = 51, 0.73%).

A few ADRs labeled only for LIR were also listed for TIR and SEM: dry mouth, asthenia, malaise, urticaria, dehydration, insomnia, renal failure/renal impairment, and rash.

Gastritis (n = 159, 0.58%), intestinal obstruction (n = 429, 1.55%), and headache (n = 694, 2.51%), labeled only for SEM and LIR, were reported with a frequency higher than 1:1000 for TIR.

Cholecystitis (n = 95, 0.86%) and cholecystitis acute (n = 33, 0.30%), ADRs labeled for TIR and LIR, were also reported for SEM.

#### 2.4.2. Reporter Type–Stratified Disproportionality Analysis Within Each GLP-1 RA

The disproportionality indicators (ROR and IC) comparing HPs and non-HPs reports for TIR, SEM, and LIR are presented in Supplementary Tables S5–S7, respectively.

Among all PTs identified across the SmPCs of the three GLP-1 RAs, the disproportionality analysis for TIR showed higher reporting frequencies by HPs versus non-HPs for several ADRs, particularly “Pancreatitis” “Injection site reaction”, “Cholelithiasis”, “Cholecystitis”, “Cholecystitis acute”, “Anaphylactic reaction”, “Amylase increased”, “Lipase increased”, and “Angioedema”. However, a significant signal was identified only for “Lipase increased” (ROR: 3.85, 95% CI: 2.74–5.41, IC025: 0.06) and “Pancreatitis” (ROR: 1.53, 95% CI: 1.38–1.70, IC025: 0.02), both of which were of low magnitude. All these ADRs were listed in the TIR SmPCs (Supplementary Material—Table S5).

For SEM, higher reporting frequencies by HPs compared with non-HPs were observed for several ADRs, including “Optic ischemic neuropathy”, “Abdominal pain”, “Pancreatitis”, “Pancreatitis acute”, “Amylase increased”, “Lipase increased”, “Dysesthesia”, “Angioedema”, “Cholecystitis”, “Cholecystitis acute”, “Cholelithiasis”, and “Urticaria”. The last three were not listed in the SmPCs of SEM approved for obesity treatment (Wegovy^®^). However, only a single statistically significant but low-magnitude signal was identified for “Pancreatitis” (ROR: 2.57, 95%CI: 2.03–3.25, IC025 0.08) (Supplementary Material—Table S6).

For LIR, higher reporting frequencies by HPs compared with non-HPs were observed for several ADRs consistent with those listed in the SmPC, including “Pancreatitis”, “Pancreatitis acute”, “Injection site erythema”, “Injection site reaction”, “Cholecystitis”, “Cholelithiasis”, “Lipase increased”, and “Urticaria”. Similar to TIR and SEM, “Pancreatitis” demonstrated a significant but low-magnitude signal according to our results (ROR: 2.63, 95% CI: 2.11–3.27, IC025: 0.09) (Supplementary Material—Table S7).

#### 2.4.3. Disproportionality Analysis Across All Molecules Using the HP Reports and the Full Database

The disproportionality indicators (ROR and IC) for ADRs listed in the SmPCs of LIR, SEM, and TIR are presented in Supplementary Table S8 (HP reports) and in Supplementary Table S9 (overall dataset: HP and non-HP reports). Additionally, a heatmap (Table 4) was generated to improve the visualization of significant disproportionality signals.

According to data presented in Supplementary Tables S8 and S9, “Optic ischemic neuropathy”, a specific ADR for SEM, was reported more frequently for SEM than for TIR by HPs (ROR 3.23, 95% CI: 2.22–4.68, IC025 0.17), as well as by all reporters (HPs and non-HPs) (ROR 2.69, 95% CI: 1.96–3.70, IC025 0.13).

For PTs within the SOC “Gastrointestinal disorders”, statistically significant signals were observed for nausea with SEM compared with TIR, as well as for pancreatitis with LIR compared with SEM, both in HP reports and in the overall dataset (Table 4). Statistically significant but low-intensity disproportionality signals because of the lower value of IC025 were observed for nausea associated with LIR compared with TIR (ROR 1.27, 95% CI: 1.17–1.38, IC025 0.01), pancreatitis associated with TIR compared with SEM (ROR 1.79, 95% CI: 1.59–2.00, IC025 0.02), vomiting reported for SEM compared with LIR (ROR 1.53, 95% CI: 1.38–1.71, IC025 0.01), and impaired gastric emptying for TIR compared with LIR (ROR 3.37, 95% CI: 2.68–4.26, IC025 0.01) and for SEM compared with LIR (ROR 2.81, 95% CI: 2.19–3.61, IC025 0.06), based on all reports (Supplementary Table S9). All these ADRs were reported in the SmPCs of all three molecules.

On the other hand, in the SOC “General disorders and administration site conditions”, fatigue was more frequently reported for SEM than for TIR, in both HP reports and the overall dataset. Moreover, injection site erythema, pruritus, and injection site urticaria were identified in both groups (total reports and HPs), with a statistically significant higher reporting for LIR compared with TIR and SEM; in addition, injection site urticaria, was more frequently reported for TIR relative to SEM (Table 4). A significant but low-magnitude signal was noticed for TIR in both datasets for: injection site erythema (HPs: ROR 7.43, 95% CI: 4.68–11.79, IC025 0.05; HPs + non-HPs: ROR 7.12, 95% CI: 4.99–10.17, IC025 0.11), injection site pruritus (HPs: ROR 13.73, 95% CI: 6.48–29.10, IC025 0.01; HPs + non-HPs: ROR 14.47, 95% CI: 8.15–25.69, IC025 0.11), and injection site reaction (HPs: ROR 24.23, 95% CI: 9.02–65.06, IC025 0.02; HPs + non-HPs: ROR 25.37, 95% CI: 10.49–61.38, IC025 0.08).

Within the SOC “Hepatobiliary disorders”, gallbladder disorder was reported with a higher probability for SEM than for TIR by both datasets (HPs: ROR 2.93, 95% CI: 2.03–4.23, IC025 0.09; HPs + non-HPs: ROR 2.24, 95% CI: 1.73–2.90, IC025 0.11) (Supplementary Tables S8 and S9), while cholelithiasis was reported more often for LIR than for SEM when all reports were considered (ROR 2.17, 95% CI: 1.81–2.60, IC025 0.17) (Supplementary Table S9). However, the identified signals appeared weak to modest, despite statistically significant disproportionality.

Regarding nervous system disorders, a relatively strong disproportionality signal was observed for dysesthesia associated with SEM compared with TIR, in both the full reports (ROR 8.32, 95% CI: 4.44–15.60, IC025 0.43) and reports submitted by HPs (ROR 10.47, 95% CI: 5.02–21.85, IC025 0.42). Additionally, headaches were more likely to be associated with SEM or LIR than with TIR across all reports (Supplementary Table S9).

Finally, alopecia was more frequently reported for SEM than for LIR (ROR 3.20, 95% CI: 2.39–4.28, IC025 0.06) or TIR (ROR 1.58, 95% CI: 1.35–1.84, IC025 0.04), according to all reports (Supplementary Table S9), although the signal was of low intensity. A graphical representation of PTs with significant and non-significant signals is presented in Table 4.

## 3. Discussion

Pharmaceutical sales estimates for 2025 showed that Mounjaro^®^, Eli Lilly (TIR), which is approved in the EU for the treatment of both obesity and diabetes at the same doses, was the world’s second best-selling drug, while Wegovy^®^, NovoNordisk (SEM) ranked 9th [13]. Hence, the higher number of reports recorded in EV for TIR (Mounjaro^®^, Eli Lilly), compared to SEM (Wegovy^®^ and Wegovy^®^ KwikPen, NovoNordisk) and LIR (Saxenda^®^, NovoNordisk), which are approved only for the treatment of obesity, is therefore not particularly surprising. Moreover, the higher number of reports may be explained by the tendency to report ADRs more frequently for newer drugs, either due to increased caution or more intensive scientific and clinical monitoring, particularly by HPs, who represent the main category of reporters for all three drugs.

Regarding gender, a clear female predominance was observed for all three drugs, in particular for LIR (78.91%, n = 5483). This is consistent with multiple studies showing that LIR for obesity (Saxenda^®^ 3.0 mg) is used more by women than men, both in clinical trials and real-world prescribing [14,15]. Furthermore, these findings are consistent with electronic health records from various healthcare systems and with clinical trials evaluating anti-obesity medications, which have shown that participants with obesity are more often women than men [16,17]. In general, men consider weight management to be less important and believe they can control their weight on their own [18]. Females, thus, represent 65–80% of patients seeking pharmacotherapy and bariatric surgery [19,20,21]. Moreover, this also aligns with the previous literature showing that women report more ADRs than men [22,23].

The predominance of reports on TIR from outside the EEA in EV may reflect the earlier and more widespread use of this drug outside the EEA, particularly in the US, combined with regulatory requirements that stipulate that marketing authorization holders report ADRs globally. By contrast, LIR and SEM have been widely used in the EEA for some time, which may explain the higher proportion of reports originating from the EEA. Therefore, these differences reflect variations in medicine exposure and prescribing practices—which might vary according to availability, government policies on reimbursement, physician preferences, and timing of market introduction—as well as reporting practices rather than actual differences in safety profiles.

Not at all unexpected, the highest number of reported cases was observed in the SOC “Gastrointestinal disorders” for each drug, which is in line with most clinical trials (e.g., SURPASS and SURMOUNT studies), post-marketing surveillance, and the SmPCs [24,25,26,27,28,29,30,31,32,33,34,35,36,37,38,39,40].

Across the top five SOCs with the highest number of ICSRs: “Gastrointestinal disorders”, “General disorders and administration site conditions”, “Injury, poisoning, and procedural complications”, “Nervous system disorders”, and “Metabolism and nutrition disorders”, TIR had the most reports, followed by SEM and then LIR. In fact, this hierarchical pattern: TIR > SEM > LIR has been noted in almost all SOCs, with a few exceptions.

However, the proportion of serious cases was higher in other SOCs, namely “Hepatobiliary disorders”, “Renal and urinary disorders”, and “Blood and lymphatic system disorders”, although the same ranking remained: TIR with the highest number of reports, followed by SEM and LIR. Importantly, the disproportionality of serious cases with TIR in “Metabolism and nutrition disorders”, “Gastrointestinal disorders”, “Nervous system disorders”, or “Renal and urinary disorders” was not increased compared with SEM and LIR, as indicated by the low IC and IC025 values. Another study evaluating trends in ADRs (both serious and non-serious) reported in the EV database found that, among the six currently approved anti-obesity drugs (orlistat, naltrexone/bupropion, setmelanotide, SEM, LIR, and TIR), only SEM demonstrates a trend of a continuous increase in annual reports of serious ADRs. Further investigation of these safety signals, therefore, appears necessary [4].

On the contrary, we observed that serious cases associated with “Product issues” were reported significantly more frequently with TIR as compared to SEM and LIR. This finding might be explained by product-related factors such as supply shortages leading to treatment interruption, recalls due to contamination, device/pen defects (dose selector malfunction), falsified products, or issues related to sterility and compounded formulations, cited in the literature not only with TIR but also with the other two obesity approved GLP-1 RAs, rather than intrinsic pharmacological effects [41,42,43,44]. For example, impurities resulting from chemical interactions between TIR and vitamin B12 have been documented in compound products, which are, however, not FDA-approved [43,45]. On the other hand, in the United Kingdom, counterfeit 15 mg KwikPen pens with defective dose-adjustment mechanisms have been identified [46]. However, such observations should be interpreted with caution, as spontaneous reporting systems do not allow for the establishment of a causal link.

All other SOCs demonstrated only low-intensity disproportionality signals, with the highest observed for “General disorders and administration site conditions”, with TIR compared with LIR and for “Skin and subcutaneous tissue disorders”, with TIR compared with SEM, indicating limited strength of association.

The top three most common gastrointestinal reports were nausea, diarrhea, vomiting for each drug, consistent with their respective package leaflet. SEM showed the highest proportion of nausea among the three agents. This pattern was supported by disproportionality analysis, which identified a statistically significantly higher reporting of nausea for SEM compared with TIR. The results in the literature, including SURMONT-5, are mixed—some studies found no significant differences in the incidence of gastrointestinal ADRs between TIR and SEM, while others observed a difference favoring either SEM or TIR [29,31,47,48,49].

Some ADRs, namely angioedema, alopecia, hypotension, NAION, hypersensitivity, dry mouth, asthenia, malaise, urticaria, dehydration, insomnia, renal failure/renal impairment and rash, cholecystitis and cholecystitis acute, gastritis, intestinal obstruction, and headache, listed in the SmPC of only one or two of the three GLP1-RAs were also reported in the EV database for the other agents that do not include these ADRs in their SmPCs. These findings may suggest shared reporting patterns or potential emerging safety signals requiring further investigation. Moreover, gastritis, intestinal obstruction, and headache, not listed in the SmPC of TIR, were reported for this agent with a frequency exceeding 1:1000. These observations are in line with findings from FAERS-based analyses [29]. However, headache appeared to be more frequently reported with SEM and LIR compared with TIR in the overall dataset.

Rare cases of serious gastrointestinal events, including intestinal obstruction, were also reported in SURMOUNT trials [50] and in a couple of case studies [51,52,53]. When analyzing SmPC-listed PTs separately for each of the three GLP1-RAs in HP versus non-HP reports, a similar pattern was observed across all three GLP1-RAs, with “Pancreatitis” showing higher reporting by HPs compared with non-HPs, although the signal was of low intensity. The predominance of HP reports likely reflects the clinical severity of the adverse event and the need for objective diagnostic confirmation. The diagnosis is suggested by clinical features, namely, severe and persistent epigastric pain that can radiate to the back and confirmed by elevated serum lipase or amylase activity (≥3 times the upper limit of normal) and characteristic imaging findings on CT or MRI. However, the low signal intensity suggests that this finding is more likely driven by reporting patterns rather than a strong underlying association. LIR used in weight management can cause acute pancreatitis as a rare complication [54]. This observation is consistent with several case reports and literature review [55,56]. Interestingly, statistically significant signals for pancreatitis were observed for LIR compared with SEM, both in HP reports and in the overall dataset, and this is in line with findings from a real-world pharmacovigilance analysis of the FAERS database. Across different statistical measures, LIR demonstrated the strongest association with acute pancreatitis among all GLP-1 RAs. Following LIR, exenatide, SEM, dulaglutide, lixisenatide, and albiglutide exhibited progressively lower values, while TIR demonstrated the lowest association [57]. Risk factors that may predispose to pancreatitis development while on LIR might include concomitant use of medications such as metformin and cholelithiasis. Moreover, LIR may induce expansion of the exocrine pancreas [58], with ductal proliferation and metaplasia reported as mechanisms for pancreatitis in humans [59,60]. However, another meta-analysis concludes that GLP-1 RAs carry a slightly increased risk of pancreatitis, which is not significant when stratified by background medication use [61]. Still, awareness of this potential adverse event helps with early diagnosis and discontinuation of the causative medication, in order to prevent complications and recurrence [55].

Another PT within the SOC “Gastrointestinal disorders” that deserves attention is “Impaired gastric emptying”, which can lead to gastroparesis and which showed higher reporting for TIR and SEM compared with LIR in the full dataset. All GLP-1-based therapies delay gastric emptying, because their therapeutic mechanisms are, at least in part, mediated by their influence on gastric emptying, although the effect appears to be more pronounced with short-acting agents, as compared to long-acting compounds. In case of long-acting GLP1-RAs including TIR, SEM, and LIR, the magnitude of this effect is variable and tends to diminish with prolonged treatment, suggesting tachyphylaxis [62,63,64,65]. Another important aspect of delayed gastric emptying associated with TIR and SEM is that patients may have diabetic gastroparesis at baseline or other pre-existing gastrointestinal comorbidities, which may predispose them to the development or worsening of gastroparesis [64,66].

On the other hand, SEM had lower rates of gastroparesis (3.6%) compared to exenatide (6.9%) and LIR (5.9%) in a retrospective cross-sectional analysis that used real-world data on 10,328 adults with diabetes/obesity in the National Institutes of Health All of Us cohort [63]. This delayed gastric emptying poses a risk, particularly when performing esophagogastroduodenoscopy, and may require specific precautions during the induction of anesthesia to prevent pulmonary aspiration of gastric contents [67]. It may also affect the absorption of concomitantly administered oral drugs [68]; therefore, prescribing physicians should be aware of these potential side effects [64].

Cholelithiasis is an ADR described in the SmPC of all three GLP-1 RAs. Several RCTs have demonstrated a higher rate of gallbladder disorders in patients who were randomized to GLP-1 RAs vs. placebo [37,69,70,71]. Both dose and duration of treatment play a key role in determining the risk of cholelithiasis, with longer treatment duration and higher doses correlating with a higher likelihood of gallstone formation [70,72]. We noted that the PT “gallbladder disorder” demonstrated higher reporting for SEM compared with TIR in both datasets, while “cholelithiasis” was more frequently reported for LIR than for SEM when all reports were considered, although the identified signals appeared weak to modest. In meta-analysis SEM showed a statistically significant increased risk of cholelithiasis and gallbladder disease compared to controls whereas TIR did not show significant biliary risk when comparing to placebo [73]. On the other hand, another meta-analysis of 43 trials (~39,000 patients) found that LIR, in particular, was associated with an increased incidence of cholelithiasis in subgroup analyses across GLP-1 receptor agonists (exenatide, LIR, lixisenatide, albiglutide, dulaglutide, and SEM) [74].

Although not described in the SmPCs of TIR and LIR, ischemic optic neuropathy has been reported with both agents, warranting further attention. However, it showed higher reporting for SEM than for TIR, although the signal strength was weak-to-moderate, while no difference was observed when comparing SEM with LIR. GLP-1 receptors are expressed in the optic nerve [75] and, therefore, may be directly affected by GLP-1 RAs [76]. NAION is an acute disorder of the optic nerve that results in sudden, painless vision loss. Multiple studies suggest a potential association between TIR and NAION/ischemic optic neuropathy, supported by pharmacovigilance data, a large cohort study, and case reports [10,76,77]. Possible contributing mechanisms include sudden metabolic shifts such as rapid glycemic and blood pressure correction that may lead to optic nerve ischemia, particularly in individuals with pre-existing vascular risk factors [10]. The event is rare but serious and warrants cautious clinical consideration and further investigation. Close monitoring of optic nerve disorders in patients starting GLP1-RAs should be recommended.

Dysesthesia is listed in the SmPCs of both TIR and SEM. Consistent with the literature in the field [78,79], our study found higher reporting for SEM compared with TIR. Moreover, dysesthesia rates appear to increase with higher doses of SEM [80].

Regarding PTs within the SOC “General disorders and administration site conditions injection” injection site erythema, injection site pruritus, and injection site urticaria showed a statistically significant higher reporting for LIR compared with both TIR and SEM. Injection site reactions and skin ADRs to LIR have been rarely reported in the literature; however, they can be severe and may lead to discontinuation of treatment [81,82,83]. Although much more data is needed in this regard, SEM might represent a safe therapeutic alternative in patients with allergy to LIR [81].

Last but not least, a higher reporting of alopecia associated with SEM was observed compared with TIR and LIR when all reports were taken into account, although the signal was of low intensity. Alopecia (hair loss) has been reported as a potentially emerging ADRs associated with GLP1-RAs. Several mechanisms have been proposed, including potential effects of GLP-1 RAs on the hair follicle cycle, together with metabolic and nutritional changes associated with rapid and substantial weight loss [78,84]. A disproportionality analysis of FAERS data (2022–2023) identified a statistically significant association between the use of SEM (ROR: 2.46, 95% CI: 2.14–2.83) and TIR (ROR: 1.73, 95% CI: 1.42–2.09) with alopecia [85]. Although a causal relationship cannot be confirmed and no well-defined pathophysiological mechanisms have been established, alopecia was consistently reported as an ADR across various clinical contexts and GLP-1RA agents [8]. Further research to clarify the relationship between GLP-1RAs and alopecia is warranted. Meanwhile, being aware of this potential effect can improve treatment adherence and prevent unnecessary diagnostic procedures [8].

One aspect that should not be overlooked is the influence of media attention. Certain ADRs, particularly NAION [86], alopecia [7,87], and pancreatitis [88], have recently received great attention from both the media and regulatory authorities, potentially contributing to stimulated reporting and notoriety bias, particularly for newer drugs.

### Limitations of the Study

Our study has several limitations, which can influence the interpretation of the results and the generalization of the conclusions. One key limitation is related to the nature of the EV database, a spontaneous and voluntary reporting system, which can generate selective, incomplete, or inaccurate reports, thus introducing potential biases that are difficult to control. Additionally, indication heterogeneity represents another major shortcoming, as TIR reports may include both obesity and type 2 diabetes cases, whereas SEM and LIR datasets appear restricted primarily to obesity indications, potentially introducing confounding in comparisons across molecules. A further limitation concerns the age categories available in EV, which are predefined and unevenly distributed. In particular, the 18–64 age group covers a much broader range than the other categories, potentially creating numerical imbalance. Furthermore, the reported frequencies do not reflect true event rates and may be influenced by market penetration, prescription volume, duration of marketing, and stimulated reporting.

Under these circumstances, the evaluation of clinically relevant factors, such as the dose administered, duration of treatment, comorbidities, drug combinations or other parameters influencing the occurrence of adverse reactions, becomes limited or even impossible. In addition, because the system is based on spontaneous reports, the quality and completeness of the data cannot be guaranteed, and the total population exposed to GLP-1 RAs cannot be accurately determined. Another important limitation is the phenomenon of under-reporting, which may lead to an underestimation of the true incidence of adverse reactions. Furthermore, the lack of adjustment for multiple testing and the absence of predefined signal prioritization criteria represent additional shortcomings, as they may increase the risk of false-positive findings in the context of multiple exploratory disproportionality analyses. Despite the mentioned limitations, the analysis of data from EV still provides a valuable overview and an effective method for monitoring the safety of the post-marketing drug.

## 4. Materials and Methods

### 4.1. Study Design

The present study focused on two GLP-1 RAs and one dual GLP-1/GIP receptor agonist, all used in the treatment of obesity, reflecting the growing interest in these therapies. Tirzepatide (Mounjaro^®^) is approved for both obesity and type 2 diabetes, whereas semaglutide (Wegovy^®^) and liraglutide (Saxenda^®^) are specifically approved for obesity, while alternative formulations of the same active substances are indicated for diabetes. The first step of the study involved identifying the common and specific ADRs across the three GLP-1 RAs by comparing the SmPC of brands approved for obesity treatment (Supplementary Table S2). Following, a descriptive and disproportionality analysis of aggregated data submitted in the EV for TIR, SEM, and LIR up to 8 February 2026 was performed. Data were extracted from the EV portal (addreports.eu), between 10 and 12 February 2026. Because products containing TIR were approved in the European Union (EU) under the same brand name for both obesity and diabetes treatment, and because EudraVigilance does not systematically allow stratification by indication at the individual case level, aggregated data reported for tirzepatide (active substance) were analyzed alongside those reported for SEM (Wegovy^®^ and Wegovy^®^ KwikPen) and LIR (Saxenda^®^).

Subsequently, to ensure maximum sensitivity in data extraction, based on MedDRA’s (Medical Dictionary for Regulatory Activities) multiaxial structure, the preferred terms (PTs) from all relevant SOCs used to report the labeled ADRs were identified (Supplementary Table S3). Accordingly, all data reported were analyzed even when a PT from a different SOC was not used to report an ADR in the EV database.

#### Inclusion and Exclusion Criteria

Data related to labeled ADRs in the SmPCs of the three brand names, approved in the European Union, for obesity treatment were included in the analysis, and PTs related to these ADRs were extracted.

To reduce the bias, some of the PTs used for reporting in SmPCs were excluded: (i) hypoglycemia—specific ADRs of the antidiabetic drugs; (ii) diabetic retinopathy, a specific complication of diabetes mellitus; (iii) decreased appetite or weight decrease, which are desired effects of GLP-1 RAs when they are used in obesity. Therefore, none of these ADRs was included in our analysis. On the other hand, according to regulations, drugs were excluded from the disproportionality analysis if there were fewer than three reports. Subsequently, to increase the sensitivity of the disproportionality analysis, reports submitted by non-HPs were excluded.

### 4.2. Descriptive Analysis

Descriptive analysis included a comparative evaluation of general characteristics (total number of reports, distribution of reports by age, sex, reporter, or origin), the number of cases reported within each SOC, and the proportion of serious cases (by dividing the numbers of serious and non-serious cases by the total number of cases in each SOC).

Moreover, to identify similarities and notable differences among the three GLP-1 RAs, a comparative analysis of their safety profiles was performed. First, the seriousness rates of cases reported in each SOC were compared across the three GLP-1 RAs. Subsequently, a signal detection analysis at the PT level and the frequency of PTs used for the labeled ADRs were analyzed, too.

### 4.3. Disproportionality Analysis

Based on a 2 × 2 contingency table, the signals’ disproportionality was approached for each SOC by comparing serious and non-serious cases (Supplementary Table S1). The magnitude of the relative risk of reporting ADRs in the SOC was evaluated using the reporting odds ratio (ROR) and its 95% confidence interval (CI) [89]. Because in some situations the number of cases in the non-exposed group was 0 and, implicitly, the ROR value would be 0 or ∞, a Haldane-Anscombe ½ correction was applied [90]. Thus, a 0.5 value was added in all four categories of the contingency table (Supplementary Table S1). A threshold of ROR > 2, with the lower bound of the 95% CI > 1, represents a stringent criterion for prioritizing disproportionality signals at the SOC and PT levels, provides a robust analytical framework, and helps minimize bias [91]. Also, this threshold helps filter the inherent noise in spontaneous reporting. Subsequently, the information component (IC), a robust indicator for small sample sizes, was calculated to confirm the stability of the signals. Even though both ROR and IC were used to prioritize signals, a stable safety signal was considered statistically significant if the lower bound of the 95% IC was greater than zero (IC025 > 0) [92,93,94,95].

*IC* = *log*_2_ [(*a* + 0.5)/(*E* + 0.5)]

IC = information component

E = expected number of cases

where the E:

E=[n1×m1]/N

Then, to evaluate statistical certainty, the variance (V[IC]) and the 95% CI were derived:

$$V[IC]\approx [1/{\left(ln2\right)}^{2}]\times [(1/(a+0.5))-(1/(N+0.5))]$$

Lower Bound (IC025): IC − (1.96 × √V[IC])

Upper Bound (IC975): IC + (1.96 × √V[IC])

V[IC] = variance

Following the SOC analysis, disproportionality was used to identify stable safety signals associated with PTs used to report the labeled ADRs. To improve the analysis sensitivity, a secondary disproportionality was performed, considering only the HPs’ reports. Therefore, data resulting from the total reports were compared with those resulting from HP’s reports.

### 4.4. Ethics

No ethics committee approval is required for descriptive and disproportionality analysis, because ICSRs do not contain any personal information [96].

## 5. Conclusions

Clinical trials are fundamental for the approval of new drugs and medical devices; however, they provide only a partial assessment of safety and effectiveness. Additional insights are obtained from real-world evidence and pharmacovigilance activities, including the analysis of spontaneous reports and observational data, which allow for the evaluation of therapies in broader and more heterogeneous patient populations. As the use of GLP-1 receptor agonists and TIR continues to expand, ongoing vigilance and individualized risk assessment remain essential to optimize patient safety and therapeutic outcomes. “Gastrointestinal disorders” accounted for the highest number of reports across all drugs, with TIR ranking highest, followed by SEM and LIR. Among serious cases, a significant difference was observed only for TIR in “Product issues”. Some ADRs listed in the SmPC of only one or two of the three GLP1-RAs were also reported in the EV database for all other agents that do not include these ADRs in their SmPCs. While the present disproportionality analysis cannot establish whether these findings reflect a true class effect, they might represent potential emerging safety concerns requiring further investigation. However, most identified signals were not statistically significant, and many of the statistically significant findings demonstrated only weak disproportionality, warranting cautious interpretation. Therefore, these results should primarily be regarded as hypothesis-generating rather than evidence of causal or clinically worthwhile differences between agents. Among the signals achieving statistical significance in both the HP reports and the overall dataset were pancreatitis, NAION, nausea, dysesthesia, fatigue, gallbladder disorder, and injection site reactions (erythema, pruritus, urticaria). Nevertheless, these findings should be interpreted as signal detection results and do not establish causal relationships. Future research that incorporates real-world cohort analyses, temporal analyses, exposure-adjusted analyses, comparative studies, and deeper insights into the signaling mechanisms of GLP-1 and GIP receptors could help determine whether the three GLP-1RAs generate distinct or common safety profiles or whether the observed signals primarily reflect contextual factors related to the treated populations and pharmacovigilance surveillance.

## Figures and Tables

**Table 1:** Case distribution by age category.

	LIR		SEM		TIR	
	n	%	n	%	n	%
Not Specified	2837	40.83%	4398	39.67%	11,136	40.30%
0–1 Month	3	0.04%	0	0.00%	4	0.00%
2 Months–2 Years	3	0.04%	3	0.03%	5	0.00%
3–11 Years	5	0.07%	8	0.07%	3	0.00%
12–17 Years	67	0.96%	72	0.65%	18	0.10%
18–64 Years	3629	52.23%	5764	51.99%	13,305	48.20%
65–85 Years	401	5.77%	833	7.51%	3053	11.10%
More than 85 Years	3	0.04%	8	0.07%	93	0.30%

**Table 2:** Distribution and frequency of cases by SOCs (number and rank).

SOC	Total Cases for TIR n (Rank)	Total Cases for SEM n (Rank)	Total Cases for LIR n (Rank)	Serious TIR Cases n (%)	Serious SEM Cases n (%)	Serious LIR Cases n (%)
Blood and lymphatic system disorders	267 (20)	61 (26)	41 (25)	240 (89.9%)	51 (83.6%)	33 (80.5%)
Cardiac disorders	1129 (13)	448 (13)	236 (13)	969 (85.8%)	372 (83.0%)	148 (62.7%)
Congenital, familial, and genetic disorders	42 (25)	26 (27)	21 (27)	39 (92.9%)	26 (100.0%)	21 (100.0%)
Ear and labyrinth disorders	200 (21)	104 (24)	52 (24)	151 (75.5%)	56 (53.8%)	24 (46.2%)
Endocrine disorders	181 (22)	111 (23)	74 (22)	152 (84.0%)	100 (90.1%)	59 (79.7%)
Eye disorders	1069 (14)	614 (10)	148 (19)	894 (83.6%)	460 (74.9%)	91 (61.5%)
Gastrointestinal disorders	13,074 (1)	4904 (1)	3024 (1)	10,192 (78.0%)	2923 (59.6%)	1657 (54.8%)
General disorders and administration site conditions	5699 (2)	2428 (2)	2026 (2)	3071 (53.9%)	1043 (43.0%)	549 (27.1%)
Hepatobiliary disorders	2067 (8)	663 (9)	511 (8)	2013 (97.4%)	618 (93.2%)	464 (90.8%)
Immune system disorders	635 (18)	130 (21)	98 (21)	535 (84.3%)	98 (75.4%)	65 (66.3%)
Infections and infestations	1447 (11)	459 (12)	307 (10)	1240 (85.7%)	377 (82.1%)	229 (74.6%)
Injury, poisoning, and procedural complications	3974 (3)	1827 (3)	864 (4)	2806 (70.6%)	1116 (61.1%)	544 (63.0%)
Investigations	3305 (6)	913 (8)	647 (6)	2358 (71.3%)	518 (56.7%)	345 (53.3%)
Metabolism and nutrition disorders	3307 (5)	1297 (5)	777 (5)	2762 (83.5%)	676 (52.1%)	337 (43.4%)
Musculoskeletal and connective tissue disorders	1596 (10)	541 (11)	234 (14)	1195 (74.9%)	348 (64.3%)	142 (60.7%)
Neoplasms—benign, malignant, and unspecified (incl. cysts and polyps)	714 (17)	183 (20)	241 (11)	707 (99.0%)	183 (100.0%)	240 (99.6%)
Nervous system disorders	3495 (4)	1684 (4)	924 (3)	2713 (77.6%)	969 (57.5%)	475 (51.4%)
Pregnancy, puerperium, and perinatal conditions	148 (23)	90 (25)	59 (23)	141 (95.3%)	86 (95.6%)	53 (89.8%)
Product issues	107 (24)	319 (16)	195 (15)	83 (77.6%)	61 (19.1%)	37 (19.0%)
Psychiatric disorders	2444 (7)	1235 (6)	545 (7)	2054 (84.0%)	869 (70.4%)	327 (60.0%)
Renal and urinary disorders	1151 (12)	258 (19)	189 (16)	1088 (94.5%)	227 (88.0%)	157 (83.1%)
Reproductive system and breast disorders	586 (19)	313 (17)	118 (20)	404 (68.9%)	150 (47.9%)	57 (48.3%)
Respiratory, thoracic, and mediastinal disorders	851 (16)	329 (15)	158 (18)	734 (86.3%)	241 (73.3%)	98 (62.0%)
Skin and subcutaneous tissue disorders	1885 (9)	969 (7)	490 (9)	1011 (53.6%)	276 (28.5%)	149 (30.4%)
Social circumstances	25 (26)	123 (22)	38 (26)	17 (68.0%)	106 (86.2%)	31 (81.6%)
Surgical and medical procedures	25 (26)	412 (14)	241 (11)	17 (68.0%)	410 (99.5%)	238 (98.8%)
Vascular disorders	888 (15)	308 (18)	160 (17)	774 (87.2%)	229 (74.4%)	118 (73.8%)

**Table 3:** Heatmap of significant vs. non-significant disproportionality signals. LIR—liraglutide, NS—no signal, SEM—semaglutide, SS—significant signal, TIR—tirzepatide. Green color—no signal; red color—significant signal.

SOC	LIR-TIR	SEM-TIR	LIR-SEM	TIR-LIR	TIR-SEM	SEM-LIR
Blood and lymphatic system disorders	NS	NS	NS	NS	NS	NS
Cardiac disorders	NS	NS	NS	NS	NS	NS
Congenital, familial, and genetic disorders	NS	NS	NS	NS	NS	NS
Ear and labyrinth disorders	NS	NS	NS	NS	NS	NS
Endocrine disorders	NS	NS	NS	NS	NS	NS
Eye disorders	NS	NS	NS	NS	NS	NS
Gastrointestinal disorders	NS	NS	NS	SS	SS	NS
General disorders and administration site conditions	NS	NS	NS	SS	NS	SS
Hepatobiliary disorders	NS	NS	NS	NS	NS	NS
Immune system disorders	NS	NS	NS	NS	NS	NS
Infections and infestations	NS	NS	NS	NS	NS	NS
Injury, poisoning, and procedural complications	NS	NS	NS	NS	NS	NS
Investigations	NS	NS	NS	NS	NS	NS
Metabolism and nutrition disorders	NS	NS	NS	SS	SS	NS
Musculoskeletal and connective tissue disorders	NS	NS	NS	NS	NS	NS
Neoplasms—benign, malignant, and unspecified (including cysts and polyps)	NS	NS	NS	NS	NS	NS
Nervous system disorders	NS	NS	NS	SS	SS	NS
Pregnancy, puerperium, and perinatal conditions	NS	NS	NS	NS	NS	NS
Product issues	NS	NS	NS	SS	SS	NS
Psychiatric disorders	NS	NS	NS	NS	NS	NS
Renal and urinary disorders	NS	NS	NS	NS	NS	NS
Reproductive system and breast disorders	NS	NS	NS	NS	NS	NS
Respiratory, thoracic, and mediastinal disorders	NS	NS	NS	NS	NS	NS
Skin and subcutaneous tissue disorders	NS	NS	NS	NS	SS	NS
Social circumstances	NS	NS	NS	NS	NS	NS
Surgical and medical procedures	NS	NS	NS	NS	NS	NS
Vascular disorders	NS	NS	NS	NS	NS	NS

**Table 4:** Heatmap of disproportionality signals associated with ADRs labeled in the SmPCs of liraglutide, semaglutide, and tirzepatide. HP—healthcare professionals, LIR—liraglutide, NA—not available, NS—no signal, SEM—semaglutide, SS—significant signal, TIR—tirzepatide. Green color—no significant signals, orange color—not available (the significance could not be calculated because of the lower number of reports), yellow color—significant signals only in total reports, red color—significant signals in total reports and also in HPs reports.

SOC	PT	Total Reports (HPs + Non-HPs)						HPs Reports					
		TIR-SEM	TIR-LIR	SEM-LIR	SEM-TIR	LIR-TIR	LIR-SEM	TIR-SEM	TIR-LIR	SEM-LIR	SEM-TIR	LIR-TIR	LIR-SEM
Cardiac disorders	Sinus tachycardia	NS	NS	NS	NS	NS	NS	NA	NS	NA	NA	NS	NA
	Tachycardia	NS	NS	NS	NS	NS	NS	NS	NS	NS	NS	NS	NS
Eye disorders	Optic ischemic neuropathy	NS	NS	NS	SS	NS	NS	NS	NS	NS	SS	NS	NS
Gastrointestinal disorders	Abdominal discomfort	NS	NS	NS	NS	NS	NS	NS	NS	NS	NS	NS	NS
	Abdominal distension	NS	NS	NS	NS	NS	NS	NS	NS	NS	NS	NS	NS
	Abdominal pain	NS	NS	NS	NS	NS	NS	NS	NS	NS	NS	NS	NS
	Abdominal pain lower	NS	NS	NS	NS	NS	NS	NS	NS	NS	NS	NS	NS
	Abdominal pain upper	NS	NS	NS	NS	NS	NS	NS	NS	NS	NS	NS	NS
	Constipation	NS	NS	NS	NS	NS	NS	NS	NS	NS	NS	NS	NS
	Diarrhea	NS	NS	NS	NS	NS	NS	NS	NS	NS	NS	NS	NS
	Dry mouth	NS	NS	NS	NS	NS	NS	NS	NS	NS	NS	NS	NS
	Dyspepsia	NS	NS	NS	NS	NS	NS	NS	NS	NS	NS	NS	NS
	Eructation	NS	NS	NS	NS	NS	NS	NS	NS	NS	NS	NS	NS
	Flatulence	NS	NS	NS	NS	NS	NS	NS	NS	NS	NS	NS	NS
	Gastritis	NS	NS	NS	NS	NS	NS	NS	NS	NS	NS	NS	NS
	Gastroesophageal reflux disease	NS	NS	NS	NS	NS	NS	NS	NS	NS	NS	NS	NS
	Impaired gastric emptying	NS	SS	SS	NS	NS	NS	NS	NS	NS	NS	NS	NS
	Intestinal obstruction	NS	NS	NS	NS	NS	NS	NS	NS	NS	NS	NS	NS
	Nausea	NS	NS	NS	SS	SS	NS	NS	NS	NS	SS	NS	NS
	Pancreatitis	SS	NS	NS	NS	NS	SS	NS	NS	NS	NS	NS	SS
	Pancreatitis acute	NS	NS	NS	NS	NS	NS	NS	NS	NS	NS	NS	NS
	Vomiting	NS	NS	SS	NS	NS	NS	NS	NS	NS	NS	NS	NS
General disorders and administration site conditions	Asthenia	NS	NS	NS	NS	NS	NS	NS	NS	NS	NS	NS	NS
	Fatigue	NS	NS	NS	SS	NS	NS	NS	NS	NS	SS	NS	NS
	Injection site erythema	SS	NS	NS	NS	SS	SS	SS	NS	NS	NS	SS	SS
	Injection site pain	NS	NS	NS	NS	NS	NS	NS	NS	NS	NS	NS	NS
	Injection site pruritus	SS	NS	NS	NS	SS	SS	SS	NS	NS	NS	SS	SS
	Injection site reaction	SS	NS	NS	NS	NS	SS	SS	NS	NS	NS	NS	SS
	Injection site urticaria	NS	NS	NS	NS	SS	SS	NS	NS	NS	NS	SS	SS
	Malaise	NS	NS	NS	NS	NS	NS	NS	NS	NS	NS	NS	NS
Hepatobiliary disorders	Cholecystitis	NS	NS	NS	NS	NS	NS	NS	NS	NS	NS	NS	NS
	Cholecystitis acute	NS	NS	NS	NS	NS	NS	NS	NS	NS	NS	NS	NS
	Cholelithiasis	NS	NS	NS	NS	NS	SS	NS	NS	NS	NS	NS	NS
	Gallbladder disorder	NS	NS	NS	SS	NS	NS	NS	NS	NS	SS	NS	NS
Immune system disorders	Anaphylactic reaction	NS	NS	NS	NS	NS	NS	NS	NS	NS	NS	NS	NS
	Anaphylactic shock	NS	NS	NS	NS	NS	NS	NS	NS	NS	NS	NS	NS
	Hypersensitivity	NS	NS	NS	NS	NS	NS	NS	NS	NS	NS	NS	NS
Investigations	Amylase increased	NS	NS	NS	NS	NS	NS	NS	NS	NS	NS	NS	NS
	Heart rate increased	NS	NS	NS	NS	NS	NS	NS	NS	NS	NS	NS	NS
	Lipase increased	NS	NS	NS	NS	NS	NS	NS	NS	NS	NS	NS	NS
Metabolism and nutrition disorders	Dehydration	NS	NS	NS	NS	NS	NS	NS	NS	NS	NS	NS	NS
	Dizziness	NS	NS	NS	NS	NS	NS	NS	NS	NS	NS	NS	NS
	Dysesthesia	NS	NA	NA	SS	NA	NA	NS	NS	NS	SS	NS	NS
	Dysgeusia	NS	NS	NS	NS	NS	NS	NS	NS	NS	NS	NS	NS
	Headache	NS	NS	NS	SS	SS	NS	NS	NS	NS	NS	NS	NS
	Lethargy	NS	NS	NS	NS	NS	NS	NS	NS	NS	NS	NS	NS
Psychiatric disorders	Insomnia	NS	NS	NS	NS	NS	NS	NS	NS	NS	NS	NS	NS
Renal and urinary disorders	Renal failure	NS	NS	NS	NS	NS	NS	NS	NS	NS	NS	NS	NS
Skin and subcutaneous tissue disorders	Alopecia	NS	NS	SS	SS	NS	NS	NS	NS	NS	NS	NS	NS
	Angioedema	NS	NS	NS	NS	NS	NS	NS	NS	NS	NS	NS	NS
	Dermatitis allergic	NS	NS	NS	NS	NS	NS	NS	NS	NS	NS	NS	NS
	Eczema	NS	NS	NS	NS	NS	NS	NS	NA	NA	NS	NA	NA
	Erythema	NS	NS	NS	NS	NS	NS	NS	NS	NS	NS	NS	NS
	Rash	NS	NS	NS	NS	NS	NS	NS	NS	NS	NS	NS	NS
	Urticaria	NS	NS	NS	NS	NS	NS	NS	NS	NS	NS	NS	NS
Vascular disorders	Hypotension	NS	NS	NS	NS	NS	NS	NS	NS	NS	NS	NS	NS
	Orthostatic hypotension	NS	NS	NS	NS	NS	NS	NS	NS	NS	NS	NS	NS

## Data Availability

The original contributions presented in this study are included in the article/Supplementary Materials. Further inquiries can be directed to the corresponding author.

## Supplementary Materials

The following supporting information can be downloaded at: https://www.mdpi.com/article/10.3390/ph19060876/s1. References in Supplementary Materials file can be found [97,98,99,100,101,102].

## References

[1] Wong H.J., Sim B., Teo Y.H., Teo Y.N., Chan M.Y., Yeo L.L.L., Eng P.C., Tan B.Y.Q., Sattar N., Dalakoti M. (2025). Efficacy of GLP-1 Receptor Agonists on Weight Loss, BMI, and Waist Circumference for Patients With Obesity or Overweight: A Systematic Review, Meta-Analysis, and Meta-Regression of 47 Randomized Controlled Trials. Diabetes Care.

[2] Kushner R.F., Johansen O.E., Araujo Torres K., Phan T.-M., Marczewska A. (2025). Symptomatic Adverse Events and Quality of Life Related to Incretin-Based Medicines for Obesity: A Systematic Review Involving >400,000 Subjects. Obesities.

[3] Butuca A., Dobrea C.M., Arseniu A.M., Frum A., Chis A.A., Rus L.L., Ghibu S., Juncan A.M., Muntean A.C., Lazăr A.E. (2024). An Assessment of Semaglutide Safety Based on Real World Data: From Popularity to Spontaneous Reporting in EudraVigilance Database. Biomedicines.

[4] Raičević B.B., Belančić A., Mirković N., Janković S.M. (2025). Analysis of Reporting Trends of Serious Adverse Events Associated with Anti-Obesity Drugs. Pharmacol. Res. Perspect..

[5] Shi Q., Wang Y., Hao Q., Vandvik P.O., Guyatt G., Li J., Chen Z., Xu S., Shen Y., Ge L. (2024). Pharmacotherapy for Adults with Overweight and Obesity: A Systematic Review and Network Meta-Analysis of Randomised Controlled Trials. Lancet.

[6] Shand J.A.D., Young S., Verster F., Peters C. (2023). Pilot Study to Test the Safety, Tolerability and Feasibility of Dulaglutide during a Low-Energy Diet for Weight Loss and Improved Glycaemic Control. BMJ Nutr. Prev. Health.

[7] Burke O., Sa B., Cespedes D.A., Sechi A., Tosti A. (2025). Glucagon-like Peptide-1 Receptor Agonist Medications and Hair Loss: A Retrospective Cohort Study. J. Am. Acad. Dermatol..

[8] Rojas Lopez R.F., Lynett Barrera D., Amaya Muñoz M.C., Saavedra Diaz M.P. (2025). Alopecia as an Emerging Adverse Effect Associated With Glucagon-Like Peptide-1 (GLP-1) Receptor Agonists for Weight Loss: A Scoping Review. Cureus.

[9] Lixi F., Calabresi V., Cukurova F., Giannaccare G. (2025). Non-Arteritic Anterior Ischemic Optic Neuropathy in an Otherwise Healthy Young Adult Patient Treated with Liraglutide and Semaglutide for Weight Loss: A Cautionary Tale. Int. Med. Case Rep. J..

[10] Castellana E., Chiappetta M.R. (2026). Exploring the Potential Link Between Tirzepatide and Non-Arteritic Anterior Ischemic Optic Neuropathy (NAION): Evidence from FAERS and Google Trends. Hosp. Pharm..

[11] Winnenburg R., Sorbello A., Bodenreider O. (2015). Exploring Adverse Drug Events at the Class Level. J. Biomed. Semant..

[12] Jose J., Al Rubaie M.H., Al Ramimy H., Varughese S.S. (2021). Pharmacovigilance. Sultan Qaboos Univ. Med. J..

[13] MarketStat Insights Top-Selling Drugs of 2025: Keytruda Still on Top, but Lilly’s Getting Close. https://marketstatics.com/top-selling-drugs-of-2025-keytruda-still-on-top-but-lillys-getting-close/.

[14] Murray-Thomas T., Dcruz J.M., Harder-Lauridsen N.M., Olsen A.H., Williams R., Major-Pedersen A. (2025). Real-world Use of Liraglutide for Weight Management According to Label in the United Kingdom: A Cohort Study Using the Clinical Practice Research Datalink Primary Care Databases. Diabetes Obes. Metab..

[15] Milani I., Guarisco G., Chinucci M., Gaita C., Leonetti F., Capoccia D. (2024). Sex-Differences in Response to Treatment with Liraglutide 3.0 Mg. J. Clin. Med..

[16] Cooper A.J., Gupta S.R., Moustafa A.F., Chao A.M. (2021). Sex/Gender Differences in Obesity Prevalence, Comorbidities, and Treatment. Curr. Obes. Rep..

[17] Muscogiuri G., Verde L., Vetrani C., Barrea L., Savastano S., Colao A. (2023). Obesity: A Gender-View. J. Endocrinol. Investig..

[18] Sbraccia P., Busetto L., Santini F., Mancuso M., Nicoziani P., Nicolucci A. (2021). Misperceptions and Barriers to Obesity Management: Italian Data from the ACTION-IO Study. Eat. Weight. Disord.-Stud. Anorex. Bulim. Obes..

[19] Wang Y., Beydoun M.A., Min J., Xue H., Kaminsky L.A., Cheskin L.J. (2020). Has the Prevalence of Overweight, Obesity and Central Obesity Levelled off in the United States? Trends, Patterns, Disparities, and Future Projections for the Obesity Epidemic. Int. J. Epidemiol..

[20] Kan H., Swindle J.P., Bae J., Dunn J.P., Buysman E.K., Gronroos N.N., Bengtson L., Chinthammit C., Ford J., Ahmad N. (2023). Weight Management Treatment Modalities in Patients with Overweight or Obesity: A Retrospective Cohort Study of Administrative Claims Data. Obes. Pillars.

[21] Tchang B.G., Mihai A.C., Stefanski A., García-Pérez L., Mojdami D., Jouravskaya I., Gurbuz S., Taylor R., Karanikas C.A., Dunn J.P. (2025). Body Weight Reduction in Women Treated with Tirzepatide by Reproductive Stage: A Post Hoc Analysis from the SURMOUNT Program. Obesity.

[22] Zopf Y., Rabe C., Neubert A., Gaßmann K.G., Rascher W., Hahn E.G., Brune K., Dormann H. (2008). Women Encounter ADRs More Often than Do Men. Eur. J. Clin. Pharmacol..

[23] Watson S., Caster O., Rochon P.A., den Ruijter H. (2019). Reported Adverse Drug Reactions in Women and Men: Aggregated Evidence from Globally Collected Individual Case Reports during Half a Century. eClinicalMedicine.

[24] Dahl D., Onishi Y., Norwood P., Huh R., Bray R., Patel H., Rodríguez Á. (2022). Effect of Subcutaneous Tirzepatide vs Placebo Added to Titrated Insulin Glargine on Glycemic Control in Patients With Type 2 Diabetes. JAMA.

[25] Del Prato S., Kahn S.E., Pavo I., Weerakkody G.J., Yang Z., Doupis J., Aizenberg D., Wynne A.G., Riesmeyer J.S., Heine R.J. (2021). Tirzepatide versus Insulin Glargine in Type 2 Diabetes and Increased Cardiovascular Risk (SURPASS-4): A Randomised, Open-Label, Parallel-Group, Multicentre, Phase 3 Trial. Lancet.

[26] Frías J.P., Davies M.J., Rosenstock J., Pérez Manghi F.C., Fernández Landó L., Bergman B.K., Liu B., Cui X., Brown K. (2021). Tirzepatide versus Semaglutide Once Weekly in Patients with Type 2 Diabetes. N. Engl. J. Med..

[27] Garvey W.T., Frias J.P., Jastreboff A.M., le Roux C.W., Sattar N., Aizenberg D., Mao H., Zhang S., Ahmad N.N., Bunck M.C. (2023). Tirzepatide Once Weekly for the Treatment of Obesity in People with Type 2 Diabetes (SURMOUNT-2): A Double-Blind, Randomised, Multicentre, Placebo-Controlled, Phase 3 Trial. Lancet.

[28] Jastreboff A.M., Aronne L.J., Ahmad N.N., Wharton S., Connery L., Alves B., Kiyosue A., Zhang S., Liu B., Bunck M.C. (2022). Tirzepatide Once Weekly for the Treatment of Obesity. N. Engl. J. Med..

[29] Khurana A., Rabbani S.A., El-Tanani M., Arora M.K., Sharma S., Dubey H., Aljabali A.A., Tambuwala M.M. (2024). Safety Profile of Tirzepatide: A Real-World Pharmacovigilance Analysis of EudraVigilance Database. Clin. Epidemiol. Glob. Health.

[30] Kushner P., Anderson J.E., Simon J., Boye K.S., Ranta K., Torcello-Gómez A., Levine J.A. (2023). Efficacy and Safety of Tirzepatide in Adults With Type 2 Diabetes: A Perspective for Primary Care Providers. Clin. Diabetes.

[31] Liu L., Chen J., Wang L., Chen C., Chen L. (2022). Association between Different GLP-1 Receptor Agonists and Gastrointestinal Adverse Reactions: A Real-World Disproportionality Study Based on FDA Adverse Event Reporting System Database. Front. Endocrinol..

[32] Liu L. (2024). A Real-World Data Analysis of Tirzepatide in the FDA Adverse Event Reporting System (FAERS) Database. Front. Pharmacol..

[33] Ludvik B., Giorgino F., Jódar E., Frias J.P., Fernández Landó L., Brown K., Bray R., Rodríguez Á. (2021). Once-Weekly Tirzepatide versus Once-Daily Insulin Degludec as Add-on to Metformin with or without SGLT2 Inhibitors in Patients with Type 2 Diabetes (SURPASS-3): A Randomised, Open-Label, Parallel-Group, Phase 3 Trial. Lancet.

[34] Rosenstock J., Wysham C., Frías J.P., Kaneko S., Lee C.J., Fernández Landó L., Mao H., Cui X., Karanikas C.A., Thieu V.T. (2021). Efficacy and Safety of a Novel Dual GIP and GLP-1 Receptor Agonist Tirzepatide in Patients with Type 2 Diabetes (SURPASS-1): A Double-Blind, Randomised, Phase 3 Trial. Lancet.

[35] Seo Y.-G. (2021). Side Effects Associated with Liraglutide Treatment for Obesity as Well as Diabetes. J. Obes. Metab. Syndr..

[36] Wadden T.A., Chao A.M., Machineni S., Kushner R., Ard J., Srivastava G., Halpern B., Zhang S., Chen J., Bunck M.C. (2023). Tirzepatide after Intensive Lifestyle Intervention in Adults with Overweight or Obesity: The SURMOUNT-3 Phase 3 Trial. Nat. Med..

[37] Wilding J.P.H., Batterham R.L., Calanna S., Davies M., Van Gaal L.F., Lingvay I., McGowan B.M., Rosenstock J., Tran M.T.D., Wadden T.A. (2021). Once-Weekly Semaglutide in Adults with Overweight or Obesity. N. Engl. J. Med..

[38] European Medicines Agency (2015). Saxenda (Liraglutide): EPAR–Product Information.

[39] European Medicines Agency (2022). Wegovy (Semaglutide): EPAR–Product Information.

[40] European Medicines Agency (2022). Mounjaro (Tirzepatide): EPAR–Product Information.

[41] Medicines and Healthcare Products Regulatory Agency (MHRA) (2022). Class 4 Medicines Defect Information: Novo Nordisk Limited, NovoRapid FlexTouch 100 Units/Ml and Saxenda FlexTouch (Liraglutide 6 Mg/Ml)–EL (22)A/33.

[42] Rowley K. November 2025 Drug Safety: Recalls for Compounded Semaglutide, Generic Synthroid; Safety Updates and Microneedling Warnings. https://news.askapatient.com/p/november-2025-drug-safety-recalls-warnings-microneedling.

[43] BioPharma Dive Lilly Warns GLP-1 Knockoffs May Be ‘Dangerous,’ Escalating War with Compounders. https://www.biopharmadive.com/news/lilly-obesity-zepbound-compounder-fda-impurity-/814536/.

[44] ByrdAdatto Alert: FDA Confirms End of Tirzepatide Drug Shortage. https://byrdadatto.com/banter/alert-fda-confirms-end-of-tirzepatide-drug-shortage/.

[45] U.S. Food and Drug Administration (2026). FDA Warns 30 Telehealth Companies Against Illegal Marketing of Compounded GLP-1s.

[46] Medicines and Healthcare Products Regulatory Agency (MHRA) (2026). MHRA Safety Roundup: February 2026.

[47] Rodriguez P.J., Goodwin Cartwright B.M., Gratzl S., Brar R., Baker C., Gluckman T.J., Stucky N.L. (2024). Semaglutide vs Tirzepatide for Weight Loss in Adults with Overweight or Obesity. JAMA Intern. Med..

[48] Shil K.K., Hira A.D., Bakchi S., Paul S.K., Hossain M., Ghosh D.K. (2025). Efficacy and Safety of Tirzepatide and Semaglutide for Obesity Management: A Real-World Comparison. Cureus.

[49] Aronne L.J., Horn D.B., le Roux C.W., Ho W., Falcon B.L., Gomez Valderas E., Das S., Lee C.J., Glass L.C., Senyucel C. (2025). Tirzepatide as Compared with Semaglutide for the Treatment of Obesity. N. Engl. J. Med..

[50] Aronne L.J., Sattar N., Horn D.B., Bays H.E., Wharton S., Lin W.-Y., Ahmad N.N., Zhang S., Liao R., Bunck M.C. (2024). Continued Treatment with Tirzepatide for Maintenance of Weight Reduction in Adults with Obesity. JAMA.

[51] Mathew A., Hannoodee H. (2023). FRI643 Tirzepatide Associated Partial Small Bowel Obstruction: A Case Report. J. Endocr. Soc..

[52] Nahar S., Maybee N., Tamanna N., Sadat A., Khanam F., Begum R., Akther S., Khan M.S., Sonia S.N., Hasan N. (2025). Severe Small-Bowel Obstruction in a High-Risk Patient on Long-Term Tirzepatide Therapy: A Case Report. Cureus.

[53] Iskander M., Wadhwa M., Kim Y., Singh N., Pathak P. (2025). Acute Functional Gastric Outlet Obstruction Associated with Low-Dose Tirzepatide. Cureus.

[54] Pi-Sunyer X., Astrup A., Fujioka K., Greenway F., Halpern A., Krempf M., Lau D.C.W., le Roux C.W., Violante Ortiz R., Jensen C.B. (2015). A Randomized, Controlled Trial of 3.0 Mg of Liraglutide in Weight Management. N. Engl. J. Med..

[55] Javed H., Kogilathota Jagirdhar G.S., Kashyap R., Vekaria P.H. (2023). Liraglutide-Induced Pancreatitis: A Case Report and Literature Review. Cureus.

[56] AlSaadoun A.R., AlSaadoun T.R., Al Ghumlas A.K. (2022). Liraglutide Overdose-Induced Acute Pancreatitis. Cureus.

[57] Guo H., Guo Q., Li Z., Wang Z. (2024). Association between Different GLP-1 Receptor Agonists and Acute Pancreatitis: Case Series and Real-World Pharmacovigilance Analysis. Front. Pharmacol..

[58] Dolan R.D., Bazarbashi A.N., Lo A., Smith B.N. (2020). Liraglutide-Induced Hemorrhagic Pancreatitis in a Nondiabetic Patient. ACG Case Rep. J..

[59] Willemer S., Adler G. (1989). Histochemical and Ultrastructural Characteristics of Tubular Complexes in Human Acute Pancreatitis. Dig. Dis. Sci..

[60] Butler A.E., Campbell-Thompson M., Gurlo T., Dawson D.W., Atkinson M., Butler P.C. (2013). Marked Expansion of Exocrine and Endocrine Pancreas With Incretin Therapy in Humans With Increased Exocrine Pancreas Dysplasia and the Potential for Glucagon-Producing Neuroendocrine Tumors. Diabetes.

[61] Wen J., Nadora D., Bernstein E., How-Volkman C., Truong A., Joy B., Kou M., Muttalib Z., Alam A., Frezza E. (2025). Evaluating the Rates of Pancreatitis and Pancreatic Cancer Among GLP-1 Receptor Agonists: A Systematic Review and Meta-Analysis of Randomised Controlled Trials. Endocrinol. Diabetes Metab..

[62] Jalleh R.J., Marathe C.S., Rayner C.K., Jones K.L., Umapathysivam M.M., Wu T., Quast D.R., Plummer M.P., Nauck M.A., Horowitz M. (2024). Physiology and Pharmacology of Effects of GLP-1-Based Therapies on Gastric, Biliary and Intestinal Motility. Endocrinology.

[63] Aldhaleei W.A., Abegaz T.M., Bhagavathula A.S. (2024). Glucagon-like Peptide-1 Receptor Agonists Associated Gastrointestinal Adverse Events: A Cross-Sectional Analysis of the National Institutes of Health All of Us Cohort. Pharmaceuticals.

[64] Wen J., Puglisi J., Frezza E. (2025). Association Between Semaglutide or Tirzepatide Therapy and Residual Gastric Content: A Potential Danger During Upper Endoscopy. Cureus.

[65] Chaudhry A., Gabriel B., Noor J., Jawad S., Challa S.R. (2024). Tendency of Semaglutide to Induce Gastroparesis: A Case Report. Cureus.

[66] Singhal R., Sachdeva D., Wortman K., Lall R. (2025). Unmasking Semaglutide-Induced Gastroparesis: The Dangers of Rapid Dose Escalation in a Diabetic Patient. Cureus.

[67] Klein S.R., Hobai I.A. (2023). Semaglutide, Delayed Gastric Emptying, and Intraoperative Pulmonary Aspiration: A Case Report. Can. J. Anesth./J. Can. D’anesthésie.

[68] Friedrichsen M., Breitschaft A., Tadayon S., Wizert A., Skovgaard D. (2021). The Effect of Semaglutide 2.4 Mg Once Weekly on Energy Intake, Appetite, Control of Eating, and Gastric Emptying in Adults with Obesity. Diabetes Obes. Metab..

[69] Lundgren J.R., Janus C., Jensen S.B.K., Juhl C.R., Olsen L.M., Christensen R.M., Svane M.S., Bandholm T., Bojsen-Møller K.N., Blond M.B. (2021). Healthy Weight Loss Maintenance with Exercise, Liraglutide, or Both Combined. N. Engl. J. Med..

[70] He L., Wang J., Ping F., Yang N., Huang J., Li Y., Xu L., Li W., Zhang H. (2022). Association of Glucagon-Like Peptide-1 Receptor Agonist Use With Risk of Gallbladder and Biliary Diseases. JAMA Intern. Med..

[71] Wadden T.A., Bailey T.S., Billings L.K., Davies M., Frias J.P., Koroleva A., Lingvay I., O’Neil P.M., Rubino D.M., Skovgaard D. (2021). Effect of Subcutaneous Semaglutide vs Placebo as an Adjunct to Intensive Behavioral Therapy on Body Weight in Adults With Overweight or Obesity. JAMA.

[72] Ramírez-Mejía M.M., Ponciano-Rodriguez G., Eslam M., Méndez-Sánchez N. (2025). GLP-1 Receptor Agonists and Gallbladder Disease Risk: Insights into Molecular Mechanisms and Clinical Implications. Ther. Adv. Endocrinol. Metab..

[73] Safwan M., Bourgleh M.S., Alotaibi S.A., Alotaibi E., Al-Ruqi A., El Raeya F. (2025). Gastrointestinal Safety of Semaglutide and Tirzepatide vs. Placebo in Obese Individuals without Diabetes: A Systematic Review and Meta Analysis. Ann. Saudi Med..

[74] Nreu B., Dicembrini I., Tinti F., Mannucci E., Monami M. (2020). Cholelithiasis in Patients Treated with Glucagon-Like Peptide-1 Receptor: An Updated Meta-Analysis of Randomized Controlled Trials. Diabetes Res. Clin. Pract..

[75] Hebsgaard J.B., Pyke C., Yildirim E., Knudsen L.B., Heegaard S., Kvist P.H. (2018). Glucagon-like Peptide-1 Receptor Expression in the Human Eye. Diabetes Obes. Metab..

[76] Wang L., Volkow N.D., Kaelber D.C., Xu R. (2025). Semaglutide or Tirzepatide and Optic Nerve and Visual Pathway Disorders in Type 2 Diabetes. JAMA Netw. Open.

[77] Gregory V., Mollan S.P. (2025). Are Synthetic Incretins Associated with Ischaemic Optic Neuropathy?. Eye.

[78] Burke O.M., Sa B., Cespedes D.A., Tosti A. (2025). Dermatologic Implications of Glucagon-Like Peptide-1 Receptor Agonist Medications. Ski. Appendage Disord..

[79] Ahern S. (2025). Allodynia and Dysesthesia Associated with Semaglutide and Tirzepatide. Cureus.

[80] Wharton S., Freitas P., Hjelmesæth J., Kabisch M., Kandler K., Lingvay I., Quiroga M., Rosenstock J., Garvey W.T. (2025). Once-Weekly Semaglutide 7·2 Mg in Adults with Obesity (STEP UP): A Randomised, Controlled, Phase 3b Trial. Lancet Diabetes Endocrinol..

[81] Moreno-Borque R., Guhl-Millán G., Mera-Carreiro S., Pazos-Guerra M., Cortés-Toro J.A., López-Bran E. (2024). Delayed Type Hypersensitivity Reaction Induced By Liraglutide With Tolerance to Semaglutide. JCEM Case Rep..

[82] Ayhan I., Topaloğlu Ö., Tekin S., Bayraktaroğlu T. (2023). Injection Site Reaction Induced by Liraglutide Use in a Female Patient with Obesity. JCEM Case Rep..

[83] Kamila J., Marcelina P.-G., Ewa O.-B., Adam R. (2019). Injection Site Reaction during Liraglutide Therapy. J. Dermatol. Plast. Surg..

[84] Buontempo M.G., Santos B.T. (2025). Exploring the Hair Loss Risk in Glucagon-like Peptide-1 Agonists: Emerging Concerns and Clinical Implications. J. Eur. Acad. Dermatol. Venereol..

[85] Godfrey H., Leibovit-Reiben Z., Jedlowski P., Thiede R. (2025). Alopecia Associated with the Use of Semaglutide and Tirzepatide: A Disproportionality Analysis Using the FDA Adverse Event Reporting System (FAERS) from 2022 to 2023. J. Eur. Acad. Dermatol. Venereol..

[86] World Health Organization WHO Medical Product Alert–Semaglutide Medicines and NAION. https://www.who.int/news/item/27-06-2025-27-06-2025-semaglutide-medicines-naion.

[87] Branyiczky M.K., Lowe M.S., McMullen E., Donovan J., Khosravi-Hafshejani T. (2026). Effects of GLP-1 Receptor Agonists on Hair Loss and Regrowth: A Systematic Review. Int. J. Dermatol..

[88] Medicines and Healthcare Products Regulatory Agency MHRA Updates Guidance for GLP-1 Prescribers and Patients. https://www.gov.uk/government/news/mhra-updates-guidance-for-glp-1-prescribers-and-patients?utm_source=chatgpt.com.

[89] Popa Ilie I.R., Dobrea C.M., Butuca A., Homorodean C., Morgovan C., Vonica-Tincu A.L., Gligor F.G., Ghibu S., Frum A. (2024). Real-Life Data on the Safety of Pasireotide in Acromegaly: Insights from EudraVigilance. Pharmaceuticals.

[90] Noguchi Y., Yoshizawa S., Aoyama K., Kubo S., Tachi T., Teramachi H. (2021). Verification of the “Upward Variation in the Reporting Odds Ratio Scores” to Detect the Signals of Drug–Drug Interactions. Pharmaceutics.

[91] Pono P., Cheng V., Skerrett V., Jones A.M. (2025). Post-Marketing Pharmacovigilance Study of Darunavir in the United Kingdom: An Analysis of Adverse Drug Reactions Reported to the MHRA. Pharmacoepidemiology.

[92] Calapai F., Mannucci C., McQuain L., Salvo F. (2023). Pharmacological Evaluation of Signals of Disproportionality Reporting Related to Adverse Reactions to Antiepileptic Cannabidiol in VigiBase. Pharmaceuticals.

[93] Cutroneo P.M., Sartori D., Tuccori M., Crisafulli S., Battini V., Carnovale C., Rafaniello C., Capuano A., Poluzzi E., Moretti U. (2024). Conducting and Interpreting Disproportionality Analyses Derived from Spontaneous Reporting Systems. Front. Drug Saf. Regul..

[94] Caster O., Juhlin K., Watson S., Norén G.N. (2014). Improved Statistical Signal Detection in Pharmacovigilance by Combining Multiple Strength-of-Evidence Aspects in VigiRank. Drug Saf..

[95] World Health Organization–Uppsala Monitoring Centre (WHO-UMC) (2016). Measures of Disproportionate Reporting.

[96] Popa Ilie I.R., Vonica-Tincu A.L., Dobrea C.M., Butuca A., Frum A., Morgovan C., Gligor F.G., Ghibu S. (2024). Safety Profiles Related to Dosing Errors of Rapid-Acting Insulin Analogs: A Comparative Analysis Using the EudraVigilance Database. Biomedicines.

[97] Lin B., Chu F., Ren Y., Jia B., Yuan N., Shang H., Feng T., Zhu Y., Ding J. (2014). Alkaline stable C2-substituted imidazolium-based cross-linked anion exchange membranes for alkaline fuel cell applications. J. Power Sources.

[98] López-Saucedo F., Zúñiga-Villarreal N., Flores-Rojas G.G., Martínez-Otero D., Magariños B., Bucio E. (2020). Zinc heterocyclic vinyl complexes and their gamma-irradiated derivatives: From the metal to antimicrobial materials. React. Funct. Polym..

[99] Sterkhova I.V., Parshina L.N., Grishchenko L.A., Borodina T.y.N., Belovezhets L.A., Semenov V.A. (2023). Complexes of zinc(II) chloride and acetate with propargylimidazoles: Synthesis, structure and non-covalent interactions. Struct. Chem..

[100] Krause L., Herbst-Irmer R., Sheldrick G.M., Stalke D. (2015). Comparison of silver and molybdenum microfocus X-ray sources for single-crystal structure determination. J. Appl. Crystallogr..

[101] Sheldrick G. (2015). SHELXT—Integrated space-group and crystal-structure determination. Acta Crystallogr. Sect. A.

[102] Sheldrick G. (2015). Crystal structure refinement with SHELXL. Acta Crystallogr. Sect. C.

