# Supplementary material for: Incretin-Based Drugs for Obesity: Common and Drug-Specific Reporting Patterns of Adverse Drug Reactions—A Comparative Disproportionality Analysis Using EudraVigilance Reports Integrating SmPC Data
Source: Pharmaceuticals (Basel). 2026 May 31;19(6):876. doi: 10.3390/ph19060876 (PMC13305957; doi:10.3390/ph19060876)
Supplement: Supplementary file 1 [file pharmaceuticals-19-00876-s001.zip › pharmaceuticals-4318004-supplementary.pdf]

Supplementary Materials

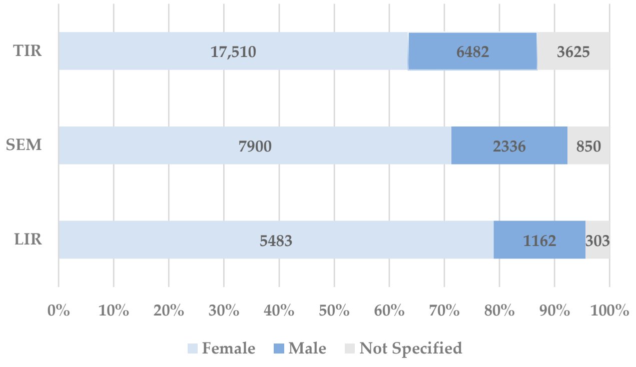

Supplementary Figure S1. Distribution of case by sex

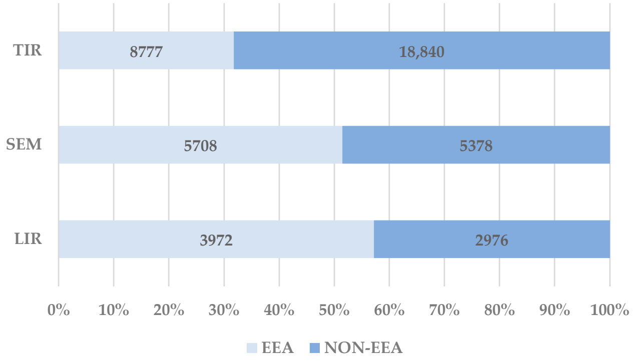

Supplementary Figure S2. Distribution of cases by region

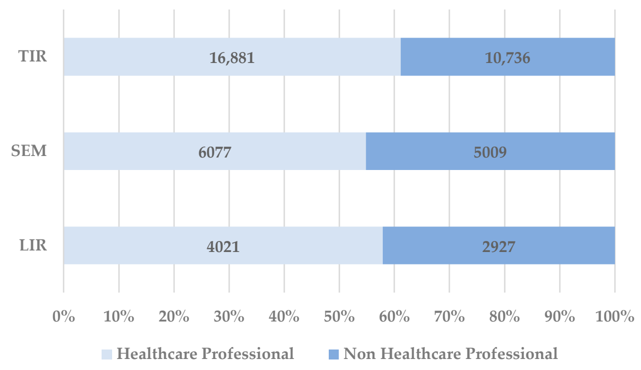

Supplementary Figure S3. Distribution of cases by reporter category

Supplementary Table S1. Contingency table for serious – non-serious cases corrected with Haldane-Anscombe ½ correction

	Serious case	Non-serious Case	Total
Drug of interest	a + 0.5	b + 0.5	n1
Comparator drug	c + 0.5	d + 0.5	n2
Total	m1	m2	N

a - number of serious cases for the drug of interest
b - number of non-serious cases for the drug of interest
c - number of serious cases for the comparator.
d - number of non-serious cases for the comparator.

Supplementary Table S2. Differences and similarities of the adverse drug reactions listed in the Summary of Product Characteristics for the three drugs

System organ class	Common ADRs	ADRs listed for tirzepatide	ADRs listed for semaglutide	ADRs listed for liraglutide	ADRs listed for tirzepatide and liraglutide	ADRs listed for tirzepatide and semaglutide	ADRs listed for liraglutide and semaglutide

Immune system disorder	Anaphylactic reaction	Hypersensitivity reactions				Angioedema	
Metabolism and nutrition disorders	Hypoglycaemia / Hypoglycaemia* when used with sulphonylurea, insulin, metformin and/or SGLT2i, *	Decreased appetite***, Weight decreased***		Dehydration			
Psychiatric disorders				Insomnia			
Nervous system disorders	Dizziness, Dysgeusia					Dysaesthesia	Headache
Gastrointestinal disorders	Nausea, Diarrhoea, Vomiting, Constipation, Dyspepsia, Abdominal distention, Flatulence, Gastroesophageal reflux disease, Delayed gastric emptying, Acute pancreatitis / Pancreatitis, Abdominal pain upper / Abdominal pain			Dry mouth			Gastritis, Intestinal obstruction
Skin and subcutaneous tissue disorders				Rash, Urticaria, Cutaneous amyloidosis		Hair loss	
General disorders and administration site conditions	Fatigue, Injection site reactions, Injection site pain			Asthenia, Malaise			
Investigations	Lipase increased, Amylase increased	Blood calcitonin increased					
Cardiac disorders	Heart rate increased / Increased heart rate / Tachycardia		Orthostatic hypotension			Hypotension	
Hepatobiliary disorders	Cholelithiasis				Cholecystitis		
Renal and urinary disorders				Acute renal failure, Renal impairment			

Eye disorders	*	Diabetic retinopathy in patients with type 2 diabetes**, Non-arteritic anterior ischaemic optic neuropathy (NAION)

* specific ADRs to antidiabetic drugs co-administered with GLP-1 RAs; ** complications of diabetes mellitus; *** effect of GLP-1 RAs

Supplementary Table S3. Preferred terms used for labelling the ADRs reported in Summaries of Product Characteristics (n=73).

SOC	PT	TIR	SEM	LIR
Cardiac disorders	Postural orthostatic tachycardia syndrome	8 (0.03%), rank 61	5 (0.05%), rank 59	1 (0.01%), rank 59
	Sinus tachycardia	6 (0.02%), rank 63	4 (0.04%), rank 61	5 (0.07%), rank 61
	Tachyarrhythmia	2 (0.01%), rank 68	0 (0.00%), rank 65	1 (0.01%), rank 65
	Tachycardia	182 (0.66%), rank 36	56 (0.51%), rank 36	52 (0.75%), rank 36
Eye disorders	Diabetic retinopathy	33 (0.12%), rank 54	4 (0.04%), rank 61	2 (0.03%), rank 61
	Optic ischemic neuropathy	78 (0.28%), rank 49	75 (0.68%), rank 29	12 (0.17%), rank 29
Gastrointestinal disorders	Abdominal discomfort	327 (1.18%), rank 27	115 (1.04%), rank 22	72 (1.04%), rank 22
	Abdominal distension	456 (1.65%), rank 21	162 (1.46%), rank 19	126 (1.81%), rank 19
	Abdominal pain	1711 (6.20%), rank 5	585 (5.28%), rank 5	286 (4.12%), rank 5
	Abdominal pain lower	38 (0.14%), rank 53	14 (0.13%), rank 50	8 (0.12%), rank 50
	Abdominal pain upper	1101 (3.99%), rank 8	405 (3.65%), rank 8	221 (3.18%), rank 8
	Constipation	1456 (5.27%), rank 6	612 (5.52%), rank 4	302 (4.35%), rank 4
	Diarrhoea	3194 (11.57%), rank 3	1017 (9.17%), rank 3	501 (7.21%), rank 3
	Dry mouth	115 (0.42%), rank 44	56 (0.51%), rank 36	59 (0.85%), rank 36
	Dyspepsia	621 (2.25%), rank 15	243 (2.19%), rank 14	170 (2.45%), rank 14
	Eructation	574 (2.08%), rank 17	225 (2.03%), rank 15	129 (1.86%), rank 15
	Flatulence	277 (1.00%), rank 29	110 (0.99%), rank 23	83 (1.19%), rank 23
	Gastritis	159 (0.58%), rank 41	38 (0.34%), rank 42	52 (0.75%), rank 42

	Gastrooesophageal reflux disease	475 (1.72%), rank 18	206 (1.86%), rank 18	80 (1.15%), rank 18
	Impaired gastric emptying	1102 (3.99%), rank 7	321 (2.90%), rank 11	78 (1.12%), rank 11
	Intestinal obstruction	429 (1.55%), rank 22	109 (0.98%), rank 24	33 (0.47%), rank 24
	Nausea	3660 (13.25%), rank 1	1833 (16.53%), rank 1	1046 (15.05%), rank 1
	Pancreatitis	1801 (6.52%), rank 4	382 (3.45%), rank 10	475 (6.84%), rank 10
	Pancreatitis acute	675 (2.44%), rank 14	144 (1.30%), rank 21	125 (1.80%), rank 21
	Vomiting	3315 (12.00%), rank 2	1315 (11.86%), rank 2	611 (8.79%), rank 2
General disorders and administration site conditions	Administration site erythema	3 (0.01%), rank 67	0 (0.00%), rank 65	1 (0.01%), rank 65
	Administration site pruritus	4 (0.01%), rank 65	0 (0.00%), rank 65	0 (0.00%), rank 65
	Administration site reaction	2 (0.01%), rank 68	0 (0.00%), rank 65	0 (0.00%), rank 65
	Application site eythema	5 (0.02%), rank 64	0 (0.00%), rank 65	5 (0.07%), rank 65
	Application site pain	0 (0.00%), rank 71	0 (0.00%), rank 65	2 (0.03%), rank 65
	Application site pruritus	4 (0.01%), rank 65	0 (0.00%), rank 65	5 (0.07%), rank 65
	Application site reaction	2 (0.01%), rank 68	0 (0.00%), rank 65	0 (0.00%), rank 65
	Asthenia	367 (1.33%), rank 25	159 (1.43%), rank 20	101 (1.45%), rank 20
	Fatigue	926 (3.35%), rank 11	569 (5.13%), rank 6	262 (3.77%), rank 6
	Injection site erythema	611 (2.21%), rank 16	32 (0.29%), rank 45	328 (4.72%), rank 45
	Injection site pain	252 (0.91%), rank 31	94 (0.85%), rank 27	92 (1.32%), rank 27
	Injection site pruritus	468 (1.69%), rank 20	12 (0.11%), rank 53	246 (3.54%), rank 53
	Injection site reaction	344 (1.25%), rank 26	5 (0.05%), rank 59	49 (0.71%), rank 59
	Injection site urticaria	83 (0.30%), rank 48	7 (0.06%), rank 58	68 (0.98%), rank 58
	Malaise	397 (1.44%), rank 23	207 (1.87%), rank 17	133 (1.91%), rank 17
Hepatobiliary disorders	Cholecystitis	319 (1.16%), rank 28	95 (0.86%), rank 26	78 (1.12%), rank 26
	Cholecystitis acute	96 (0.35%), rank 47	33 (0.30%), rank 43	20 (0.29%), rank 43
	Cholelithiasis	1068 (3.87%), rank 10	214 (1.93%), rank 16	294 (4.23%), rank 16
	Gallbladder disorder	132 (0.48%), rank 42	106 (0.96%), rank 25	20 (0.29%), rank 25
Immune system disorders	Anaphylactic reaction	177 (0.64%), rank 38	22 (0.20%), rank 47	21 (0.30%), rank 47
	Anaphylactic shock	64 (0.23%), rank 52	9 (0.08%), rank 56	8 (0.12%), rank 56
	Hypersensitivity	234 (0.85%), rank 33	64 (0.58%), rank 32	51 (0.73%), rank 32

Investigations	Amylase increased	129 (0.47%), rank 43	33 (0.30%), rank 43	29 (0.42%), rank 43
	Blood calcitonin increased	10 (0.04%), rank 60	1 (0.01%), rank 64	2 (0.03%), rank 64
	Heart rate increased	198 (0.72%), rank 35	65 (0.59%), rank 31	76 (1.09%), rank 31
	Lipase increased	272 (0.98%), rank 30	58 (0.52%), rank 34	59 (0.85%), rank 34
Metabolism and nutrition disorders	Dehydration	1070 (3.87%), rank 9	257 (2.32%), rank 13	96 (1.38%), rank 13
	Hypoglycaemia	393 (1.42%), rank 24	57 (0.51%), rank 35	61 (0.88%), rank 35
Nervous system disorders	Dizziness	805 (2.91%), rank 12	387 (3.49%), rank 9	234 (3.37%), rank 9
	Dizziness postural	24 (0.09%), rank 58	9 (0.08%), rank 56	2 (0.03%), rank 56
	Dysaesthesia	13 (0.05%), rank 59	39 (0.35%), rank 41	0 (0.00%), rank 41
	Dysgeusia	66 (0.24%), rank 51	47 (0.42%), rank 39	26 (0.37%), rank 39
	Headache	694 (2.51%), rank 13	434 (3.91%), rank 7	305 (4.39%), rank 7
	Lethargy	74 (0.27%), rank 50	14 (0.13%), rank 50	16 (0.23%), rank 50
Psychiatric disorders	Insomnia	177 (0.64%), rank 38	90 (0.81%), rank 28	61 (0.88%), rank 28
Renal and urinary disorders	Renal failure	169 (0.61%), rank 40	18 (0.16%), rank 48	15 (0.22%), rank 48
	Renal impairment	0 (0.00%), rank 71	18 (0.16%), rank 48	16 (0.23%), rank 48
Skin and subcutaneous tissue disorders	Alopecia	471 (1.71%), rank 19	265 (2.39%), rank 12	56 (0.81%), rank 12
	Angioedema	105 (0.38%), rank 46	41 (0.37%), rank 40	13 (0.19%), rank 40
	Cutaneous amyloidosis	0 (0.00%), rank 71	0 (0.00%), rank 65	1 (0.01%), rank 65
	Dermatitis	7 (0.03%), rank 62	3 (0.03%), rank 63	3 (0.04%), rank 63
	Dermatitis allergic	31 (0.11%), rank 56	13 (0.12%), rank 52	16 (0.23%), rank 52
	Eczema	30 (0.11%), rank 57	11 (0.10%), rank 55	5 (0.07%), rank 55
	Erythema	107 (0.39%), rank 45	29 (0.26%), rank 46	42 (0.60%), rank 46
	Rash	230 (0.83%), rank 34	74 (0.67%), rank 30	79 (1.14%), rank 30
	Urticaria	178 (0.64%), rank 37	59 (0.53%), rank 33	72 (1.04%), rank 33
*	Hypotension	249 (0.90%), rank 32	56 (0.51%), rank 36	37 (0.53%), rank 36
	Orthostatic hypotension	33 (0.12%), rank 54	12 (0.11%), rank 53	4 (0.06%), rank 53

Supplementary Table S4. Disproportionality analysis by SOC. IC – information component, LIR – liraglutide, ROR – reporting odds ratio, SEM – semaglutide, TIR – tirzepatide.

SOC	TIR-LIR		TIR-SEM		SEM-LIR		SEM-TIR		LIR-TIR		LIR-SEM	
	ROR (95% CI minim - maxim)	IC (IC025 - IC075)	ROR (95% CI minim - maxim)	IC (IC025 - IC075)	ROR (95% CI minim - maxim)	IC (IC025 - IC075)	ROR (95% CI minim - maxim)	IC (IC025 - IC075)	ROR (95% CI minim - maxim)	IC (IC025 - IC075)	ROR (95% CI minim - maxim)	IC (IC025 - IC075)
Blood and lymphatic system disorders	ROR 2.22 (0.95-5.19)	IC: 0.02 (-0.18 - 0.22)	ROR 1.78 (0.82-3.86)	IC: 0.02 (-0.20 - 0.24)	ROR 1.24 (0.46-3.39)	IC: 0.02 (-0.62 - 0.66)	ROR 1.78 (0.82-3.86)	IC: -0.08 (-0.92 - 0.75)	ROR 2.22 (0.95-5.19)	IC: -0.14 (-1.20 - 0.92)	ROR 1.24 (0.46-3.39)	IC: -0.03 (-0.96 - 0.89)
Cardiac disorders	ROR 3.60 (2.64-4.92)	IC: 0.07 (-0.04 - 0.18)	ROR 1.24 (0.92-1.67)	IC: 0.01 (-0.12 - 0.14)	ROR 2.90 (2.02-4.16)	IC: 0.13 (-0.10 - 0.35)	ROR 1.24 (0.92-1.67)	IC: -0.03 (-0.33 - 0.26)	ROR 3.60 (2.64-4.92)	IC: -0.38 (-0.89 - 0.12)	ROR 2.90 (2.02-4.16)	IC: -0.28 (-0.75 - 0.20)
Congenital, familial and genetic disorders	ROR 0.26 (0.01-5.32)	IC: -0.04 (-0.67 - 0.60)	ROR 0.21 (0.01-4.29)	IC: -0.04 (-0.72 - 0.63)	ROR 1.23 (0.02-64.73)	IC: 0.00 (-0.84 - 0.84)	ROR 0.21 (0.01-4.29)	IC: 0.06 (-0.93 - 1.05)	ROR 0.26 (0.01-5.32)	IC: 0.07 (-1.07 - 1.21)	ROR 1.23 (0.02-64.73)	IC: 0.00 (-1.04 - 1.04)
Ear and labyrinth disorders	ROR 3.56 (1.90-6.67)	IC: 0.12 (-0.21 - 0.45)	ROR 2.63 (1.59-4.33)	IC: 0.15 (-0.23 - 0.52)	ROR 1.36 (0.70-2.63)	IC: 0.07 (-0.62 - 0.76)	ROR 2.63 (1.59-4.33)	IC: -0.34 (-1.12 - 0.45)	ROR 3.56 (1.90-6.67)	IC: -0.58 (-1.83 - 0.67)	ROR 1.36 (0.70-2.63)	IC: -0.15 (-1.36 - 1.06)
Endocrine disorders	ROR 1.35 (0.68-2.67)	IC: 0.02 (-0.31 - 0.36)	ROR 0.59 (0.29-1.22)	IC: -0.04 (-0.40 - 0.33)	ROR 2.28 (1.00-5.21)	IC: 0.07 (-0.37 - 0.51)	ROR 0.59 (0.29-1.22)	IC: 0.06 (-0.46 - 0.59)	ROR 1.35 (0.68-2.67)	IC: -0.05 (-0.79 - 0.69)	ROR 2.28 (1.00-5.21)	IC: -0.11 (-0.80 - 0.59)
Eye disorders	ROR 3.20 (2.22-4.62)	IC: 0.05 (-0.06 - 0.16)	ROR 1.71 (1.34-2.18)	IC: 0.06 (-0.09 - 0.20)	ROR 1.87 (1.28-2.73)	IC: 0.05 (-0.14 - 0.24)	ROR 1.71 (1.34-2.18)	IC: -0.10 (-0.36 - 0.16)	ROR 3.20 (2.22-4.62)	IC: -0.39 (-1.05 - 0.26)	ROR 1.87 (1.28-2.73)	IC: -0.23 (-0.87 - 0.41)
Gastrointestinal disorders	ROR 2.92 (2.69-3.17)	IC: 0.08 (0.04 - 0.12)	ROR 2.40 (2.23-2.57)	IC: 0.10 (0.05 - 0.14)	ROR 1.22 (1.11-1.33)	IC: 0.05 (-0.05 - 0.14)	ROR 2.40 (2.23-2.57)	IC: -0.29 (-0.40 - - 0.18)	ROR 2.92 (2.69-3.17)	IC: -0.43 (-0.58 - - 0.27)	ROR 1.22 (1.11-1.33)	IC: -0.08 (-0.22 - 0.07)

General disorders and administration site conditions	ROR 3.14 (2.81-3.51)	IC: 0.20 (0.11 - 0.29)	ROR 1.55 (1.41-1.71)	IC: 0.09 (0.00 - 0.18)	ROR 2.03 (1.78-2.30)	IC: 0.27 (0.09 - 0.44)	ROR 1.55 (1.41-1.71)	IC: -0.24 (-0.42 - - 0.05)	ROR 3.14 (2.81-3.51)	IC: -0.79 (-1.06 - - 0.52)	ROR 2.03 (1.78-2.30)	IC: -0.40 (-0.66 - - 0.14)
Hepatobiliary disorders	ROR 3.78 (2.53-5.65)	IC: 0.02 (-0.05 - 0.09)	ROR 2.72 (1.81-4.07)	IC: 0.02 (-0.06 - 0.09)	ROR 1.39 (0.91-2.12)	IC: 0.02 (-0.16 - 0.20)	ROR 2.72 (1.81-4.07)	IC: -0.05 (-0.28 - 0.18)	ROR 3.78 (2.53-5.65)	IC: -0.08 (-0.35 - 0.19)	ROR 1.39 (0.91-2.12)	IC: -0.02 (-0.26 - 0.21)
Immune system disorders	ROR 2.73 (1.71-4.35)	IC: 0.04 (-0.10 - 0.19)	ROR 1.76 (1.12-2.76)	IC: 0.03 (-0.13 - 0.18)	ROR 1.55 (0.87-2.75)	IC: 0.08 (-0.42 - 0.57)	ROR 1.76 (1.12-2.76)	IC: -0.13 (-0.75 - 0.48)	ROR 2.73 (1.71-4.35)	IC: -0.30 (-1.07 - 0.47)	ROR 1.55 (0.87-2.75)	IC: -0.11 (-0.79 - 0.57)
Infections and infestations	ROR 2.04 (1.52-2.75)	IC: 0.03 (-0.07 - 0.13)	ROR 1.31 (0.99-1.73)	IC: 0.01 (-0.09 - 0.12)	ROR 1.57 (1.10-2.22)	IC: 0.05 (-0.18 - 0.29)	ROR 1.31 (0.99-1.73)	IC: -0.05 (-0.35 - 0.25)	ROR 2.04 (1.52-2.75)	IC: -0.17 (-0.57 - 0.23)	ROR 1.57 (1.10-2.22)	IC: -0.08 (-0.44 - 0.28)
Injury, poisoning and procedural complications	ROR 1.41 (1.21-1.65)	IC: 0.03 (-0.05 - 0.11)	ROR 1.53 (1.36-1.72)	IC: 0.06 (-0.03 - 0.15)	ROR 0.92 (0.78-1.09)	IC: -0.01 (-0.16 - 0.13)	ROR 1.53 (1.36-1.72)	IC: -0.15 (-0.32 - 0.03)	ROR 1.41 (1.21-1.65)	IC: -0.14 (-0.40 - 0.13)	ROR 0.92 (0.78-1.09)	IC: 0.03 (-0.22 - 0.28)
Investigations	ROR 2.18 (1.84-2.59)	IC: 0.06 (-0.02 - 0.15)	ROR 1.90 (1.63-2.21)	IC: 0.07 (-0.02 - 0.15)	ROR 1.15 (0.94-1.41)	IC: 0.04 (-0.20 - 0.27)	ROR 1.90 (1.63-2.21)	IC: -0.26 (-0.53 - 0.00)	ROR 2.18 (1.84-2.59)	IC: -0.36 (-0.69 - - 0.02)	ROR 1.15 (0.94-1.41)	IC: -0.05 (-0.36 - 0.26)
Metabolism and nutrition disorders	ROR 6.61 (5.58-7.83)	IC: 0.14 (0.07 - 0.21)	ROR 4.65 (4.03-5.36)	IC: 0.16 (0.08 - 0.24)	ROR 1.42 (1.19-1.70)	IC: 0.09 (-0.11 - 0.30)	ROR 4.65 (4.03-5.36)	IC: -0.52 (-0.75 - - 0.29)	ROR 6.61 (5.58-7.83)	IC: -0.81 (-1.15 - - 0.47)	ROR 1.42 (1.19-1.70)	IC: -0.17 (-0.50 - 0.15)
Musculoskeletal and connective tissue disorders	ROR 1.93 (1.45-2.57)	IC: 0.04 (-0.08 - 0.15)	ROR 1.65 (1.34-2.04)	IC: 0.05 (-0.07 - 0.18)	ROR 1.17 (0.85-1.60)	IC: 0.02 (-0.23 - 0.28)	ROR 1.65 (1.34-2.04)	IC: -0.17 (-0.49 - 0.15)	ROR 1.93 (1.45-2.57)	IC: -0.27 (-0.79 - 0.26)	ROR 1.17 (0.85-1.60)	IC: -0.06 (-0.55 - 0.43)
Neoplasms benign, malignant and unspecified (incl cysts and polyps)	ROR 0.59 (0.10-3.42)	IC: 0.00 (-0.13 - 0.12)	ROR 0.26 (0.01-4.52)	IC: 0.00 (-0.12 - 0.11)	ROR 2.29 (0.09- 56.52)	IC: 0.00 (-0.36 - 0.37)	ROR 0.26 (0.01-4.52)	IC: 0.01 (-0.42 - 0.44)	ROR 0.59 (0.10-3.42)	IC: 0.01 (-0.36 - 0.37)	ROR 2.29 (0.09- 56.52)	IC: 0.00 (-0.28 - 0.27)
Nervous system disorders	ROR 3.28 (2.82-3.81)	IC: 0.11 (0.03 - 0.18)	ROR 2.56 (2.26-2.90)	IC: 0.13 (0.04 - 0.21)	ROR 1.28 (1.09-1.50)	IC: 0.06 (-0.11 - 0.22)	ROR 2.56 (2.26-2.90)	IC: -0.30 (-0.49 - - 0.12)	ROR 3.28 (2.82-3.81)	IC: -0.49 (-0.77 - - 0.21)	ROR 1.28 (1.09-1.50)	IC: -0.11 (-0.38 - 0.16)
Pregnancy, puerperium and perinatal conditions	ROR 2.29 (0.77-6.86)	IC: 0.02 (-0.29 - 0.33)	ROR 0.98 (0.30-3.26)	IC: 0.00 (-0.35 - 0.35)	ROR 2.34 (0.67-8.15)	IC: 0.03 (-0.42 - 0.49)	ROR 0.98 (0.30-3.26)	IC: 0.00 (-0.56 - 0.56)	ROR 2.29 (0.77-6.86)	IC: -0.06 (-0.83 - 0.71)	ROR 2.34 (0.67-8.15)	IC: -0.05 (-0.77 - 0.66)
Product issues	ROR 14.41 (8.11- 25.57)	IC: 0.96 (0.35 - 1.56)	ROR 14.33 (8.44- 24.32)	IC: 1.19 (0.55 - 1.83)	ROR 1.01 (0.64-1.58)	IC: 0.00 (-0.77 - 0.78)	ROR 14.33 (8.44- 24.32)	IC: -0.82 (-1.58 - - 0.05)	ROR 14.41 (8.11- 25.57)	IC: -1.06 (-2.05 - - 0.06)	ROR 1.01 (0.64-1.58)	IC: -0.01 (-1.03 - 1.02)
Psychiatric disorders	ROR 3.51 (2.87-4.30)	IC: 0.08 (0.00 - 0.16)	ROR 2.22 (1.88-2.61)	IC: 0.08 (-0.01 - 0.18)	ROR 1.58 (1.28-1.95)	IC: 0.07 (-0.09 - 0.22)	ROR 2.22 (1.88-2.61)	IC: -0.18 (-0.37 - 0.02)	ROR 3.51 (2.87-4.30)	IC: -0.41 (-0.75 - - 0.07)	ROR 1.58 (1.28-1.95)	IC: -0.16 (-0.49 - 0.16)
Renal and urinary disorders	ROR 3.54 (2.25-5.57)	IC: 0.02 (-0.06 - 0.11)	ROR 2.37 (1.51-3.72)	IC: 0.02 (-0.08 - 0.11)	ROR 1.49 (0.88-2.53)	IC: 0.03 (-0.27 - 0.34)	ROR 2.37 (1.51-3.72)	IC: -0.08 (-0.48 - 0.31)	ROR 3.54 (2.25-5.57)	IC: -0.16 (-0.65 - 0.33)	ROR 1.49 (0.88-2.53)	IC: -0.05 (-0.47 - 0.37)

Reproductive system and breast disorders	ROR 2.37 (1.59-3.54)	IC: 0.07 (-0.14 - 0.29)	ROR 2.41 (1.82-3.19)	IC: 0.16 (-0.08 - 0.40)	ROR 0.98 (0.65-1.50)	IC: 0.00 (-0.43 - 0.43)	ROR 2.41 (1.82-3.19)	IC: -0.36 (-0.85 - 0.12)	ROR 2.37 (1.59-3.54)	IC: -0.44 (-1.26 - 0.39)	ROR 0.98 (0.65-1.50)	IC: 0.01 (-0.79 - 0.81)
Respiratory, thoracic and mediastinal disorders	ROR 3.84 (2.64-5.58)	IC: 0.06 (-0.06 - 0.19)	ROR 2.29 (1.68-3.13)	IC: 0.06 (-0.09 - 0.21)	ROR 1.68 (1.12-2.51)	IC: 0.07 (-0.22 - 0.37)	ROR 2.29 (1.68-3.13)	IC: -0.17 (-0.55 - 0.20)	ROR 3.84 (2.64-5.58)	IC: -0.41 (-1.03 - 0.21)	ROR 1.68 (1.12-2.51)	IC: -0.17 (-0.75 - 0.42)
Skin and subcutaneous tissue disorders	ROR 2.64 (2.14-3.27)	IC: 0.13 (-0.02 - 0.29)	ROR 2.90 (2.46-3.43)	IC: 0.25 (0.09 - 0.41)	ROR 0.91 (0.72-1.15)	IC: -0.03 (-0.38 - 0.32)	ROR 2.90 (2.46-3.43)	IC: -0.66 (-1.03 - - 0.29)	ROR 2.64 (2.14-3.27)	IC: -0.68 (-1.20 - - 0.17)	ROR 0.91 (0.72-1.15)	IC: 0.06 (-0.44 - 0.57)
Social circumstances	ROR 0.49 (0.16-1.54)	IC: -0.16 (-1.48 - 1.17)	ROR 0.34 (0.13-0.89)	IC: -0.28 (-1.74 - 1.18)	ROR 1.45 (0.56-3.72)	IC: 0.02 (-0.35 - 0.39)	ROR 0.34 (0.13-0.89)	IC: 0.05 (-0.28 - 0.39)	ROR 0.49 (0.16-1.54)	IC: 0.10 (-0.73 - 0.92)	ROR 1.45 (0.56-3.72)	IC: -0.06 (-1.10 - 0.98)
Surgical and medical procedures	ROR 0.03 (0.01-0.11)	IC: -0.48 (-1.99 - 1.02)	ROR 0.01 (0.00-0.06)	IC: -0.51 (-2.04 - 1.01)	ROR 2.41 (0.47-12.31)	IC: 0.00 (-0.19 - 0.20)	ROR 0.01 (0.00-0.06)	IC: 0.03 (-0.05 - 0.11)	ROR 0.03 (0.01-0.11)	IC: 0.04 (-0.09 - 0.18)	ROR 2.41 (0.47-12.31)	IC: -0.01 (-0.34 - 0.33)
Vascular disorders	ROR 2.43 (1.62-3.63)	IC: 0.03 (-0.09 - 0.15)	ROR 2.34 (1.70-3.23)	IC: 0.06 (-0.08 - 0.19)	ROR 1.04 (0.67-1.60)	IC: 0.00 (-0.30 - 0.31)	ROR 2.34 (1.70-3.23)	IC: -0.17 (-0.56 - 0.21)	ROR 2.43 (1.62-3.63)	IC: -0.21 (-0.77 - 0.36)	ROR 1.04 (0.67-1.60)	IC: -0.01 (-0.52 - 0.51)

Supplementary Table S5. Odds ratios between HPs and non-HPs reporting of TIR ADRs listed in the GLP-1 RAs SmPCs (n=55)

SOC	PT	ROR (95% CI: minim - maxim)	IC (IC025 - IC075)	Significance
Cardiac disorders	Tachycardia	ROR 1.04 (95% CI: 0.77-1.41)	IC: 0.02 (IC025 -0.59 - IC075 0.63)	No Signal
Eye disorders	Optic ischemic neuropathy	ROR 1.27 (95% CI: 0.79-2.04)	IC: 0.12 (IC025 -0.77 - IC075 1.02)	No Signal
Gastrointestinal disorders	Abdominal discomfort	ROR 0.35 (95% CI: 0.27-0.43)	IC: -0.78 (IC025 -1.38 - IC075 -0.18)	No Signal
	Abdominal distension	ROR 0.35 (95% CI: 0.29-0.42)	IC: -0.77 (IC025 -1.28 - IC075 -0.26)	No Signal
	Abdominal pain	ROR 0.65 (95% CI: 0.59-0.72)	IC: -0.25 (IC025 -0.47 - IC075 -0.03)	No Signal
	Abdominal pain lower	ROR 1.56 (95% CI: 0.77-3.15)	IC: 0.21 (IC025 -1.03 - IC075 1.45)	No Signal
	Abdominal pain upper	ROR 0.40 (95% CI: 0.35-0.45)	IC: -0.64 (IC025 -0.95 - IC075 -0.33)	No Signal
	Constipation	ROR 0.40 (95% CI: 0.36-0.44)	IC: -0.62 (IC025 -0.89 - IC075 -0.36)	No Signal
	Diarrhoea	ROR 0.56 (95% CI: 0.52-0.60)	IC: -0.33 (IC025 -0.49 - IC075 -0.17)	No Signal
	Dry mouth	ROR 0.41 (95% CI: 0.28-0.59)	IC: -0.64 (IC025 -1.60 - IC075 0.33)	No Signal
	Dyspepsia	ROR 0.27 (95% CI: 0.23-0.33)	IC: -0.99 (IC025 -1.46 - IC075 -0.52)	No Signal
	Eructation	ROR 0.40 (95% CI: 0.34-0.48)	IC: -0.64 (IC025 -1.07 - IC075 -0.21)	No Signal
	Flatulence	ROR 0.40 (95% CI: 0.31-0.51)	IC: -0.66 (IC025 -1.29 - IC075 -0.03)	No Signal
	Gastritis	ROR 1.08 (95% CI: 0.78-1.49)	IC: 0.04 (IC025 -0.61 - IC075 0.69)	No Signal
	Gastrooesophageal reflux disease	ROR 0.82 (95% CI: 0.68-0.99)	IC: -0.11 (IC025 -0.51 - IC075 0.28)	No Signal
	Impaired gastric emptying	ROR 0.12 (95% CI: 0.10-0.14)	IC: -1.85 (IC025 -2.33 - IC075 -1.38)	No Signal
	Intestinal obstruction	ROR 0.34 (95% CI: 0.28-0.42)	IC: -0.79 (IC025 -1.32 - IC075 -0.27)	No Signal
	Nausea	ROR 0.58 (95% CI: 0.54-0.62)	IC: -0.30 (IC025 -0.45 - IC075 -0.15)	No Signal
	Pancreatitis	ROR 1.53 (95% CI: 1.38-1.70)	IC: 0.20 (IC025 0.02 - IC075 0.38)	Significant Signal
	Pancreatitis acute	ROR 1.10 (95% CI: 0.94-1.29)	IC: 0.05 (IC025 -0.26 - IC075 0.36)	No Signal
	Vomiting	ROR 0.66 (95% CI: 0.61-0.71)	IC: -0.23 (IC025 -0.38 - IC075 -0.08)	No Signal
General disorders and administration site conditions	Asthenia	ROR 0.50 (95% CI: 0.41-0.62)	IC: -0.46 (IC025 -0.97 - IC075 0.05)	No Signal
	Fatigue	ROR 0.77 (95% CI: 0.68-0.88)	IC: -0.15 (IC025 -0.43 - IC075 0.14)	No Signal
	Injection site erythema	ROR 1.07 (95% CI: 0.91-1.27)	IC: 0.04 (IC025 -0.29 - IC075 0.37)	No Signal
	Injection site pain	ROR 0.38 (95% CI: 0.29-0.49)	IC: -0.71 (IC025 -1.38 - IC075 -0.04)	No Signal
	Injection site pruritus	ROR 0.81 (95% CI: 0.67-0.97)	IC: -0.13 (IC025 -0.53 - IC075 0.27)	No Signal
	Injection site reaction	ROR 2.12 (95% CI: 1.65-2.73)	IC: 0.33 (IC025 -0.07 - IC075 0.73)	No Signal
	Injection site urticaria	ROR 1.25 (95% CI: 0.79-1.98)	IC: 0.12 (IC025 -0.76 - IC075 0.99)	No Signal

	Malaise	ROR 0.61 (95% CI: 0.50-0.75)	IC: -0.31 (IC025 -0.78 - IC075 0.15)	No Signal
Hepatobiliary disorders	Cholecystitis	ROR 2.02 (95% CI: 1.56-2.61)	IC: 0.31 (IC025 -0.10 - IC075 0.73)	No Signal
	Cholecystitis acute	ROR 3.46 (95% CI: 1.99-6.00)	IC: 0.46 (IC025 -0.26 - IC075 1.18)	No Signal
	Cholelithiasis	ROR 1.38 (95% CI: 1.21-1.58)	IC: 0.16 (IC025 -0.08 - IC075 0.40)	No Signal
	Gallbladder disorder	ROR 0.47 (95% CI: 0.33-0.66)	IC: -0.52 (IC025 -1.39 - IC075 0.34)	No Signal
Immune system disorders	Anaphylactic reaction	ROR 1.72 (95% CI: 1.23-2.40)	IC: 0.25 (IC025 -0.32 - IC075 0.83)	No Signal
	Anaphylactic shock	ROR 0.53 (95% CI: 0.32-0.86)	IC: -0.42 (IC025 -1.62 - IC075 0.77)	No Signal
	Hypersensitivity	ROR 0.90 (95% CI: 0.69-1.17)	IC: -0.06 (IC025 -0.61 - IC075 0.49)	No Signal
Investigations	Amylase increased	ROR 4.88 (95% CI: 2.84-8.36)	IC: 0.53 (IC025 -0.08 - IC075 1.14)	No Signal
	Heart rate increased	ROR 0.45 (95% CI: 0.34-0.60)	IC: -0.56 (IC025 -1.27 - IC075 0.16)	No Signal
	Lipase increased	ROR 3.85 (95% CI: 2.74-5.41)	IC: 0.49 (IC025 0.06 - IC075 0.91)	Significant Signal
Metabolism and nutrition disorders	Dehydration	ROR 0.49 (95% CI: 0.43-0.55)	IC: -0.47 (IC025 -0.77 - IC075 -0.17)	No Signal
Nervous system disorders	Dizziness	ROR 0.55 (95% CI: 0.48-0.63)	IC: -0.39 (IC025 -0.72 - IC075 -0.05)	No Signal
	Dizziness postural	ROR 0.21 (95% CI: 0.08-0.54)	IC: -1.22 (IC025 -3.77 - IC075 1.33)	No Signal
	Dysaesthesia	ROR 1.44 (95% CI: 0.44-4.67)	IC: 0.17 (IC025 -1.94 - IC075 2.28)	No Signal
	Dysgeusia	ROR 0.72 (95% CI: 0.44-1.17)	IC: -0.20 (IC025 -1.29 - IC075 0.89)	No Signal
	Headache	ROR 0.75 (95% CI: 0.64-0.87)	IC: -0.18 (IC025 -0.51 - IC075 0.16)	No Signal
	Lethargy	ROR 0.54 (95% CI: 0.34-0.86)	IC: -0.40 (IC025 -1.51 - IC075 0.70)	No Signal
Psychiatric disorders	Insomnia	ROR 0.38 (95% CI: 0.28-0.51)	IC: -0.71 (IC025 -1.50 - IC075 0.09)	No Signal
Renal and urinary disorders	Renal failure	ROR 0.56 (95% CI: 0.41-0.76)	IC: -0.38 (IC025 -1.11 - IC075 0.35)	No Signal
Skin and subcutaneous tissue disorders	Alopecia	ROR 0.72 (95% CI: 0.60-0.86)	IC: -0.20 (IC025 -0.61 - IC075 0.21)	No Signal
	Angioedema	ROR 2.16 (95% CI: 1.37-3.41)	IC: 0.34 (IC025 -0.38 - IC075 1.06)	No Signal
	Erythema	ROR 1.57 (95% CI: 1.03-2.38)	IC: 0.22 (IC025 -0.53 - IC075 0.96)	No Signal
	Rash	ROR 1.25 (95% CI: 0.95-1.64)	IC: 0.12 (IC025 -0.41 - IC075 0.64)	No Signal
	Urticaria	ROR 1.60 (95% CI: 1.15-2.21)	IC: 0.23 (IC025 -0.35 - IC075 0.80)	No Signal
Vascular disorders	Hypotension	ROR 1.07 (95% CI: 0.83-1.39)	IC: 0.04 (IC025 -0.48 - IC075 0.56)	No Signal
	Orthostatic hypotension	ROR 0.98 (95% CI: 0.49-1.98)	IC: -0.01 (IC025 -1.45 - IC075 1.43)	No Signal

Supplementary Table S6. Odds ratios between HPs and non-HPs reporting of SEM ADRs listed in the GLP-1 RAs SmPCs (n=53)

SOC	PT	ROR (95% CI: minim - maxim)	IC (IC025 - IC075)	Significance
Cardiac disorders	Tachycardia	ROR 1.74 (95% CI: 0.99-3.06)	IC: 0.30 (IC025 -0.74 - IC075 1.35)	No Signal
Eye disorders	Optic ischemic neuropathy	ROR 3.32 (95% CI: 1.88-5.85)	IC: 0.54 (IC025 -0.29 - IC075 1.37)	No Signal
Gastrointestinal disorders	Abdominal discomfort	ROR 0.37 (95% CI: 0.25-0.55)	IC: -0.80 (IC025 -1.88 - IC075 0.28)	No Signal
	Abdominal distension	ROR 0.45 (95% CI: 0.33-0.63)	IC: -0.61 (IC025 -1.46 - IC075 0.24)	No Signal
	Abdominal pain	ROR 1.37 (95% CI: 1.15-1.63)	IC: 0.18 (IC025 -0.16 - IC075 0.51)	No Signal
	Abdominal pain lower	ROR 0.62 (95% CI: 0.21-1.78)	IC: -0.33 (IC025 -2.88 - IC075 2.22)	No Signal
	Abdominal pain upper	ROR 0.48 (95% CI: 0.39-0.59)	IC: -0.55 (IC025 -1.08 - IC075 -0.03)	No Signal
	Constipation	ROR 0.55 (95% CI: 0.46-0.65)	IC: -0.43 (IC025 -0.84 - IC075 -0.02)	No Signal
	Diarrhoea	ROR 0.76 (95% CI: 0.66-0.86)	IC: -0.18 (IC025 -0.46 - IC075 0.11)	No Signal
	Dry mouth	ROR 0.57 (95% CI: 0.34-0.98)	IC: -0.41 (IC025 -1.75 - IC075 0.93)	No Signal
	Dyspepsia	ROR 0.37 (95% CI: 0.29-0.49)	IC: -0.79 (IC025 -1.52 - IC075 -0.05)	No Signal
	Eructation	ROR 0.41 (95% CI: 0.31-0.55)	IC: -0.69 (IC025 -1.44 - IC075 0.05)	No Signal
	Flatulence	ROR 0.37 (95% CI: 0.24-0.55)	IC: -0.82 (IC025 -1.92 - IC075 0.29)	No Signal
	Gastritis	ROR 0.74 (95% CI: 0.39-1.40)	IC: -0.21 (IC025 -1.72 - IC075 1.31)	No Signal
	Gastrooesophageal reflux disease	ROR 0.65 (95% CI: 0.49-0.85)	IC: -0.31 (IC025 -0.99 - IC075 0.37)	No Signal
	Impaired gastric emptying	ROR 0.24 (95% CI: 0.19-0.31)	IC: -1.22 (IC025 -1.97 - IC075 -0.48)	No Signal
	Intestinal obstruction	ROR 0.35 (95% CI: 0.24-0.53)	IC: -0.85 (IC025 -1.97 - IC075 0.28)	No Signal
	Nausea	ROR 0.67 (95% CI: 0.61-0.74)	IC: -0.24 (IC025 -0.45 - IC075 -0.02)	No Signal
	Pancreatitis	ROR 2.57 (95% CI: 2.03-3.25)	IC: 0.45 (IC025 0.08 - IC075 0.83)	Significant Signal
	Pancreatitis acute	ROR 3.46 (95% CI: 2.29-5.24)	IC: 0.55 (IC025 -0.05 - IC075 1.15)	No Signal
	Vomiting	ROR 0.79 (95% CI: 0.71-0.89)	IC: -0.14 (IC025 -0.39 - IC075 0.11)	No Signal
General disorders and administration site conditions	Asthenia	ROR 0.60 (95% CI: 0.43-0.82)	IC: -0.38 (IC025 -1.17 - IC075 0.41)	No Signal
	Fatigue	ROR 0.76 (95% CI: 0.64-0.89)	IC: -0.19 (IC025 -0.57 - IC075 0.20)	No Signal
	Injection site erythema	ROR 1.21 (95% CI: 0.60-2.44)	IC: 0.11 (IC025 -1.36 - IC075 1.59)	No Signal

	Injection site pain	ROR 0.69 (95% CI: 0.46-1.04)	IC: -0.26 (IC025 -1.24 - IC075 0.73)	No Signal
	Injection site pruritus	ROR 1.16 (95% CI: 0.37-3.64)	IC: 0.08 (IC025 -2.29 - IC075 2.46)	No Signal
	Malaise	ROR 0.65 (95% CI: 0.50-0.86)	IC: -0.30 (IC025 -0.97 - IC075 0.37)	No Signal
Hepatobiliary disorders	Cholecystitis	ROR 2.76 (95% CI: 1.71-4.45)	IC: 0.48 (IC025 -0.27 - IC075 1.24)	No Signal
	Cholecystitis acute	ROR 3.72 (95% CI: 1.54-9.03)	IC: 0.57 (IC025 -0.67 - IC075 1.81)	No Signal
	Cholelithiasis	ROR 2.75 (95% CI: 2.00-3.79)	IC: 0.48 (IC025 -0.02 - IC075 0.99)	No Signal
	Gallbladder disorder	ROR 1.04 (95% CI: 0.71-1.52)	IC: 0.02 (IC025 -0.82 - IC075 0.86)	No Signal
Immune system disorders	Hypersensitivity	ROR 1.29 (95% CI: 0.78-2.13)	IC: 0.15 (IC025 -0.88 - IC075 1.19)	No Signal
Investigations	Amylase increased	ROR 3.73 (95% CI: 1.54-9.03)	IC: 0.57 (IC025 -0.67 - IC075 1.81)	No Signal
	Heart rate increased	ROR 0.48 (95% CI: 0.29-0.80)	IC: -0.56 (IC025 -1.87 - IC075 0.75)	No Signal
	Lipase increased	ROR 2.87 (95% CI: 1.55-5.33)	IC: 0.50 (IC025 -0.47 - IC075 1.46)	No Signal
Metabolism and nutrition disorders	Dehydration	ROR 0.63 (95% CI: 0.49-0.81)	IC: -0.33 (IC025 -0.94 - IC075 0.28)	No Signal
Nervous system disorders	Dizziness	ROR 0.52 (95% CI: 0.42-0.64)	IC: -0.49 (IC025 -1.01 - IC075 0.04)	No Signal
	Dysaesthesia	ROR 5.64 (95% CI: 2.20-14.43)	IC: 0.66 (IC025 -0.45 - IC075 1.76)	No Signal
	Dysgeusia	ROR 0.31 (95% CI: 0.17-0.60)	IC: -0.96 (IC025 -2.73 - IC075 0.81)	No Signal
	Headache	ROR 0.68 (95% CI: 0.56-0.82)	IC: -0.27 (IC025 -0.73 - IC075 0.19)	No Signal
	Lethargy	ROR 0.62 (95% CI: 0.21-1.78)	IC: -0.33 (IC025 -2.88 - IC075 2.22)	No Signal
Psychiatric disorders	Insomnia	ROR 0.57 (95% CI: 0.38-0.87)	IC: -0.41 (IC025 -1.47 - IC075 0.65)	No Signal
Renal and urinary disorders	Renal failure	ROR 1.65 (95% CI: 0.62-4.41)	IC: 0.27 (IC025 -1.57 - IC075 2.11)	No Signal
	Renal impairment	ROR 2.89 (95% CI: 0.95-8.80)	IC: 0.48 (IC025 -1.22 - IC075 2.19)	No Signal
Skin and subcutaneous tissue disorders	Alopecia	ROR 0.48 (95% CI: 0.37-0.61)	IC: -0.56 (IC025 -1.22 - IC075 0.09)	No Signal
	Angioedema	ROR 2.95 (95% CI: 1.40-6.18)	IC: 0.50 (IC025 -0.64 - IC075 1.64)	No Signal
	Dermatitis allergic	ROR 1.32 (95% CI: 0.43-4.04)	IC: 0.16 (IC025 -2.07 - IC075 2.39)	No Signal

	Eczema	ROR 0.69 (95% CI: 0.21-2.25)	IC: -0.25 (IC025 -3.02 - IC075 2.53)	No Signal
	Erythema	ROR 1.84 (95% CI: 0.84-4.04)	IC: 0.32 (IC025 -1.11 - IC075 1.76)	No Signal
	Rash	ROR 1.21 (95% CI: 0.76-1.93)	IC: 0.12 (IC025 -0.86 - IC075 1.09)	No Signal
	Urticaria	ROR 2.05 (95% CI: 1.16-3.60)	IC: 0.37 (IC025 -0.62 - IC075 1.37)	No Signal
Vascular disorders	Hypotension	ROR 1.19 (95% CI: 0.70-2.02)	IC: 0.10 (IC025 -1.02 - IC075 1.23)	No Signal
	Orthostatic hypotension	ROR 1.65 (95% CI: 0.50-5.49)	IC: 0.27 (IC025 -1.97 - IC075 2.50)	No Signal

Supplementary Table S7. Odds ratios between HPs and non-HPs reporting of LIR ADRs listed in the GLP-1 RAs SmPCs (n=50)

SOC	PT	ROR (95% CI: minim - maxim)	IC (IC025 - IC075)	Significance
Cardiac disorders	Tachycardia	ROR 1.27 (95% CI: 0.72-2.23)	IC: 0.13 (IC025 -0.99 - IC075 1.25)	No Signal
Eye disorders	Optic ischemic neuropathy	ROR 0.73 (95% CI: 0.23-2.26)	IC: -0.20 (IC025 -2.75 - IC075 2.36)	No Signal
Gastrointestinal disorders	Abdominal discomfort	ROR 0.24 (95% CI: 0.14-0.41)	IC: -1.19 (IC025 -2.70 - IC075 0.32)	No Signal
	Abdominal distension	ROR 0.40 (95% CI: 0.28-0.57)	IC: -0.69 (IC025 -1.65 - IC075 0.27)	No Signal
	Abdominal pain	ROR 0.91 (95% CI: 0.72-1.15)	IC: -0.06 (IC025 -0.57 - IC075 0.45)	No Signal
	Abdominal pain lower	ROR 0.73 (95% CI: 0.18-2.91)	IC: -0.19 (IC025 -3.26 - IC075 2.88)	No Signal
	Abdominal pain upper	ROR 0.51 (95% CI: 0.39-0.67)	IC: -0.47 (IC025 -1.15 - IC075 0.20)	No Signal
	Constipation	ROR 0.52 (95% CI: 0.41-0.66)	IC: -0.45 (IC025 -1.02 - IC075 0.12)	No Signal
	Diarrhoea	ROR 0.57 (95% CI: 0.48-0.69)	IC: -0.36 (IC025 -0.78 - IC075 0.07)	No Signal
	Dry mouth	ROR 0.70 (95% CI: 0.42-1.17)	IC: -0.23 (IC025 -1.43 - IC075 0.96)	No Signal
	Dyspepsia	ROR 0.60 (95% CI: 0.44-0.81)	IC: -0.35 (IC025 -1.09 - IC075 0.38)	No Signal
	Eructation	ROR 0.41 (95% CI: 0.29-0.59)	IC: -0.66 (IC025 -1.60 - IC075 0.28)	No Signal
	Flatulence	ROR 0.41 (95% CI: 0.26-0.64)	IC: -0.67 (IC025 -1.85 - IC075 0.51)	No Signal
	Gastritis	ROR 0.42 (95% CI: 0.24-0.73)	IC: -0.65 (IC025 -2.12 - IC075 0.82)	No Signal
	Gastrooesophageal reflux disease	ROR 0.69 (95% CI: 0.44-1.07)	IC: -0.24 (IC025 -1.28 - IC075 0.79)	No Signal
	Impaired gastric emptying	ROR 0.13 (95% CI: 0.07-0.24)	IC: -1.87 (IC025 -3.71 - IC075 -0.03)	No Signal
	Intestinal obstruction	ROR 0.61 (95% CI: 0.30-1.20)	IC: -0.34 (IC025 -1.99 - IC075 1.31)	No Signal
	Nausea	ROR 0.77 (95% CI: 0.68-0.88)	IC: -0.14 (IC025 -0.41 - IC075 0.13)	No Signal
	Pancreatitis	ROR 2.63 (95% CI: 2.11-3.27)	IC: 0.42 (IC025 0.09 - IC075 0.75)	Significant Signal

	Pancreatitis acute	ROR 2.56 (95% CI: 1.68-3.91)	IC: 0.42 (IC025 -0.23 - IC075 1.08)	No Signal
	Vomiting	ROR 0.59 (95% CI: 0.50-0.70)	IC: -0.34 (IC025 -0.72 - IC075 0.05)	No Signal
General disorders and administration site conditions	Asthenia	ROR 0.42 (95% CI: 0.28-0.63)	IC: -0.65 (IC025 -1.71 - IC075 0.41)	No Signal
	Fatigue	ROR 0.48 (95% CI: 0.37-0.62)	IC: -0.51 (IC025 -1.14 - IC075 0.11)	No Signal
	Injection site erythema	ROR 1.70 (95% CI: 1.34-2.16)	IC: 0.27 (IC025 -0.16 - IC075 0.69)	No Signal
	Injection site pain	ROR 0.53 (95% CI: 0.35-0.81)	IC: -0.44 (IC025 -1.48 - IC075 0.59)	No Signal
	Injection site pruritus	ROR 1.25 (95% CI: 0.96-1.63)	IC: 0.12 (IC025 -0.39 - IC075 0.64)	No Signal
	Injection site reaction	ROR 3.27 (95% CI: 1.58-6.74)	IC: 0.49 (IC025 -0.53 - IC075 1.51)	No Signal
	Injection site urticaria	ROR 1.53 (95% CI: 0.92-2.55)	IC: 0.22 (IC025 -0.73 - IC075 1.18)	No Signal
	Malaise	ROR 0.48 (95% CI: 0.34-0.68)	IC: -0.53 (IC025 -1.42 - IC075 0.35)	No Signal
Hepatobiliary disorders	Cholecystitis	ROR 2.64 (95% CI: 1.54-4.54)	IC: 0.43 (IC025 -0.39 - IC075 1.26)	No Signal
	Cholelithiasis	ROR 1.48 (95% CI: 1.16-1.90)	IC: 0.21 (IC025 -0.25 - IC075 0.66)	No Signal
	Gallbladder disorder	ROR 0.89 (95% CI: 0.37-2.16)	IC: -0.07 (IC025 -1.99 - IC075 1.85)	No Signal
Immune system disorders	Anaphylactic reaction	ROR 2.34 (95% CI: 0.86-6.40)	IC: 0.38 (IC025 -1.22 - IC075 1.99)	No Signal
	Hypersensitivity	ROR 1.13 (95% CI: 0.64-1.99)	IC: 0.07 (IC025 -1.09 - IC075 1.23)	No Signal
Investigations	Amylase increased	ROR 1.63 (95% CI: 0.74-3.58)	IC: 0.25 (IC025 -1.19 - IC075 1.68)	No Signal
	Heart rate increased	ROR 0.45 (95% CI: 0.28-0.71)	IC: -0.59 (IC025 -1.79 - IC075 0.61)	No Signal
	Lipase increased	ROR 4.10 (95% CI: 2.01-8.34)	IC: 0.55 (IC025 -0.37 - IC075 1.46)	No Signal
Metabolism and nutrition disorders	Dehydration	ROR 0.83 (95% CI: 0.55-1.24)	IC: -0.12 (IC025 -1.02 - IC075 0.78)	No Signal
Nervous system disorders	Dizziness	ROR 0.51 (95% CI: 0.40-0.67)	IC: -0.46 (IC025 -1.11 - IC075 0.19)	No Signal
	Dysgeusia	ROR 0.39 (95% CI: 0.17-0.87)	IC: -0.71 (IC025 -2.82 - IC075 1.40)	No Signal

	Headache	ROR 0.53 (95% CI: 0.42-0.67)	IC: -0.44 (IC025 -1.00 - IC075 0.13)	No Signal
	Lethargy	ROR 0.94 (95% CI: 0.35-2.53)	IC: -0.04 (IC025 -2.15 - IC075 2.07)	No Signal
Psychiatric disorders	Insomnia	ROR 0.54 (95% CI: 0.32-0.90)	IC: -0.43 (IC025 -1.69 - IC075 0.83)	No Signal
Renal and urinary disorders	Renal failure	ROR 1.46 (95% CI: 0.50-4.28)	IC: 0.20 (IC025 -1.81 - IC075 2.20)	No Signal
	Renal impairment	ROR 1.22 (95% CI: 0.44-3.36)	IC: 0.11 (IC025 -1.90 - IC075 2.12)	No Signal
Skin and subcutaneous tissue disorders	Alopecia	ROR 0.34 (95% CI: 0.20-0.60)	IC: -0.83 (IC025 -2.34 - IC075 0.68)	No Signal
	Erythema	ROR 1.19 (95% CI: 0.64-2.22)	IC: 0.10 (IC025 -1.16 - IC075 1.36)	No Signal
	Rash	ROR 1.13 (95% CI: 0.72-1.79)	IC: 0.07 (IC025 -0.86 - IC075 1.00)	No Signal
	Urticaria	ROR 2.21 (95% CI: 1.29-3.77)	IC: 0.37 (IC025 -0.51 - IC075 1.25)	No Signal
Vascular disorders	Hypotension	ROR 0.69 (95% CI: 0.36-1.32)	IC: -0.24 (IC025 -1.75 - IC075 1.27)	No Signal

Supplementary Table S8. Disproportionality analysis of ADRs listed in the SmPCs of liraglutide, semaglutide, and tirzepatide and reported by healthcare professionals. CI – confidence interval, IC – information component, LIR – liraglutide, PT – preferred term, ROR – reporting odds ratio, SEM – semaglutide, TIR - tirzepatide

PT	LIR-SEM		LIR-TIR		SEM-LIR		SEM-TIR		TIR-LIR		TIR-SEM	
	ROR (95% CI: minim - maxim)	IC (IC025 - IC075)	ROR (95% CI: minim - maxim)	IC (IC025 - IC075)	ROR (95% CI: minim - maxim)	IC (IC025 - IC075)	ROR (95% CI: minim - maxim)	IC (IC025 - IC075)	ROR (95% CI: minim - maxim)	IC (IC025 - IC075)	ROR (95% CI: minim - maxim)	IC (IC025 - IC075)
Sinus tachycardia	NA	NA	ROR 4.20 (95% CI: 1.22-14.52)	IC: 1.18 (IC025 - 1.59 - IC075 3.96)	NA	NA	NA	NA	ROR 0.24 (95% CI: 0.07-0.82)	IC: -0.64 (IC025 - 3.42 - IC075 2.14)	NA	NA
Tachycardia	ROR 1.32 (95% CI: 0.82-2.10)	IC: 0.22 (IC025 - 0.90 - IC075 1.34)	ROR 1.23 (95% CI: 0.83-1.81)	IC: 0.23 (IC025 - 0.90 - IC075 1.35)	ROR 0.76 (95% CI: 0.48-1.21)	IC: -0.17 (IC025 - 1.21 - IC075 0.88)	ROR 0.93 (95% CI: 0.65-1.35)	IC: -0.07 (IC025 - 1.12 - IC075 0.98)	ROR 0.81 (95% CI: 0.55-1.20)	IC: -0.06 (IC025 - 0.67 - IC075 0.55)	ROR 1.07 (95% CI: 0.74-1.55)	IC: 0.03 (IC025 - 0.58 - IC075 0.63)
Optic ischemic neuropathy	ROR 0.15 (95% CI: 0.06-0.35)	IC: -2.04 (IC025 - 4.60 - IC075 0.51)	ROR 0.48 (95% CI: 0.21-1.13)	IC: -0.84 (IC025 - 3.40 - IC075 1.71)	ROR 6.67 (95% CI: 2.88-15.45)	IC: 0.59 (IC025 - 0.25 - IC075 1.42)	ROR 3.23 (95% CI: 2.22-4.68)	IC: 1.00 (IC025 0.17 - IC075 1.84)	ROR 2.07 (95% CI: 0.89-4.82)	IC: 0.15 (IC025 - 0.75 - IC075 1.05)	ROR 0.31 (95% CI: 0.21-0.45)	IC: -0.66 (IC025 - 1.56 - IC075 0.24)
Abdominal discomfort	ROR 0.75 (95% CI: 0.43-1.33)	IC: -0.25 (IC025 - 1.76 - IC075 1.26)	ROR 0.65 (95% CI: 0.40-1.07)	IC: -0.51 (IC025 - 2.02 - IC075 1.01)	ROR 1.32 (95% CI: 0.75-2.34)	IC: 0.15 (IC025 - 0.93 - IC075 1.22)	ROR 0.86 (95% CI: 0.59-1.25)	IC: -0.16 (IC025 - 1.24 - IC075 0.92)	ROR 1.54 (95% CI: 0.94-2.53)	IC: 0.10 (IC025 - 0.50 - IC075 0.70)	ROR 1.16 (95% CI: 0.80-1.69)	IC: 0.05 (IC025 - 0.55 - IC075 0.66)
Abdominal distension	ROR 1.18 (95% CI: 0.79-1.74)	IC: 0.13 (IC025 - 0.83 - IC075 1.10)	ROR 1.16 (95% CI: 0.83-1.62)	IC: 0.17 (IC025 - 0.80 - IC075 1.13)	ROR 0.85 (95% CI: 0.58-1.26)	IC: -0.10 (IC025 - 0.94 - IC075 0.75)	ROR 0.99 (95% CI: 0.73-1.33)	IC: -0.01 (IC025 - 0.86 - IC075 0.84)	ROR 0.86 (95% CI: 0.62-1.20)	IC: -0.04 (IC025 - 0.55 - IC075 0.46)	ROR 1.01 (95% CI: 0.75-1.37)	IC: 0.00 (IC025 - 0.50 - IC075 0.51)
Abdominal pain	ROR 0.65 (95% CI: 0.54-0.78)	IC: -0.38 (IC025 - 0.90 - IC075 0.13)	ROR 0.75 (95% CI: 0.63-0.89)	IC: -0.33 (IC025 - 0.84 - IC075 0.19)	ROR 1.54 (95% CI: 1.27-1.87)	IC: 0.21 (IC025 - 0.13 - IC075 0.54)	ROR 1.16 (95% CI: 1.02-1.31)	IC: 0.14 (IC025 - 0.20 - IC075 0.48)	ROR 1.33 (95% CI: 1.12-1.59)	IC: 0.07 (IC025 - 0.15 - IC075 0.28)	ROR 0.87 (95% CI: 0.76-0.98)	IC: -0.05 (IC025 - 0.27 - IC075 0.16)
Abdominal pain lower	ROR 1.01 (95% CI: 0.28-3.58)	IC: 0.01 (IC025 - 3.06 - IC075 3.08)	ROR 0.62 (95% CI: 0.22-1.78)	IC: -0.52 (IC025 - 3.59 - IC075 2.55)	ROR 0.99 (95% CI: 0.28-3.52)	IC: 0.00 (IC025 - 2.56 - IC075 2.55)	ROR 0.62 (95% CI: 0.25-1.49)	IC: -0.51 (IC025 - 3.06 - IC075 2.05)	ROR 1.61 (95% CI: 0.56-4.60)	IC: 0.11 (IC025 - 1.13 - IC075 1.35)	ROR 1.62 (95% CI: 0.67-3.93)	IC: 0.15 (IC025 - 1.09 - IC075 1.39)

Abdominal pain upper	ROR 0.92 (95% CI: 0.71-1.20)	IC: -0.07 (IC025 - 0.75 - IC075 0.60)	ROR 0.89 (95% CI: 0.71-1.12)	IC: -0.13 (IC025 - 0.81 - IC075 0.54)	ROR 1.09 (95% CI: 0.84-1.41)	IC: 0.05 (IC025 - 0.48 - IC075 0.57)	ROR 0.97 (95% CI: 0.80-1.17)	IC: -0.03 (IC025 - 0.56 - IC075 0.49)	ROR 1.12 (95% CI: 0.89-1.41)	IC: 0.03 (IC025 - 0.28 - IC075 0.34)	ROR 1.03 (95% CI: 0.86-1.24)	IC: 0.01 (IC025 - 0.30 - IC075 0.32)
Constipation	ROR 0.77 (95% CI: 0.62-0.96)	IC: -0.23 (IC025 - 0.80 - IC075 0.34)	ROR 0.93 (95% CI: 0.76-1.13)	IC: -0.08 (IC025 - 0.66 - IC075 0.49)	ROR 1.30 (95% CI: 1.04-1.61)	IC: 0.13 (IC025 - 0.27 - IC075 0.54)	ROR 1.21 (95% CI: 1.04-1.40)	IC: 0.19 (IC025 - 0.22 - IC075 0.60)	ROR 1.08 (95% CI: 0.89-1.31)	IC: 0.02 (IC025 - 0.25 - IC075 0.29)	ROR 0.83 (95% CI: 0.71-0.97)	IC: -0.07 (IC025 - 0.34 - IC075 0.19)
Diarrhoea	ROR 0.68 (95% CI: 0.57-0.80)	IC: -0.34 (IC025 - 0.77 - IC075 0.09)	ROR 0.59 (95% CI: 0.51-0.68)	IC: -0.60 (IC025 - 1.03 - IC075 - 0.16)	ROR 1.48 (95% CI: 1.26-1.74)	IC: 0.19 (IC025 - 0.10 - IC075 0.47)	ROR 0.87 (95% CI: 0.78-0.97)	IC: -0.13 (IC025 - 0.42 - IC075 0.16)	ROR 1.70 (95% CI: 1.47-1.96)	IC: 0.11 (IC025 - 0.05 - IC075 0.27)	ROR 1.15 (95% CI: 1.03-1.28)	IC: 0.05 (IC025 - 0.11 - IC075 0.21)
Dry mouth	ROR 1.92 (95% CI: 1.11-3.32)	IC: 0.48 (IC025 - 0.72 - IC075 1.67)	ROR 2.72 (95% CI: 1.70-4.34)	IC: 1.00 (IC025 - 0.20 - IC075 2.20)	ROR 0.52 (95% CI: 0.30-0.90)	IC: -0.44 (IC025 - 1.78 - IC075 0.90)	ROR 1.42 (95% CI: 0.86-2.35)	IC: 0.34 (IC025 - 1.00 - IC075 1.69)	ROR 0.37 (95% CI: 0.23-0.59)	IC: -0.41 (IC025 - 1.37 - IC075 0.56)	ROR 0.70 (95% CI: 0.43-1.17)	IC: -0.15 (IC025 - 1.11 - IC075 0.81)
Dyspepsia	ROR 1.52 (95% CI: 1.11-2.10)	IC: 0.33 (IC025 - 0.41 - IC075 1.06)	ROR 1.71 (95% CI: 1.31-2.23)	IC: 0.57 (IC025 - 0.16 - IC075 1.31)	ROR 0.66 (95% CI: 0.48-0.90)	IC: -0.27 (IC025 - 1.00 - IC075 0.47)	ROR 1.12 (95% CI: 0.86-1.46)	IC: 0.12 (IC025 - 0.62 - IC075 0.85)	ROR 0.59 (95% CI: 0.45-0.77)	IC: -0.18 (IC025 - 0.65 - IC075 0.29)	ROR 0.89 (95% CI: 0.68-1.17)	IC: -0.04 (IC025 - 0.51 - IC075 0.42)
Eructation	ROR 0.94 (95% CI: 0.65-1.35)	IC: -0.06 (IC025 - 1.00 - IC075 0.89)	ROR 0.88 (95% CI: 0.64-1.20)	IC: -0.15 (IC025 - 1.10 - IC075 0.79)	ROR 1.07 (95% CI: 0.74-1.54)	IC: 0.04 (IC025 - 0.70 - IC075 0.78)	ROR 0.94 (95% CI: 0.72-1.22)	IC: -0.07 (IC025 - 0.81 - IC075 0.67)	ROR 1.14 (95% CI: 0.83-1.57)	IC: 0.03 (IC025 - 0.40 - IC075 0.47)	ROR 1.07 (95% CI: 0.82-1.39)	IC: 0.02 (IC025 - 0.41 - IC075 0.46)
Flatulence	ROR 1.34 (95% CI: 0.82-2.19)	IC: 0.23 (IC025 - 0.94 - IC075 1.41)	ROR 1.18 (95% CI: 0.78-1.77)	IC: 0.18 (IC025 - 0.99 - IC075 1.36)	ROR 0.75 (95% CI: 0.46-1.22)	IC: -0.18 (IC025 - 1.29 - IC075 0.93)	ROR 0.88 (95% CI: 0.60-1.30)	IC: -0.13 (IC025 - 1.24 - IC075 0.97)	ROR 0.85 (95% CI: 0.57-1.27)	IC: -0.05 (IC025 - 0.67 - IC075 0.58)	ROR 1.14 (95% CI: 0.77-1.67)	IC: 0.05 (IC025 - 0.58 - IC075 0.67)
Gastritis	ROR 1.60 (95% CI: 0.84-3.06)	IC: 0.36 (IC025 - 1.11 - IC075 1.83)	ROR 0.80 (95% CI: 0.49-1.30)	IC: -0.26 (IC025 - 1.74 - IC075 1.21)	ROR 0.62 (95% CI: 0.33-1.19)	IC: -0.30 (IC025 - 1.81 - IC075 1.21)	ROR 0.50 (95% CI: 0.30-0.82)	IC: -0.78 (IC025 - 2.29 - IC075 0.73)	ROR 1.26 (95% CI: 0.77-2.05)	IC: 0.06 (IC025 - 0.59 - IC075 0.70)	ROR 2.01 (95% CI: 1.22-3.32)	IC: 0.20 (IC025 - 0.44 - IC075 0.85)

Gastrooesophageal reflux disease	ROR 0.65 (95% CI: 0.44-0.94)	IC: -0.40 (IC025 - 1.44 - IC075 0.63)	ROR 0.61 (95% CI: 0.43-0.85)	IC: -0.59 (IC025 - 1.63 - IC075 0.44)	ROR 1.55 (95% CI: 1.06-2.26)	IC: 0.22 (IC025 - 0.46 - IC075 0.89)	ROR 0.94 (95% CI: 0.74-1.19)	IC: -0.06 (IC025 - 0.74 - IC075 0.61)	ROR 1.65 (95% CI: 1.18-2.31)	IC: 0.11 (IC025 - 0.28 - IC075 0.51)	ROR 1.06 (95% CI: 0.84-1.35)	IC: 0.02 (IC025 - 0.37 - IC075 0.42)
Impaired gastric emptying	ROR 0.24 (95% CI: 0.13-0.44)	IC: -1.49 (IC025 - 3.33 - IC075 0.35)	ROR 0.27 (95% CI: 0.15-0.48)	IC: -1.63 (IC025 - 3.47 - IC075 0.21)	ROR 4.16 (95% CI: 2.26-7.67)	IC: 0.51 (IC025 - 0.23 - IC075 1.26)	ROR 1.12 (95% CI: 0.85-1.46)	IC: 0.11 (IC025 - 0.63 - IC075 0.86)	ROR 3.72 (95% CI: 2.07-6.68)	IC: 0.22 (IC025 - 0.26 - IC075 0.69)	ROR 0.89 (95% CI: 0.68-1.17)	IC: -0.04 (IC025 - 0.52 - IC075 0.43)
Intestinal obstruction	ROR 0.69 (95% CI: 0.37-1.27)	IC: -0.34 (IC025 - 1.99 - IC075 1.32)	ROR 0.41 (95% CI: 0.24-0.71)	IC: -1.07 (IC025 - 2.72 - IC075 0.59)	ROR 1.45 (95% CI: 0.79-2.68)	IC: 0.19 (IC025 - 0.94 - IC075 1.31)	ROR 0.60 (95% CI: 0.41-0.88)	IC: -0.56 (IC025 - 1.68 - IC075 0.57)	ROR 2.41 (95% CI: 1.42-4.10)	IC: 0.17 (IC025 - 0.36 - IC075 0.70)	ROR 1.66 (95% CI: 1.14-2.42)	IC: 0.16 (IC025 - 0.37 - IC075 0.69)
Nausea	ROR 0.97 (95% CI: 0.87-1.09)	IC: -0.02 (IC025 - 0.29 - IC075 0.25)	ROR 1.31 (95% CI: 1.18-1.45)	IC: 0.27 (IC025 0.00 - IC075 0.55)	ROR 1.03 (95% CI: 0.92-1.16)	IC: 0.01 (IC025 - 0.20 - IC075 0.23)	ROR 1.35 (95% CI: 1.24-1.47)	IC: 0.27 (IC025 0.05 - IC075 0.49)	ROR 0.76 (95% CI: 0.69-0.84)	IC: -0.07 (IC025 - 0.22 - IC075 0.07)	ROR 0.74 (95% CI: 0.68-0.81)	IC: -0.11 (IC025 - 0.26 - IC075 0.04)
Pancreatitis	ROR 2.03 (95% CI: 1.73-2.39)	IC: 0.50 (IC025 0.16 - IC075 0.83)	ROR 1.24 (95% CI: 1.10-1.40)	IC: 0.23 (IC025 - 0.11 - IC075 0.56)	ROR 0.49 (95% CI: 0.42-0.58)	IC: -0.46 (IC025 - 0.84 - IC075 - 0.08)	ROR 0.61 (95% CI: 0.54-0.70)	IC: -0.52 (IC025 - 0.90 - IC075 - 0.14)	ROR 0.80 (95% CI: 0.71-0.91)	IC: -0.06 (IC025 - 0.24 - IC075 0.12)	ROR 1.64 (95% CI: 1.43-1.87)	IC: 0.15 (IC025 - 0.03 - IC075 0.33)
Pancreatitis acute	ROR 1.27 (95% CI: 0.97-1.67)	IC: 0.20 (IC025 - 0.46 - IC075 0.85)	ROR 0.95 (95% CI: 0.76-1.19)	IC: -0.06 (IC025 - 0.71 - IC075 0.60)	ROR 0.78 (95% CI: 0.60-1.03)	IC: -0.15 (IC025 - 0.75 - IC075 0.45)	ROR 0.75 (95% CI: 0.61-0.92)	IC: -0.31 (IC025 - 0.91 - IC075 0.29)	ROR 1.05 (95% CI: 0.84-1.31)	IC: 0.01 (IC025 - 0.30 - IC075 0.32)	ROR 1.34 (95% CI: 1.09-1.65)	IC: 0.10 (IC025 - 0.21 - IC075 0.41)
Vomiting	ROR 0.62 (95% CI: 0.54-0.72)	IC: -0.40 (IC025 - 0.79 - IC075 - 0.02)	ROR 0.66 (95% CI: 0.57-0.75)	IC: -0.46 (IC025 - 0.85 - IC075 - 0.08)	ROR 1.60 (95% CI: 1.38-1.85)	IC: 0.22 (IC025 - 0.03 - IC075 0.46)	ROR 1.05 (95% CI: 0.95-1.16)	IC: 0.05 (IC025 - 0.20 - IC075 0.30)	ROR 1.53 (95% CI: 1.34-1.74)	IC: 0.09 (IC025 - 0.06 - IC075 0.24)	ROR 0.95 (95% CI: 0.87-1.05)	IC: -0.02 (IC025 - 0.17 - IC075 0.13)
Asthenia	ROR 0.84 (95% CI: 0.56-1.25)	IC: -0.16 (IC025 - 1.22 - IC075 0.90)	ROR 0.95 (95% CI: 0.67-1.36)	IC: -0.06 (IC025 - 1.12 - IC075 1.01)	ROR 1.20 (95% CI: 0.80-1.79)	IC: 0.10 (IC025 - 0.69 - IC075 0.89)	ROR 1.14 (95% CI: 0.86-1.52)	IC: 0.13 (IC025 - 0.66 - IC075 0.92)	ROR 1.05 (95% CI: 0.73-1.50)	IC: 0.01 (IC025 - 0.49 - IC075 0.52)	ROR 0.88 (95% CI: 0.66-1.17)	IC: -0.05 (IC025 - 0.56 - IC075 0.46)

Fatigue	ROR 0.58 (95% CI: 0.46-0.72)	IC: -0.51 (IC025 - 1.14 - IC075 0.12)	ROR 0.87 (95% CI: 0.70-1.07)	IC: -0.16 (IC025 - 0.79 - IC075 0.47)	ROR 1.74 (95% CI: 1.38-2.18)	IC: 0.26 (IC025 - 0.13 - IC075 0.65)	ROR 1.51 (95% CI: 1.30-1.75)	IC: 0.40 (IC025 0.01 - IC075 0.79)	ROR 1.15 (95% CI: 0.93-1.42)	IC: 0.04 (IC025 - 0.25 - IC075 0.32)	ROR 0.66 (95% CI: 0.57-0.77)	IC: -0.18 (IC025 - 0.46 - IC075 0.11)
Injection site erythema	ROR 19.24 (95% CI: 12.03-30.78)	IC: 1.21 (IC025 0.79 - IC075 1.64)	ROR 2.59 (95% CI: 2.19-3.06)	IC: 0.95 (IC025 0.52 - IC075 1.38)	ROR 0.05 (95% CI: 0.03-0.08)	IC: -2.94 (IC025 - 4.41 - IC075 - 1.46)	ROR 0.13 (95% CI: 0.08-0.21)	IC: -2.46 (IC025 - 3.93 - IC075 - 0.99)	ROR 0.39 (95% CI: 0.33-0.46)	IC: -0.37 (IC025 - 0.69 - IC075 - 0.04)	ROR 7.43 (95% CI: 4.68-11.79)	IC: 0.37 (IC025 0.05 - IC075 0.70)
Injection site pain	ROR 1.38 (95% CI: 0.89-2.13)	IC: 0.26 (IC025 - 0.78 - IC075 1.29)	ROR 1.75 (95% CI: 1.20-2.54)	IC: 0.60 (IC025 - 0.44 - IC075 1.63)	ROR 0.72 (95% CI: 0.47-1.12)	IC: -0.20 (IC025 - 1.18 - IC075 0.79)	ROR 1.27 (95% CI: 0.88-1.82)	IC: 0.24 (IC025 - 0.75 - IC075 1.22)	ROR 0.57 (95% CI: 0.39-0.83)	IC: -0.19 (IC025 - 0.86 - IC075 0.48)	ROR 0.79 (95% CI: 0.55-1.13)	IC: -0.10 (IC025 - 0.77 - IC075 0.57)
Injection site pruritus	ROR 34.92 (95% CI: 16.35-74.54)	IC: 1.26 (IC025 0.74 - IC075 1.78)	ROR 2.54 (95% CI: 2.08-3.11)	IC: 0.95 (IC025 0.43 - IC075 1.47)	ROR 0.03 (95% CI: 0.01-0.06)	IC: -3.71 (IC025 - 6.09 - IC075 - 1.33)	ROR 0.07 (95% CI: 0.03-0.15)	IC: -3.26 (IC025 - 5.64 - IC075 - 0.88)	ROR 0.39 (95% CI: 0.32-0.48)	IC: -0.36 (IC025 - 0.76 - IC075 0.04)	ROR 13.73 (95% CI: 6.48-29.10)	IC: 0.41 (IC025 0.01 - IC075 0.81)
Injection site reaction	ROR 15.32 (95% CI: 5.48-42.86)	IC: 1.17 (IC025 0.15 - IC075 2.19)	ROR 0.63 (95% CI: 0.45-0.88)	IC: -0.54 (IC025 - 1.56 - IC075 0.48)	ROR 0.07 (95% CI: 0.02-0.18)	IC: -2.59 (IC025 - 5.65 - IC075 0.48)	ROR 0.04 (95% CI: 0.02-0.11)	IC: -3.99 (IC025 - 7.06 - IC075 - 0.92)	ROR 1.58 (95% CI: 1.13-2.21)	IC: 0.10 (IC025 - 0.29 - IC075 0.50)	ROR 24.23 (95% CI: 9.02-65.06)	IC: 0.42 (IC025 0.02 - IC075 0.82)
Injection site urticaria	ROR 17.65 (95% CI: 6.35-49.07)	IC: 1.19 (IC025 0.24 - IC075 2.14)	ROR 3.54 (95% CI: 2.39-5.25)	IC: 1.22 (IC025 0.27 - IC075 2.18)	ROR 0.06 (95% CI: 0.02-0.16)	IC: -2.77 (IC025 - 5.84 - IC075 0.30)	ROR 0.20 (95% CI: 0.07-0.55)	IC: -1.85 (IC025 - 4.91 - IC075 1.22)	ROR 0.28 (95% CI: 0.19-0.42)	IC: -0.56 (IC025 - 1.44 - IC075 0.31)	ROR 4.99 (95% CI: 1.81-13.76)	IC: 0.34 (IC025 - 0.53 - IC075 1.21)
Malaise	ROR 0.87 (95% CI: 0.62-1.23)	IC: -0.12 (IC025 - 1.01 - IC075 0.77)	ROR 1.14 (95% CI: 0.84-1.55)	IC: 0.15 (IC025 - 0.74 - IC075 1.04)	ROR 1.15 (95% CI: 0.81-1.61)	IC: 0.07 (IC025 - 0.60 - IC075 0.75)	ROR 1.31 (95% CI: 1.02-1.68)	IC: 0.27 (IC025 - 0.41 - IC075 0.95)	ROR 0.87 (95% CI: 0.64-1.19)	IC: -0.04 (IC025 - 0.50 - IC075 0.42)	ROR 0.76 (95% CI: 0.60-0.98)	IC: -0.11 (IC025 - 0.58 - IC075 0.35)
Cholecystitis	ROR 1.27 (95% CI: 0.90-1.79)	IC: 0.20 (IC025 - 0.63 - IC075 1.02)	ROR 1.06 (95% CI: 0.80-1.41)	IC: 0.07 (IC025 - 0.76 - IC075 0.89)	ROR 0.79 (95% CI: 0.56-1.11)	IC: -0.15 (IC025 - 0.90 - IC075 0.61)	ROR 0.83 (95% CI: 0.64-1.08)	IC: -0.20 (IC025 - 0.95 - IC075 0.56)	ROR 0.94 (95% CI: 0.71-1.25)	IC: -0.02 (IC025 - 0.43 - IC075 0.40)	ROR 1.20 (95% CI: 0.92-1.56)	IC: 0.06 (IC025 - 0.35 - IC075 0.48)

Cholecystitis acute	ROR 1.07 (95% CI: 0.59-1.93)	IC: 0.06 (IC025 - 1.42 - IC075 1.53)	ROR 0.98 (95% CI: 0.60-1.62)	IC: -0.02 (IC025 - 1.49 - IC075 1.46)	ROR 0.94 (95% CI: 0.52-1.68)	IC: -0.04 (IC025 - 1.28 - IC075 1.20)	ROR 0.92 (95% CI: 0.60-1.42)	IC: -0.09 (IC025 - 1.33 - IC075 1.15)	ROR 1.02 (95% CI: 0.62-1.68)	IC: 0.00 (IC025 - 0.72 - IC075 0.72)	ROR 1.09 (95% CI: 0.70-1.68)	IC: 0.03 (IC025 - 0.69 - IC075 0.75)
Cholelithiasis	ROR 1.86 (95% CI: 1.50-2.29)	IC: 0.45 (IC025 - 0.01 - IC075 0.91)	ROR 1.14 (95% CI: 0.97-1.34)	IC: 0.14 (IC025 - 0.32 - IC075 0.60)	ROR 0.54 (95% CI: 0.44-0.67)	IC: -0.40 (IC025 - 0.91 - IC075 0.10)	ROR 0.61 (95% CI: 0.52-0.73)	IC: -0.53 (IC025 - 1.04 - IC075 - 0.02)	ROR 0.88 (95% CI: 0.75-1.03)	IC: -0.04 (IC025 - 0.27 - IC075 0.20)	ROR 1.63 (95% CI: 1.38-1.94)	IC: 0.15 (IC025 - 0.08 - IC075 0.39)
Gallbladder disorder	ROR 0.28 (95% CI: 0.15-0.54)	IC: -1.30 (IC025 - 3.22 - IC075 0.62)	ROR 0.82 (95% CI: 0.43-1.57)	IC: -0.22 (IC025 - 2.14 - IC075 1.70)	ROR 3.55 (95% CI: 1.87-6.77)	IC: 0.48 (IC025 - 0.36 - IC075 1.32)	ROR 2.93 (95% CI: 2.03-4.23)	IC: 0.94 (IC025 0.09 - IC075 1.78)	ROR 1.21 (95% CI: 0.64-2.32)	IC: 0.05 (IC025 - 0.82 - IC075 0.91)	ROR 0.34 (95% CI: 0.24-0.49)	IC: -0.59 (IC025 - 1.45 - IC075 0.28)
Anaphylactic reaction	ROR 1.28 (95% CI: 0.66-2.49)	IC: 0.20 (IC025 - 1.40 - IC075 1.80)	ROR 0.52 (95% CI: 0.31-0.87)	IC: -0.78 (IC025 - 2.39 - IC075 0.82)	ROR 0.78 (95% CI: 0.40-1.52)	IC: -0.15 (IC025 - 1.62 - IC075 1.32)	ROR 0.41 (95% CI: 0.25-0.66)	IC: -1.03 (IC025 - 2.50 - IC075 0.44)	ROR 1.93 (95% CI: 1.15-3.24)	IC: 0.14 (IC025 - 0.43 - IC075 0.71)	ROR 2.47 (95% CI: 1.52-4.00)	IC: 0.25 (IC025 - 0.32 - IC075 0.82)
Anaphylactic shock	ROR 1.09 (95% CI: 0.34-3.42)	IC: 0.06 (IC025 - 2.71 - IC075 2.84)	ROR 0.72 (95% CI: 0.28-1.87)	IC: -0.36 (IC025 - 3.13 - IC075 2.42)	ROR 0.92 (95% CI: 0.29-2.90)	IC: -0.05 (IC025 - 2.42 - IC075 2.33)	ROR 0.67 (95% CI: 0.29-1.52)	IC: -0.43 (IC025 - 2.80 - IC075 1.95)	ROR 1.38 (95% CI: 0.53-3.57)	IC: 0.08 (IC025 - 1.12 - IC075 1.28)	ROR 1.50 (95% CI: 0.66-3.43)	IC: 0.13 (IC025 - 1.07 - IC075 1.33)
Hypersensitivity	ROR 1.21 (95% CI: 0.75-1.94)	IC: 0.16 (IC025 - 1.00 - IC075 1.31)	ROR 0.95 (95% CI: 0.64-1.40)	IC: -0.06 (IC025 - 1.22 - IC075 1.10)	ROR 0.83 (95% CI: 0.51-1.33)	IC: -0.11 (IC025 - 1.15 - IC075 0.92)	ROR 0.78 (95% CI: 0.55-1.12)	IC: -0.26 (IC025 - 1.29 - IC075 0.78)	ROR 1.05 (95% CI: 0.71-1.56)	IC: 0.01 (IC025 - 0.54 - IC075 0.57)	ROR 1.27 (95% CI: 0.89-1.82)	IC: 0.08 (IC025 - 0.47 - IC075 0.64)
Amylase increased	ROR 1.13 (95% CI: 0.63-2.01)	IC: 0.10 (IC025 - 1.34 - IC075 1.54)	ROR 0.74 (95% CI: 0.46-1.18)	IC: -0.36 (IC025 - 1.80 - IC075 1.08)	ROR 0.89 (95% CI: 0.50-1.58)	IC: -0.07 (IC025 - 1.31 - IC075 1.17)	ROR 0.65 (95% CI: 0.43-0.99)	IC: -0.47 (IC025 - 1.71 - IC075 0.77)	ROR 1.36 (95% CI: 0.84-2.19)	IC: 0.07 (IC025 - 0.53 - IC075 0.68)	ROR 1.53 (95% CI: 1.01-2.33)	IC: 0.14 (IC025 - 0.47 - IC075 0.75)
Heart rate increased	ROR 1.84 (95% CI: 1.07-3.17)	IC: 0.45 (IC025 - 0.74 - IC075 1.65)	ROR 1.49 (95% CI: 0.97-2.28)	IC: 0.43 (IC025 - 0.77 - IC075 1.63)	ROR 0.54 (95% CI: 0.32-0.93)	IC: -0.41 (IC025 - 1.72 - IC075 0.91)	ROR 0.81 (95% CI: 0.51-1.27)	IC: -0.23 (IC025 - 1.54 - IC075 1.09)	ROR 0.67 (95% CI: 0.44-1.03)	IC: -0.13 (IC025 - 0.84 - IC075 0.59)	ROR 1.24 (95% CI: 0.79-1.95)	IC: 0.08 (IC025 - 0.64 - IC075 0.79)

Lipase increased	ROR 1.70 (95% CI: 1.13-2.55)	IC: 0.40 (IC025 - 0.51 - IC075 1.32)	ROR 0.90 (95% CI: 0.66-1.22)	IC: -0.12 (IC025 - 1.04 - IC075 0.79)	ROR 0.59 (95% CI: 0.39-0.88)	IC: -0.35 (IC025 - 1.31 - IC075 0.62)	ROR 0.53 (95% CI: 0.38-0.73)	IC: -0.71 (IC025 - 1.67 - IC075 0.25)	ROR 1.11 (95% CI: 0.82-1.51)	IC: 0.03 (IC025 - 0.40 - IC075 0.45)	ROR 1.89 (95% CI: 1.37-2.60)	IC: 0.19 (IC025 - 0.23 - IC075 0.61)
Dehydration	ROR 0.69 (95% CI: 0.49-0.96)	IC: -0.34 (IC025 - 1.24 - IC075 0.57)	ROR 0.45 (95% CI: 0.33-0.60)	IC: -0.97 (IC025 - 1.88 - IC075 - 0.06)	ROR 1.45 (95% CI: 1.04-2.03)	IC: 0.19 (IC025 - 0.42 - IC075 0.80)	ROR 0.65 (95% CI: 0.53-0.80)	IC: -0.47 (IC025 - 1.08 - IC075 0.14)	ROR 2.23 (95% CI: 1.67-2.99)	IC: 0.16 (IC025 - 0.14 - IC075 0.46)	ROR 1.54 (95% CI: 1.25-1.90)	IC: 0.14 (IC025 - 0.16 - IC075 0.44)
Dizziness	ROR 0.99 (95% CI: 0.76-1.28)	IC: -0.01 (IC025 - 0.66 - IC075 0.64)	ROR 1.10 (95% CI: 0.88-1.37)	IC: 0.10 (IC025 - 0.55 - IC075 0.76)	ROR 1.01 (95% CI: 0.78-1.31)	IC: 0.01 (IC025 - 0.52 - IC075 0.53)	ROR 1.11 (95% CI: 0.92-1.34)	IC: 0.11 (IC025 - 0.42 - IC075 0.63)	ROR 0.91 (95% CI: 0.73-1.14)	IC: -0.03 (IC025 - 0.36 - IC075 0.31)	ROR 0.90 (95% CI: 0.74-1.09)	IC: -0.04 (IC025 - 0.37 - IC075 0.29)
Dysaesthesia	NA	NA	NA	NA	NA	NA	ROR 10.47 (95% CI: 5.02-21.85)	IC: 1.53 (IC025 0.42 - IC075 2.64)	NA	NA	ROR 0.10 (95% CI: 0.05-0.20)	IC: -1.75 (IC025 - 3.87 - IC075 0.36)
Dysgeusia	ROR 1.05 (95% CI: 0.45-2.47)	IC: 0.04 (IC025 - 2.07 - IC075 2.15)	ROR 1.08 (95% CI: 0.52-2.25)	IC: 0.08 (IC025 - 2.03 - IC075 2.20)	ROR 0.95 (95% CI: 0.41-2.22)	IC: -0.03 (IC025 - 1.80 - IC075 1.74)	ROR 1.02 (95% CI: 0.54-1.94)	IC: 0.02 (IC025 - 1.75 - IC075 1.80)	ROR 0.93 (95% CI: 0.44-1.93)	IC: -0.02 (IC025 - 1.11 - IC075 1.07)	ROR 0.98 (95% CI: 0.52-1.85)	IC: -0.01 (IC025 - 1.10 - IC075 1.08)
Headache	ROR 1.00 (95% CI: 0.80-1.26)	IC: 0.00 (IC025 - 0.56 - IC075 0.57)	ROR 1.47 (95% CI: 1.20-1.80)	IC: 0.42 (IC025 - 0.15 - IC075 0.99)	ROR 1.00 (95% CI: 0.79-1.25)	IC: 0.00 (IC025 - 0.46 - IC075 0.46)	ROR 1.46 (95% CI: 1.23-1.74)	IC: 0.37 (IC025 - 0.09 - IC075 0.83)	ROR 0.68 (95% CI: 0.56-0.83)	IC: -0.12 (IC025 - 0.45 - IC075 0.21)	ROR 0.68 (95% CI: 0.57-0.81)	IC: -0.16 (IC025 - 0.50 - IC075 0.17)
Lethargy	ROR 2.29 (95% CI: 0.81-6.43)	IC: 0.56 (IC025 - 1.55 - IC075 2.67)	ROR 1.11 (95% CI: 0.53-2.32)	IC: 0.12 (IC025 - 2.00 - IC075 2.23)	ROR 0.44 (95% CI: 0.16-1.23)	IC: -0.56 (IC025 - 3.11 - IC075 2.00)	ROR 0.49 (95% CI: 0.20-1.16)	IC: -0.78 (IC025 - 3.33 - IC075 1.78)	ROR 0.90 (95% CI: 0.43-1.88)	IC: -0.03 (IC025 - 1.14 - IC075 1.08)	ROR 2.06 (95% CI: 0.86-4.90)	IC: 0.21 (IC025 - 0.90 - IC075 1.32)
Insomnia	ROR 1.07 (95% CI: 0.65-1.77)	IC: 0.06 (IC025 - 1.21 - IC075 1.32)	ROR 1.66 (95% CI: 1.05-2.61)	IC: 0.54 (IC025 - 0.72 - IC075 1.81)	ROR 0.93 (95% CI: 0.56-1.54)	IC: -0.04 (IC025 - 1.10 - IC075 1.02)	ROR 1.55 (95% CI: 1.03-2.32)	IC: 0.43 (IC025 - 0.64 - IC075 1.49)	ROR 0.60 (95% CI: 0.38-0.95)	IC: -0.17 (IC025 - 0.97 - IC075 0.63)	ROR 0.65 (95% CI: 0.43-0.97)	IC: -0.19 (IC025 - 0.99 - IC075 0.60)

Renal failure	ROR 1.27 (95% CI: 0.55-2.94)	IC: 0.19 (IC025 - 1.82 - IC075 2.20)	ROR 0.53 (95% CI: 0.27-1.02)	IC: -0.75 (IC025 - 2.76 - IC075 1.26)	ROR 0.79 (95% CI: 0.34-1.82)	IC: -0.14 (IC025 - 1.98 - IC075 1.70)	ROR 0.42 (95% CI: 0.23-0.77)	IC: -0.98 (IC025 - 2.83 - IC075 0.86)	ROR 1.89 (95% CI: 0.98-3.64)	IC: 0.14 (IC025 - 0.59 - IC075 0.86)	ROR 2.40 (95% CI: 1.30-4.40)	IC: 0.24 (IC025 - 0.49 - IC075 0.97)
Alopecia	ROR 0.28 (95% CI: 0.17-0.46)	IC: -1.33 (IC025 - 2.84 - IC075 0.18)	ROR 0.30 (95% CI: 0.19-0.48)	IC: -1.49 (IC025 - 3.01 - IC075 0.02)	ROR 3.62 (95% CI: 2.18-5.99)	IC: 0.48 (IC025 - 0.17 - IC075 1.13)	ROR 1.08 (95% CI: 0.85-1.37)	IC: 0.08 (IC025 - 0.57 - IC075 0.74)	ROR 3.34 (95% CI: 2.07-5.40)	IC: 0.21 (IC025 - 0.20 - IC075 0.62)	ROR 0.92 (95% CI: 0.73-1.17)	IC: -0.03 (IC025 - 0.44 - IC075 0.38)
Angioedema	ROR 0.52 (95% CI: 0.26-1.04)	IC: -0.61 (IC025 - 2.53 - IC075 1.31)	ROR 0.57 (95% CI: 0.30-1.07)	IC: -0.66 (IC025 - 2.58 - IC075 1.26)	ROR 1.91 (95% CI: 0.96-3.80)	IC: 0.30 (IC025 - 0.84 - IC075 1.44)	ROR 1.09 (95% CI: 0.72-1.64)	IC: 0.09 (IC025 - 1.05 - IC075 1.23)	ROR 1.76 (95% CI: 0.94-3.30)	IC: 0.12 (IC025 - 0.60 - IC075 0.84)	ROR 0.92 (95% CI: 0.61-1.38)	IC: -0.03 (IC025 - 0.75 - IC075 0.69)
Dermatitis allergic	ROR 2.86 (95% CI: 1.21-6.76)	IC: 0.69 (IC025 - 0.96 - IC075 2.34)	ROR 2.34 (95% CI: 1.24-4.40)	IC: 0.85 (IC025 - 0.80 - IC075 2.51)	ROR 0.35 (95% CI: 0.15-0.82)	IC: -0.76 (IC025 - 2.99 - IC075 1.47)	ROR 0.82 (95% CI: 0.37-1.80)	IC: -0.21 (IC025 - 2.44 - IC075 2.02)	ROR 0.43 (95% CI: 0.23-0.81)	IC: -0.32 (IC025 - 1.56 - IC075 0.92)	ROR 1.23 (95% CI: 0.56-2.70)	IC: 0.07 (IC025 - 1.17 - IC075 1.31)
Eczema	ROR 0.61 (95% CI: 0.12-3.14)	IC: -0.39 (IC025 - 4.51 - IC075 3.73)	ROR 0.44 (95% CI: 0.10-1.90)	IC: -0.86 (IC025 - 4.98 - IC075 3.26)	ROR 1.64 (95% CI: 0.32-8.46)	IC: 0.22 (IC025 - 2.56 - IC075 2.99)	ROR 0.72 (95% CI: 0.27-1.94)	IC: -0.33 (IC025 - 3.10 - IC075 2.45)	ROR 2.26 (95% CI: 0.53-9.73)	IC: 0.16 (IC025 - 1.31 - IC075 1.63)	ROR 1.38 (95% CI: 0.52-3.70)	IC: 0.11 (IC025 - 1.37 - IC075 1.58)
Erythema	ROR 1.99 (95% CI: 1.11-3.57)	IC: 0.50 (IC025 - 0.76 - IC075 1.76)	ROR 1.44 (95% CI: 0.92-2.25)	IC: 0.40 (IC025 - 0.87 - IC075 1.66)	ROR 0.50 (95% CI: 0.28-0.90)	IC: -0.46 (IC025 - 1.90 - IC075 0.97)	ROR 0.72 (95% CI: 0.44-1.19)	IC: -0.35 (IC025 - 1.78 - IC075 1.09)	ROR 0.69 (95% CI: 0.44-1.09)	IC: -0.12 (IC025 - 0.86 - IC075 0.63)	ROR 1.38 (95% CI: 0.84-2.26)	IC: 0.11 (IC025 - 0.63 - IC075 0.85)
Rash	ROR 1.67 (95% CI: 1.11-2.52)	IC: 0.39 (IC025 - 0.54 - IC075 1.33)	ROR 1.33 (95% CI: 0.96-1.84)	IC: 0.32 (IC025 - 0.62 - IC075 1.25)	ROR 0.60 (95% CI: 0.40-0.90)	IC: -0.33 (IC025 - 1.31 - IC075 0.64)	ROR 0.80 (95% CI: 0.57-1.11)	IC: -0.24 (IC025 - 1.22 - IC075 0.73)	ROR 0.75 (95% CI: 0.54-1.04)	IC: -0.09 (IC025 - 0.61 - IC075 0.44)	ROR 1.26 (95% CI: 0.90-1.76)	IC: 0.08 (IC025 - 0.45 - IC075 0.61)
Urticaria	ROR 1.97 (95% CI: 1.32-2.96)	IC: 0.50 (IC025 - 0.38 - IC075 1.38)	ROR 1.80 (95% CI: 1.30-2.47)	IC: 0.63 (IC025 - 0.26 - IC075 1.51)	ROR 0.51 (95% CI: 0.34-0.76)	IC: -0.46 (IC025 - 1.46 - IC075 0.54)	ROR 0.91 (95% CI: 0.64-1.29)	IC: -0.10 (IC025 - 1.10 - IC075 0.90)	ROR 0.56 (95% CI: 0.40-0.77)	IC: -0.20 (IC025 - 0.78 - IC075 0.37)	ROR 1.10 (95% CI: 0.77-1.56)	IC: 0.03 (IC025 - 0.54 - IC075 0.61)

Hypotension	ROR 0.83 (95% CI: 0.47-1.48)	IC: -0.16 (IC025 - 1.67 - IC075 1.35)	ROR 0.48 (95% CI: 0.30-0.79)	IC: -0.88 (IC025 - 2.39 - IC075 0.64)	ROR 1.20 (95% CI: 0.68-2.14)	IC: 0.10 (IC025 - 1.03 - IC075 1.22)	ROR 0.58 (95% CI: 0.40-0.85)	IC: -0.60 (IC025 - 1.73 - IC075 0.52)	ROR 2.07 (95% CI: 1.27-3.38)	IC: 0.15 (IC025 - 0.37 - IC075 0.67)	ROR 1.72 (95% CI: 1.18-2.51)	IC: 0.17 (IC025 - 0.35 - IC075 0.69)
Orthostatic hypotension	ROR 0.76 (95% CI: 0.23-2.53)	IC: -0.22 (IC025 - 3.29 - IC075 2.85)	ROR 0.84 (95% CI: 0.29-2.46)	IC: -0.19 (IC025 - 3.25 - IC075 2.88)	ROR 1.31 (95% CI: 0.39-4.36)	IC: 0.13 (IC025 - 2.10 - IC075 2.37)	ROR 1.10 (95% CI: 0.48-2.50)	IC: 0.09 (IC025 - 2.14 - IC075 2.33)	ROR 1.19 (95% CI: 0.41-3.49)	IC: 0.04 (IC025 - 1.39 - IC075 1.48)	ROR 0.91 (95% CI: 0.40-2.06)	IC: -0.04 (IC025 - 1.47 - IC075 1.40)

Supplementary Table S9. Disproportionality analysis of ADRs listed in the SmPCs of liraglutide, semaglutide, and tirzepatide and reported by both categories (HPs and non-HPs professionals). CI – confident interval, IC – information component, LIR – liraglutide, PT – preferred term, ROR – reporting odds ratio, SEM – semaglutide, TIR - tirzepatide

PT	LIR-SEM		LIR-TIR		SEM-LIR		SEM-TIR		TIR-LIR		TIR-SEM	
	ROR (95% CI: minim - maxim)	IC (IC025 - IC075)	ROR (95% CI: minim - maxim)	IC (IC025 - IC075)	ROR (95% CI: minim - maxim)	IC (IC025 - IC075)	ROR (95% CI: minim - maxim)	IC (IC025 - IC075)	ROR (95% CI: minim - maxim)	IC (IC025 - IC075)	ROR (95% CI: minim - maxim)	IC (IC025 - IC075)
Sinus tachycardia	ROR 1.89 (95% CI: 0.51-7.04)	IC: 0.43 (IC025 - 2.35 - IC075 3.21)	ROR 3.50 (95% CI: 1.07-11.48)	IC: 1.07 (IC025 - 1.70 - IC075 3.85)	ROR 0.53 (95% CI: 0.14-1.97)	IC: -0.39 (IC025 - 3.46 - IC075 2.67)	ROR 1.85 (95% CI: 0.52-6.57)	IC: 0.52 (IC025 - 2.55 - IC075 3.58)	ROR 0.29 (95% CI: 0.09-0.94)	IC: -0.53 (IC025 - 3.08 - IC075 2.02)	ROR 0.54 (95% CI: 0.15-1.91)	IC: -0.27 (IC025 - 2.83 - IC075 2.28)
Tachycardia	ROR 1.41 (95% CI: 0.96-2.06)	IC: 0.27 (IC025 - 0.62 - IC075 1.17)	ROR 1.20 (95% CI: 0.88-1.64)	IC: 0.21 (IC025 - 0.69 - IC075 1.10)	ROR 0.71 (95% CI: 0.49-1.04)	IC: -0.21 (IC025 - 1.08 - IC075 0.65)	ROR 0.85 (95% CI: 0.63-1.15)	IC: -0.17 (IC025 - 1.03 - IC075 0.70)	ROR 0.83 (95% CI: 0.61-1.14)	IC: -0.05 (IC025 - 0.53 - IC075 0.43)	ROR 1.17 (95% CI: 0.87-1.58)	IC: 0.06 (IC025 - 0.42 - IC075 0.54)
Optic ischemic neuropathy	ROR 0.24 (95% CI: 0.13-0.44)	IC: -1.49 (IC025 - 3.33 - IC075 0.35)	ROR 0.64 (95% CI: 0.35-1.19)	IC: -0.51 (IC025 - 2.35 - IC075 1.33)	ROR 4.17 (95% CI: 2.27-7.69)	IC: 0.51 (IC025 - 0.23 - IC075 1.26)	ROR 2.69 (95% CI: 1.96-3.70)	IC: 0.88 (IC025 0.13 - IC075 1.63)	ROR 1.55 (95% CI: 0.84-2.85)	IC: 0.10 (IC025 - 0.63 - IC075 0.83)	ROR 0.37 (95% CI: 0.27-0.51)	IC: -0.53 (IC025 - 1.26 - IC075 0.21)
Abdominal discomfort	ROR 0.95 (95% CI: 0.70-1.27)	IC: -0.05 (IC025 - 0.81 - IC075 0.71)	ROR 0.92 (95% CI: 0.71-1.19)	IC: -0.09 (IC025 - 0.86 - IC075 0.67)	ROR 1.06 (95% CI: 0.79-1.42)	IC: 0.03 (IC025 - 0.57 - IC075 0.63)	ROR 0.98 (95% CI: 0.79-1.21)	IC: -0.03 (IC025 - 0.63 - IC075 0.58)	ROR 1.08 (95% CI: 0.83-1.40)	IC: 0.02 (IC025 - 0.34 - IC075 0.38)	ROR 1.02 (95% CI: 0.83-1.27)	IC: 0.01 (IC025 - 0.35 - IC075 0.37)
Abdominal distension	ROR 1.18 (95% CI: 0.93-1.50)	IC: 0.14 (IC025 - 0.44 - IC075 0.71)	ROR 1.17 (95% CI: 0.95-1.42)	IC: 0.17 (IC025 - 0.41 - IC075 0.75)	ROR 0.85 (95% CI: 0.67-1.07)	IC: -0.10 (IC025 - 0.60 - IC075 0.41)	ROR 0.99 (95% CI: 0.82-1.18)	IC: -0.01 (IC025 - 0.52 - IC075 0.49)	ROR 0.86 (95% CI: 0.71-1.05)	IC: -0.04 (IC025 - 0.34 - IC075 0.26)	ROR 1.01 (95% CI: 0.85-1.22)	IC: 0.01 (IC025 - 0.30 - IC075 0.31)
Abdominal pain	ROR 0.72 (95% CI: 0.62-0.83)	IC: -0.28 (IC025 - 0.66 - IC075 0.10)	ROR 0.68 (95% CI: 0.60-0.77)	IC: -0.43 (IC025 - 0.81 - IC075 - 0.04)	ROR 1.39 (95% CI: 1.20-1.61)	IC: 0.16 (IC025 - 0.10 - IC075 0.42)	ROR 0.94 (95% CI: 0.85-1.04)	IC: -0.06 (IC025 - 0.32 - IC075 0.21)	ROR 1.43 (95% CI: 1.25-1.62)	IC: 0.08 (IC025 - 0.07 - IC075 0.23)	ROR 1.06 (95% CI: 0.96-1.17)	IC: 0.02 (IC025 - 0.13 - IC075 0.17)
Abdominal pain lower	ROR 0.86 (95% CI: 0.36-2.06)	IC: -0.12 (IC025 - 2.36 - IC075 2.11)	ROR 0.88 (95% CI: 0.41-1.90)	IC: -0.14 (IC025 - 2.37 - IC075 2.10)	ROR 1.16 (95% CI: 0.48-2.76)	IC: 0.08 (IC025 - 1.63 - IC075 1.79)	ROR 1.02 (95% CI: 0.55-1.89)	IC: 0.02 (IC025 - 1.69 - IC075 1.73)	ROR 1.13 (95% CI: 0.53-2.43)	IC: 0.03 (IC025 - 1.02 - IC075 1.08)	ROR 0.98 (95% CI: 0.53-1.81)	IC: -0.01 (IC025 - 1.06 - IC075 1.04)

Abdominal pain upper	ROR 0.82 (95% CI: 0.69-0.97)	IC: -0.17 (IC025 - 0.60 - IC075 0.26)	ROR 0.83 (95% CI: 0.72-0.97)	IC: -0.20 (IC025 - 0.64 - IC075 0.23)	ROR 1.23 (95% CI: 1.04-1.45)	IC: 0.10 (IC025 - 0.21 - IC075 0.42)	ROR 1.02 (95% CI: 0.91-1.15)	IC: 0.02 (IC025 - 0.30 - IC075 0.34)	ROR 1.18 (95% CI: 1.02-1.37)	IC: 0.04 (IC025 - 0.15 - IC075 0.23)	ROR 0.98 (95% CI: 0.87-1.10)	IC: -0.01 (IC025 - 0.20 - IC075 0.18)
Constipation	ROR 0.73 (95% CI: 0.63-0.84)	IC: -0.27 (IC025 - 0.64 - IC075 0.10)	ROR 0.86 (95% CI: 0.76-0.98)	IC: -0.16 (IC025 - 0.53 - IC075 0.21)	ROR 1.38 (95% CI: 1.19-1.59)	IC: 0.15 (IC025 - 0.10 - IC075 0.41)	ROR 1.18 (95% CI: 1.07-1.31)	IC: 0.16 (IC025 - 0.10 - IC075 0.42)	ROR 1.15 (95% CI: 1.01-1.31)	IC: 0.03 (IC025 - 0.13 - IC075 0.20)	ROR 0.84 (95% CI: 0.76-0.93)	IC: -0.06 (IC025 - 0.23 - IC075 0.10)
Diarrhoea	ROR 0.71 (95% CI: 0.63-0.80)	IC: -0.27 (IC025 - 0.55 - IC075 0.01)	ROR 0.61 (95% CI: 0.55-0.67)	IC: -0.50 (IC025 - 0.79 - IC075 - 0.22)	ROR 1.41 (95% CI: 1.26-1.58)	IC: 0.15 (IC025 - 0.04 - IC075 0.35)	ROR 0.86 (95% CI: 0.80-0.93)	IC: -0.13 (IC025 - 0.33 - IC075 0.07)	ROR 1.52 (95% CI: 1.38-1.68)	IC: 0.08 (IC025 - 0.02 - IC075 0.19)	ROR 1.16 (95% CI: 1.08-1.26)	IC: 0.05 (IC025 - 0.06 - IC075 0.15)
Dry mouth	ROR 1.60 (95% CI: 1.11-2.32)	IC: 0.36 (IC025 - 0.48 - IC075 1.21)	ROR 2.17 (95% CI: 1.58-2.98)	IC: 0.81 (IC025 - 0.03 - IC075 1.65)	ROR 0.62 (95% CI: 0.43-0.90)	IC: -0.30 (IC025 - 1.17 - IC075 0.56)	ROR 1.35 (95% CI: 0.98-1.87)	IC: 0.30 (IC025 - 0.56 - IC075 1.17)	ROR 0.46 (95% CI: 0.33-0.63)	IC: -0.29 (IC025 - 0.89 - IC075 0.31)	ROR 0.74 (95% CI: 0.54-1.02)	IC: -0.13 (IC025 - 0.73 - IC075 0.48)
Dyspepsia	ROR 1.06 (95% CI: 0.87-1.30)	IC: 0.05 (IC025 - 0.45 - IC075 0.54)	ROR 1.16 (95% CI: 0.97-1.37)	IC: 0.16 (IC025 - 0.34 - IC075 0.66)	ROR 0.94 (95% CI: 0.77-1.15)	IC: -0.03 (IC025 - 0.45 - IC075 0.38)	ROR 1.09 (95% CI: 0.94-1.27)	IC: 0.09 (IC025 - 0.33 - IC075 0.50)	ROR 0.87 (95% CI: 0.73-1.04)	IC: -0.04 (IC025 - 0.30 - IC075 0.22)	ROR 0.92 (95% CI: 0.79-1.07)	IC: -0.03 (IC025 - 0.29 - IC075 0.23)
Eructation	ROR 0.86 (95% CI: 0.69-1.08)	IC: -0.13 (IC025 - 0.69 - IC075 0.44)	ROR 0.94 (95% CI: 0.78-1.14)	IC: -0.07 (IC025 - 0.64 - IC075 0.50)	ROR 1.16 (95% CI: 0.93-1.44)	IC: 0.08 (IC025 - 0.35 - IC075 0.51)	ROR 1.09 (95% CI: 0.93-1.28)	IC: 0.09 (IC025 - 0.34 - IC075 0.52)	ROR 1.06 (95% CI: 0.88-1.29)	IC: 0.02 (IC025 - 0.25 - IC075 0.28)	ROR 0.92 (95% CI: 0.78-1.07)	IC: -0.03 (IC025 - 0.30 - IC075 0.24)
Flatulence	ROR 1.15 (95% CI: 0.86-1.53)	IC: 0.11 (IC025 - 0.60 - IC075 0.82)	ROR 1.26 (95% CI: 0.99-1.62)	IC: 0.26 (IC025 - 0.45 - IC075 0.97)	ROR 0.87 (95% CI: 0.65-1.16)	IC: -0.08 (IC025 - 0.70 - IC075 0.54)	ROR 1.10 (95% CI: 0.88-1.38)	IC: 0.10 (IC025 - 0.52 - IC075 0.72)	ROR 0.79 (95% CI: 0.62-1.01)	IC: -0.07 (IC025 - 0.46 - IC075 0.32)	ROR 0.91 (95% CI: 0.73-1.13)	IC: -0.04 (IC025 - 0.43 - IC075 0.35)
Gastritis	ROR 2.09 (95% CI: 1.37-3.18)	IC: 0.53 (IC025 - 0.36 - IC075 1.43)	ROR 1.38 (95% CI: 1.01-1.89)	IC: 0.35 (IC025 - 0.54 - IC075 1.25)	ROR 0.48 (95% CI: 0.31-0.73)	IC: -0.51 (IC025 - 1.55 - IC075 0.54)	ROR 0.66 (95% CI: 0.46-0.94)	IC: -0.45 (IC025 - 1.50 - IC075 0.59)	ROR 0.72 (95% CI: 0.53-0.99)	IC: -0.10 (IC025 - 0.61 - IC075 0.41)	ROR 1.51 (95% CI: 1.06-2.16)	IC: 0.13 (IC025 - 0.38 - IC075 0.65)

Gastrooesophageal reflux disease	ROR 0.58 (95% CI: 0.45-0.75)	IC: -0.50 (IC025 - 1.23 - IC075 0.22)	ROR 0.70 (95% CI: 0.55-0.89)	IC: -0.41 (IC025 - 1.14 - IC075 0.31)	ROR 1.72 (95% CI: 1.33-2.24)	IC: 0.26 (IC025 - 0.19 - IC075 0.71)	ROR 1.21 (95% CI: 1.02-1.43)	IC: 0.19 (IC025 - 0.26 - IC075 0.64)	ROR 1.41 (95% CI: 1.11-1.80)	IC: 0.08 (IC025 - 0.21 - IC075 0.38)	ROR 0.83 (95% CI: 0.70-0.98)	IC: -0.08 (IC025 - 0.37 - IC075 0.22)
Impaired gastric emptying	ROR 0.36 (95% CI: 0.28-0.46)	IC: -1.02 (IC025 - 1.75 - IC075 - 0.29)	ROR 0.28 (95% CI: 0.22-0.36)	IC: -1.54 (IC025 - 2.27 - IC075 - 0.80)	ROR 2.81 (95% CI: 2.19-3.61)	IC: 0.42 (IC025 0.06 - IC075 0.77)	ROR 0.80 (95% CI: 0.70-0.90)	IC: -0.23 (IC025 - 0.59 - IC075 0.13)	ROR 3.37 (95% CI: 2.68-4.26)	IC: 0.20 (IC025 0.01 - IC075 0.39)	ROR 1.26 (95% CI: 1.11-1.43)	IC: 0.08 (IC025 - 0.12 - IC075 0.27)
Intestinal obstruction	ROR 0.45 (95% CI: 0.31-0.67)	IC: -0.77 (IC025 - 1.89 - IC075 0.36)	ROR 0.32 (95% CI: 0.22-0.45)	IC: -1.42 (IC025 - 2.54 - IC075 - 0.29)	ROR 2.20 (95% CI: 1.49-3.26)	IC: 0.35 (IC025 - 0.27 - IC075 0.97)	ROR 0.70 (95% CI: 0.56-0.86)	IC: -0.39 (IC025 - 1.01 - IC075 0.23)	ROR 3.09 (95% CI: 2.16-4.40)	IC: 0.20 (IC025 - 0.12 - IC075 0.51)	ROR 1.43 (95% CI: 1.16-1.77)	IC: 0.12 (IC025 - 0.19 - IC075 0.43)
Nausea	ROR 0.82 (95% CI: 0.75-0.89)	IC: -0.13 (IC025 - 0.32 - IC075 0.06)	ROR 1.27 (95% CI: 1.17-1.38)	IC: 0.21 (IC025 0.01 - IC075 0.40)	ROR 1.22 (95% CI: 1.12-1.34)	IC: 0.08 (IC025 - 0.06 - IC075 0.22)	ROR 1.55 (95% CI: 1.45-1.66)	IC: 0.33 (IC025 0.19 - IC075 0.48)	ROR 0.84 (95% CI: 0.78-0.91)	IC: -0.04 (IC025 - 0.14 - IC075 0.06)	ROR 0.64 (95% CI: 0.60-0.69)	IC: -0.14 (IC025 - 0.24 - IC075 - 0.04)
Pancreatitis	ROR 2.00 (95% CI: 1.74-2.31)	IC: 0.48 (IC025 0.19 - IC075 0.77)	ROR 1.12 (95% CI: 1.01-1.25)	IC: 0.12 (IC025 - 0.18 - IC075 0.41)	ROR 0.50 (95% CI: 0.43-0.57)	IC: -0.43 (IC025 - 0.76 - IC075 - 0.11)	ROR 0.56 (95% CI: 0.50-0.63)	IC: -0.60 (IC025 - 0.93 - IC075 - 0.27)	ROR 0.91 (95% CI: 0.82-1.01)	IC: -0.03 (IC025 - 0.17 - IC075 0.12)	ROR 1.79 (95% CI: 1.59-2.00)	IC: 0.17 (IC025 0.02 - IC075 0.31)
Pancreatitis acute	ROR 1.33 (95% CI: 1.04-1.69)	IC: 0.22 (IC025 - 0.35 - IC075 0.80)	ROR 0.77 (95% CI: 0.63-0.94)	IC: -0.30 (IC025 - 0.88 - IC075 0.28)	ROR 0.75 (95% CI: 0.59-0.96)	IC: -0.17 (IC025 - 0.71 - IC075 0.37)	ROR 0.58 (95% CI: 0.48-0.70)	IC: -0.59 (IC025 - 1.13 - IC075 - 0.05)	ROR 1.28 (95% CI: 1.05-1.55)	IC: 0.06 (IC025 - 0.19 - IC075 0.31)	ROR 1.72 (95% CI: 1.43-2.07)	IC: 0.17 (IC025 - 0.08 - IC075 0.41)
Vomiting	ROR 0.65 (95% CI: 0.59-0.72)	IC: -0.32 (IC025 - 0.58 - IC075 - 0.07)	ROR 0.73 (95% CI: 0.67-0.81)	IC: -0.31 (IC025 - 0.56 - IC075 - 0.05)	ROR 1.53 (95% CI: 1.38-1.71)	IC: 0.18 (IC025 0.01 - IC075 0.35)	ROR 1.13 (95% CI: 1.05-1.21)	IC: 0.10 (IC025 - 0.08 - IC075 0.27)	ROR 1.28 (95% CI: 1.17-1.41)	IC: 0.05 (IC025 - 0.05 - IC075 0.16)	ROR 0.89 (95% CI: 0.83-0.96)	IC: -0.04 (IC025 - 0.14 - IC075 0.07)
Asthenia	ROR 0.96 (95% CI: 0.75-1.24)	IC: -0.03 (IC025 - 0.68 - IC075 0.61)	ROR 1.16 (95% CI: 0.93-1.45)	IC: 0.16 (IC025 - 0.48 - IC075 0.81)	ROR 1.04 (95% CI: 0.81-1.34)	IC: 0.02 (IC025 - 0.49 - IC075 0.53)	ROR 1.20 (95% CI: 1.00-1.45)	IC: 0.19 (IC025 - 0.33 - IC075 0.70)	ROR 0.86 (95% CI: 0.69-1.08)	IC: -0.04 (IC025 - 0.38 - IC075 0.29)	ROR 0.83 (95% CI: 0.69-1.00)	IC: -0.07 (IC025 - 0.41 - IC075 0.26)

Fatigue	ROR 0.68 (95% CI: 0.58-0.79)	IC: -0.33 (IC025 - 0.73 - IC075 0.06)	ROR 1.20 (95% CI: 1.04-1.38)	IC: 0.20 (IC025 - 0.20 - IC075 0.60)	ROR 1.48 (95% CI: 1.27-1.72)	IC: 0.18 (IC025 - 0.08 - IC075 0.45)	ROR 1.77 (95% CI: 1.59-1.98)	IC: 0.52 (IC025 0.25 - IC075 0.79)	ROR 0.84 (95% CI: 0.73-0.96)	IC: -0.05 (IC025 - 0.26 - IC075 0.16)	ROR 0.56 (95% CI: 0.51-0.63)	IC: -0.25 (IC025 - 0.46 - IC075 - 0.04)
Injection site erythema	ROR 16.84 (95% CI: 11.69-24.27)	IC: 1.19 (IC025 0.84 - IC075 1.55)	ROR 2.37 (95% CI: 2.06-2.72)	IC: 0.86 (IC025 0.50 - IC075 1.22)	ROR 0.06 (95% CI: 0.04-0.09)	IC: -2.74 (IC025 - 3.88 - IC075 - 1.60)	ROR 0.14 (95% CI: 0.10-0.20)	IC: -2.40 (IC025 - 3.54 - IC075 - 1.26)	ROR 0.44 (95% CI: 0.38-0.51)	IC: -0.30 (IC025 - 0.56 - IC075 - 0.04)	ROR 7.12 (95% CI: 4.99-10.17)	IC: 0.37 (IC025 0.11 - IC075 0.63)
Injection site pain	ROR 1.50 (95% CI: 1.12-2.00)	IC: 0.31 (IC025 - 0.36 - IC075 0.99)	ROR 1.55 (95% CI: 1.21-1.97)	IC: 0.47 (IC025 - 0.20 - IC075 1.15)	ROR 0.67 (95% CI: 0.50-0.89)	IC: -0.25 (IC025 - 0.92 - IC075 0.41)	ROR 1.03 (95% CI: 0.81-1.31)	IC: 0.03 (IC025 - 0.64 - IC075 0.70)	ROR 0.65 (95% CI: 0.51-0.83)	IC: -0.14 (IC025 - 0.55 - IC075 0.27)	ROR 0.97 (95% CI: 0.76-1.23)	IC: -0.01 (IC025 - 0.42 - IC075 0.40)
Injection site pruritus	ROR 33.08 (95% CI: 18.50-59.13)	IC: 1.26 (IC025 0.85 - IC075 1.67)	ROR 2.29 (95% CI: 1.95-2.68)	IC: 0.84 (IC025 0.43 - IC075 1.25)	ROR 0.03 (95% CI: 0.02-0.05)	IC: -3.64 (IC025 - 5.48 - IC075 - 1.80)	ROR 0.07 (95% CI: 0.04-0.12)	IC: -3.36 (IC025 - 5.20 - IC075 - 1.51)	ROR 0.46 (95% CI: 0.39-0.53)	IC: -0.29 (IC025 - 0.59 - IC075 0.01)	ROR 14.47 (95% CI: 8.15-25.69)	IC: 0.41 (IC025 0.11 - IC075 0.71)
Injection site reaction	ROR 15.05 (95% CI: 5.99-37.80)	IC: 1.17 (IC025 0.25 - IC075 2.10)	ROR 0.59 (95% CI: 0.44-0.80)	IC: -0.62 (IC025 - 1.54 - IC075 0.30)	ROR 0.07 (95% CI: 0.03-0.17)	IC: -2.59 (IC025 - 5.36 - IC075 0.19)	ROR 0.04 (95% CI: 0.02-0.10)	IC: -4.08 (IC025 - 6.86 - IC075 - 1.31)	ROR 1.66 (95% CI: 1.23-2.24)	IC: 0.11 (IC025 - 0.24 - IC075 0.46)	ROR 25.37 (95% CI: 10.49-61.38)	IC: 0.42 (IC025 0.08 - IC075 0.77)
Injection site urticaria	ROR 14.99 (95% CI: 6.88-32.66)	IC: 1.18 (IC025 0.39 - IC075 1.96)	ROR 3.48 (95% CI: 2.52-4.81)	IC: 1.21 (IC025 0.43 - IC075 2.00)	ROR 0.07 (95% CI: 0.03-0.15)	IC: -2.61 (IC025 - 4.98 - IC075 - 0.23)	ROR 0.23 (95% CI: 0.11-0.50)	IC: -1.70 (IC025 - 4.08 - IC075 0.67)	ROR 0.29 (95% CI: 0.21-0.40)	IC: -0.55 (IC025 - 1.26 - IC075 0.16)	ROR 4.30 (95% CI: 1.99-9.31)	IC: 0.33 (IC025 - 0.38 - IC075 1.04)
Malaise	ROR 0.97 (95% CI: 0.78-1.22)	IC: -0.02 (IC025 - 0.58 - IC075 0.54)	ROR 1.42 (95% CI: 1.16-1.73)	IC: 0.38 (IC025 - 0.18 - IC075 0.94)	ROR 1.03 (95% CI: 0.82-1.28)	IC: 0.01 (IC025 - 0.43 - IC075 0.46)	ROR 1.46 (95% CI: 1.23-1.73)	IC: 0.37 (IC025 - 0.08 - IC075 0.82)	ROR 0.71 (95% CI: 0.58-0.86)	IC: -0.11 (IC025 - 0.43 - IC075 0.22)	ROR 0.69 (95% CI: 0.58-0.81)	IC: -0.16 (IC025 - 0.48 - IC075 0.16)
Cholecystitis	ROR 1.25 (95% CI: 0.93-1.69)	IC: 0.18 (IC025 - 0.55 - IC075 0.91)	ROR 1.03 (95% CI: 0.80-1.32)	IC: 0.03 (IC025 - 0.70 - IC075 0.76)	ROR 0.80 (95% CI: 0.59-1.08)	IC: -0.13 (IC025 - 0.80 - IC075 0.53)	ROR 0.82 (95% CI: 0.65-1.03)	IC: -0.21 (IC025 - 0.88 - IC075 0.45)	ROR 0.97 (95% CI: 0.76-1.25)	IC: -0.01 (IC025 - 0.37 - IC075 0.35)	ROR 1.22 (95% CI: 0.97-1.54)	IC: 0.07 (IC025 - 0.29 - IC075 0.43)

Cholecystitis acute	ROR 0.92 (95% CI: 0.53-1.61)	IC: -0.07 (IC025 - 1.51 - IC075 1.37)	ROR 0.87 (95% CI: 0.54-1.42)	IC: -0.15 (IC025 - 1.59 - IC075 1.28)	ROR 1.09 (95% CI: 0.62-1.90)	IC: 0.05 (IC025 - 1.08 - IC075 1.17)	ROR 0.95 (95% CI: 0.64-1.41)	IC: -0.05 (IC025 - 1.18 - IC075 1.07)	ROR 1.15 (95% CI: 0.71-1.86)	IC: 0.04 (IC025 - 0.63 - IC075 0.70)	ROR 1.05 (95% CI: 0.71-1.57)	IC: 0.02 (IC025 - 0.64 - IC075 0.68)
Cholelithiasis	ROR 2.17 (95% CI: 1.81-2.60)	IC: 0.54 (IC025 0.17 - IC075 0.92)	ROR 1.17 (95% CI: 1.02-1.34)	IC: 0.17 (IC025 - 0.21 - IC075 0.54)	ROR 0.46 (95% CI: 0.38-0.55)	IC: -0.52 (IC025 - 0.96 - IC075 - 0.08)	ROR 0.54 (95% CI: 0.46-0.62)	IC: -0.67 (IC025 - 1.11 - IC075 - 0.23)	ROR 0.87 (95% CI: 0.76-1.00)	IC: -0.04 (IC025 - 0.23 - IC075 0.16)	ROR 1.86 (95% CI: 1.60-2.16)	IC: 0.18 (IC025 - 0.01 - IC075 0.38)
Gallbladder disorder	ROR 0.28 (95% CI: 0.18-0.46)	IC: -1.30 (IC025 - 2.74 - IC075 0.14)	ROR 0.63 (95% CI: 0.40-1.02)	IC: -0.54 (IC025 - 1.97 - IC075 0.90)	ROR 3.53 (95% CI: 2.19-5.70)	IC: 0.48 (IC025 - 0.15 - IC075 1.11)	ROR 2.24 (95% CI: 1.73-2.90)	IC: 0.74 (IC025 0.11 - IC075 1.37)	ROR 1.57 (95% CI: 0.98-2.51)	IC: 0.10 (IC025 - 0.46 - IC075 0.67)	ROR 0.45 (95% CI: 0.35-0.58)	IC: -0.40 (IC025 - 0.97 - IC075 0.16)
Anaphylactic reaction	ROR 1.45 (95% CI: 0.80-2.65)	IC: 0.29 (IC025 - 1.11 - IC075 1.69)	ROR 0.50 (95% CI: 0.31-0.78)	IC: -0.84 (IC025 - 2.25 - IC075 0.56)	ROR 0.69 (95% CI: 0.38-1.25)	IC: -0.23 (IC025 - 1.60 - IC075 1.14)	ROR 0.34 (95% CI: 0.22-0.53)	IC: -1.25 (IC025 - 2.62 - IC075 0.13)	ROR 1.99 (95% CI: 1.26-3.13)	IC: 0.14 (IC025 - 0.34 - IC075 0.63)	ROR 2.93 (95% CI: 1.88-4.57)	IC: 0.28 (IC025 - 0.21 - IC075 0.76)
Anaphylactic shock	ROR 1.35 (95% CI: 0.52-3.51)	IC: 0.23 (IC025 - 2.00 - IC075 2.46)	ROR 0.52 (95% CI: 0.25-1.09)	IC: -0.76 (IC025 - 2.99 - IC075 1.48)	ROR 0.74 (95% CI: 0.29-1.92)	IC: -0.18 (IC025 - 2.29 - IC075 1.93)	ROR 0.39 (95% CI: 0.19-0.78)	IC: -1.07 (IC025 - 3.18 - IC075 1.04)	ROR 1.94 (95% CI: 0.93-4.05)	IC: 0.14 (IC025 - 0.67 - IC075 0.95)	ROR 2.58 (95% CI: 1.28-5.19)	IC: 0.25 (IC025 - 0.56 - IC075 1.06)
Hypersensitivity	ROR 1.21 (95% CI: 0.84-1.76)	IC: 0.16 (IC025 - 0.75 - IC075 1.06)	ROR 0.91 (95% CI: 0.67-1.24)	IC: -0.10 (IC025 - 1.01 - IC075 0.80)	ROR 0.82 (95% CI: 0.57-1.19)	IC: -0.12 (IC025 - 0.92 - IC075 0.69)	ROR 0.75 (95% CI: 0.57-0.99)	IC: -0.31 (IC025 - 1.12 - IC075 0.50)	ROR 1.09 (95% CI: 0.80-1.48)	IC: 0.02 (IC025 - 0.40 - IC075 0.44)	ROR 1.33 (95% CI: 1.01-1.75)	IC: 0.10 (IC025 - 0.33 - IC075 0.52)
Amylase increased	ROR 1.34 (95% CI: 0.81-2.21)	IC: 0.23 (IC025 - 0.96 - IC075 1.43)	ROR 0.94 (95% CI: 0.63-1.41)	IC: -0.07 (IC025 - 1.26 - IC075 1.13)	ROR 0.75 (95% CI: 0.45-1.23)	IC: -0.18 (IC025 - 1.30 - IC075 0.94)	ROR 0.70 (95% CI: 0.48-1.03)	IC: -0.38 (IC025 - 1.50 - IC075 0.74)	ROR 1.02 (95% CI: 0.68-1.53)	IC: 0.01 (IC025 - 0.56 - IC075 0.58)	ROR 1.42 (95% CI: 0.97-2.08)	IC: 0.12 (IC025 - 0.45 - IC075 0.69)
Heart rate increased	ROR 1.79 (95% CI: 1.28-2.50)	IC: 0.44 (IC025 - 0.30 - IC075 1.18)	ROR 1.62 (95% CI: 1.24-2.12)	IC: 0.52 (IC025 - 0.22 - IC075 1.27)	ROR 0.56 (95% CI: 0.40-0.78)	IC: -0.39 (IC025 - 1.19 - IC075 0.42)	ROR 0.91 (95% CI: 0.68-1.20)	IC: -0.10 (IC025 - 0.91 - IC075 0.70)	ROR 0.63 (95% CI: 0.48-0.82)	IC: -0.15 (IC025 - 0.61 - IC075 0.31)	ROR 1.10 (95% CI: 0.83-1.46)	IC: 0.04 (IC025 - 0.42 - IC075 0.50)

Lipase increased	ROR 1.56 (95% CI: 1.08-2.24)	IC: 0.34 (IC025 - 0.50 - IC075 1.18)	ROR 0.91 (95% CI: 0.68-1.21)	IC: -0.11 (IC025 - 0.95 - IC075 0.73)	ROR 0.64 (95% CI: 0.45-0.93)	IC: -0.28 (IC025 - 1.13 - IC075 0.57)	ROR 0.58 (95% CI: 0.44-0.78)	IC: -0.59 (IC025 - 1.44 - IC075 0.26)	ROR 1.11 (95% CI: 0.83-1.47)	IC: 0.03 (IC025 - 0.36 - IC075 0.42)	ROR 1.71 (95% CI: 1.29-2.27)	IC: 0.17 (IC025 - 0.23 - IC075 0.56)
Dehydration	ROR 0.56 (95% CI: 0.44-0.71)	IC: -0.54 (IC025 - 1.20 - IC075 0.12)	ROR 0.36 (95% CI: 0.29-0.45)	IC: -1.22 (IC025 - 1.88 - IC075 - 0.56)	ROR 1.79 (95% CI: 1.41-2.27)	IC: 0.27 (IC025 - 0.13 - IC075 0.67)	ROR 0.65 (95% CI: 0.56-0.74)	IC: -0.46 (IC025 - 0.86 - IC075 - 0.05)	ROR 2.53 (95% CI: 2.05-3.13)	IC: 0.16 (IC025 - 0.03 - IC075 0.35)	ROR 1.54 (95% CI: 1.34-1.77)	IC: 0.14 (IC025 - 0.06 - IC075 0.33)
Dizziness	ROR 0.92 (95% CI: 0.77-1.08)	IC: -0.07 (IC025 - 0.49 - IC075 0.35)	ROR 1.23 (95% CI: 1.06-1.43)	IC: 0.23 (IC025 - 0.20 - IC075 0.65)	ROR 1.09 (95% CI: 0.92-1.29)	IC: 0.05 (IC025 - 0.28 - IC075 0.37)	ROR 1.35 (95% CI: 1.19-1.53)	IC: 0.29 (IC025 - 0.04 - IC075 0.61)	ROR 0.81 (95% CI: 0.70-0.95)	IC: -0.06 (IC025 - 0.28 - IC075 0.17)	ROR 0.74 (95% CI: 0.65-0.84)	IC: -0.12 (IC025 - 0.35 - IC075 0.11)
Dysaesthesia	NA	NA	NA	NA	NA	NA	ROR 8.32 (95% CI: 4.44-15.60)	IC: 1.46 (IC025 0.43 - IC075 2.50)	NA	NA	ROR 0.12 (95% CI: 0.06-0.23)	IC: -1.52 (IC025 - 3.29 - IC075 0.25)
Dysgeusia	ROR 0.84 (95% CI: 0.52-1.36)	IC: -0.15 (IC025 - 1.42 - IC075 1.11)	ROR 1.66 (95% CI: 1.05-2.61)	IC: 0.54 (IC025 - 0.72 - IC075 1.81)	ROR 1.19 (95% CI: 0.74-1.92)	IC: 0.09 (IC025 - 0.85 - IC075 1.03)	ROR 1.97 (95% CI: 1.35-2.87)	IC: 0.64 (IC025 - 0.31 - IC075 1.58)	ROR 0.58 (95% CI: 0.37-0.92)	IC: -0.18 (IC025 - 0.98 - IC075 0.62)	ROR 0.51 (95% CI: 0.35-0.74)	IC: -0.33 (IC025 - 1.12 - IC075 0.47)
Headache	ROR 1.08 (95% CI: 0.92-1.25)	IC: 0.06 (IC025 - 0.31 - IC075 0.42)	ROR 1.91 (95% CI: 1.67-2.20)	IC: 0.66 (IC025 0.30 - IC075 1.03)	ROR 0.93 (95% CI: 0.80-1.08)	IC: -0.04 (IC025 - 0.35 - IC075 0.27)	ROR 1.78 (95% CI: 1.57-2.01)	IC: 0.53 (IC025 0.22 - IC075 0.84)	ROR 0.54 (95% CI: 0.47-0.62)	IC: -0.21 (IC025 - 0.45 - IC075 0.03)	ROR 0.56 (95% CI: 0.50-0.64)	IC: -0.25 (IC025 - 0.50 - IC075 - 0.01)
Lethargy	ROR 1.74 (95% CI: 0.85-3.58)	IC: 0.41 (IC025 - 1.19 - IC075 2.01)	ROR 0.91 (95% CI: 0.53-1.56)	IC: -0.11 (IC025 - 1.71 - IC075 1.49)	ROR 0.57 (95% CI: 0.28-1.18)	IC: -0.36 (IC025 - 2.07 - IC075 1.35)	ROR 0.52 (95% CI: 0.29-0.92)	IC: -0.72 (IC025 - 2.43 - IC075 0.99)	ROR 1.12 (95% CI: 0.65-1.93)	IC: 0.03 (IC025 - 0.72 - IC075 0.78)	ROR 1.92 (95% CI: 1.08-3.40)	IC: 0.20 (IC025 - 0.56 - IC075 0.95)
Insomnia	ROR 1.03 (95% CI: 0.74-1.43)	IC: 0.03 (IC025 - 0.80 - IC075 0.85)	ROR 1.45 (95% CI: 1.08-1.95)	IC: 0.41 (IC025 - 0.42 - IC075 1.24)	ROR 0.97 (95% CI: 0.70-1.34)	IC: -0.02 (IC025 - 0.70 - IC075 0.66)	ROR 1.41 (95% CI: 1.09-1.82)	IC: 0.34 (IC025 - 0.34 - IC075 1.02)	ROR 0.68 (95% CI: 0.51-0.92)	IC: -0.12 (IC025 - 0.61 - IC075 0.37)	ROR 0.71 (95% CI: 0.55-0.92)	IC: -0.15 (IC025 - 0.63 - IC075 0.34)

Renal failure	ROR 1.27 (95% CI: 0.64-2.52)	IC: 0.19 (IC025 - 1.46 - IC075 1.84)	ROR 0.37 (95% CI: 0.22-0.63)	IC: -1.21 (IC025 - 2.86 - IC075 0.44)	ROR 0.79 (95% CI: 0.40-1.56)	IC: -0.14 (IC025 - 1.65 - IC075 1.37)	ROR 0.29 (95% CI: 0.18-0.47)	IC: -1.44 (IC025 - 2.96 - IC075 0.07)	ROR 2.66 (95% CI: 1.56-4.51)	IC: 0.18 (IC025 - 0.32 - IC075 0.68)	ROR 3.43 (95% CI: 2.11-5.58)	IC: 0.30 (IC025 - 0.20 - IC075 0.80)
Alopecia	ROR 0.31 (95% CI: 0.23-0.42)	IC: -1.18 (IC025 - 2.04 - IC075 - 0.31)	ROR 0.49 (95% CI: 0.37-0.65)	IC: -0.85 (IC025 - 1.72 - IC075 0.01)	ROR 3.20 (95% CI: 2.39-4.28)	IC: 0.45 (IC025 0.06 - IC075 0.84)	ROR 1.58 (95% CI: 1.35-1.84)	IC: 0.43 (IC025 0.04 - IC075 0.83)	ROR 2.09 (95% CI: 1.58-2.76)	IC: 0.15 (IC025 - 0.14 - IC075 0.45)	ROR 0.63 (95% CI: 0.54-0.74)	IC: -0.20 (IC025 - 0.49 - IC075 0.10)
Angioedema	ROR 0.48 (95% CI: 0.26-0.90)	IC: -0.70 (IC025 - 2.47 - IC075 1.07)	ROR 0.52 (95% CI: 0.29-0.92)	IC: -0.78 (IC025 - 2.55 - IC075 0.99)	ROR 2.08 (95% CI: 1.11-3.88)	IC: 0.33 (IC025 - 0.68 - IC075 1.33)	ROR 1.08 (95% CI: 0.75-1.55)	IC: 0.08 (IC025 - 0.93 - IC075 1.09)	ROR 1.95 (95% CI: 1.09-3.46)	IC: 0.14 (IC025 - 0.49 - IC075 0.77)	ROR 0.93 (95% CI: 0.65-1.33)	IC: -0.03 (IC025 - 0.66 - IC075 0.60)
Dermatitis allergic	ROR 1.88 (95% CI: 0.90-3.91)	IC: 0.46 (IC025 - 1.14 - IC075 2.06)	ROR 2.17 (95% CI: 1.19-3.97)	IC: 0.79 (IC025 - 0.81 - IC075 2.39)	ROR 0.53 (95% CI: 0.26-1.11)	IC: -0.42 (IC025 - 2.19 - IC075 1.36)	ROR 1.16 (95% CI: 0.60-2.21)	IC: 0.14 (IC025 - 1.63 - IC075 1.92)	ROR 0.48 (95% CI: 0.26-0.89)	IC: -0.27 (IC025 - 1.43 - IC075 0.89)	ROR 0.87 (95% CI: 0.45-1.65)	IC: -0.06 (IC025 - 1.22 - IC075 1.10)
Eczema	ROR 0.69 (95% CI: 0.24-1.99)	IC: -0.31 (IC025 - 3.09 - IC075 2.46)	ROR 0.70 (95% CI: 0.27-1.80)	IC: -0.40 (IC025 - 3.17 - IC075 2.38)	ROR 1.44 (95% CI: 0.50-4.16)	IC: 0.18 (IC025 - 1.74 - IC075 2.10)	ROR 1.01 (95% CI: 0.51-2.02)	IC: 0.01 (IC025 - 1.91 - IC075 1.93)	ROR 1.45 (95% CI: 0.56-3.73)	IC: 0.09 (IC025 - 1.09 - IC075 1.27)	ROR 0.99 (95% CI: 0.50-1.98)	IC: 0.00 (IC025 - 1.18 - IC075 1.17)
Erythema	ROR 2.22 (95% CI: 1.38-3.57)	IC: 0.57 (IC025 - 0.43 - IC075 1.57)	ROR 1.65 (95% CI: 1.16-2.37)	IC: 0.54 (IC025 - 0.45 - IC075 1.54)	ROR 0.45 (95% CI: 0.28-0.72)	IC: -0.56 (IC025 - 1.75 - IC075 0.64)	ROR 0.75 (95% CI: 0.49-1.12)	IC: -0.32 (IC025 - 1.51 - IC075 0.88)	ROR 0.63 (95% CI: 0.44-0.90)	IC: -0.16 (IC025 - 0.78 - IC075 0.47)	ROR 1.34 (95% CI: 0.89-2.02)	IC: 0.10 (IC025 - 0.53 - IC075 0.73)
Rash	ROR 1.64 (95% CI: 1.19-2.26)	IC: 0.38 (IC025 - 0.35 - IC075 1.11)	ROR 1.45 (95% CI: 1.12-1.88)	IC: 0.41 (IC025 - 0.32 - IC075 1.14)	ROR 0.61 (95% CI: 0.44-0.84)	IC: -0.32 (IC025 - 1.07 - IC075 0.43)	ROR 0.88 (95% CI: 0.68-1.15)	IC: -0.13 (IC025 - 0.88 - IC075 0.62)	ROR 0.68 (95% CI: 0.53-0.89)	IC: -0.12 (IC025 - 0.55 - IC075 0.31)	ROR 1.13 (95% CI: 0.87-1.47)	IC: 0.04 (IC025 - 0.38 - IC075 0.47)
Urticaria	ROR 1.88 (95% CI: 1.33-2.65)	IC: 0.47 (IC025 - 0.29 - IC075 1.23)	ROR 1.71 (95% CI: 1.30-2.25)	IC: 0.58 (IC025 - 0.19 - IC075 1.34)	ROR 0.53 (95% CI: 0.38-0.75)	IC: -0.42 (IC025 - 1.26 - IC075 0.42)	ROR 0.91 (95% CI: 0.68-1.23)	IC: -0.10 (IC025 - 0.94 - IC075 0.75)	ROR 0.64 (95% CI: 0.48-0.84)	IC: -0.16 (IC025 - 0.64 - IC075 0.33)	ROR 1.10 (95% CI: 0.82-1.47)	IC: 0.03 (IC025 - 0.45 - IC075 0.52)

Hypotension	ROR 1.01 (95% CI: 0.66-1.53)	IC: 0.01 (IC025 - 1.05 - IC075 1.07)	ROR 0.62 (95% CI: 0.44-0.88)	IC: -0.57 (IC025 - 1.63 - IC075 0.50)	ROR 0.99 (95% CI: 0.65-1.51)	IC: 0.00 (IC025 - 0.87 - IC075 0.86)	ROR 0.62 (95% CI: 0.46-0.82)	IC: -0.53 (IC025 - 1.40 - IC075 0.33)	ROR 1.69 (95% CI: 1.20-2.39)	IC: 0.12 (IC025 - 0.29 - IC075 0.53)	ROR 1.62 (95% CI: 1.21-2.17)	IC: 0.15 (IC025 - 0.26 - IC075 0.56)
Orthostatic hypotension	ROR 0.51 (95% CI: 0.16-1.58)	IC: -0.60 (IC025 - 3.67 - IC075 2.47)	ROR 0.51 (95% CI: 0.18-1.44)	IC: -0.76 (IC025 - 3.83 - IC075 2.31)	ROR 1.97 (95% CI: 0.63-6.11)	IC: 0.30 (IC025 - 1.54 - IC075 2.14)	ROR 1.00 (95% CI: 0.52-1.94)	IC: 0.00 (IC025 - 1.84 - IC075 1.84)	ROR 1.86 (95% CI: 0.66-5.24)	IC: 0.13 (IC025 - 1.00 - IC075 1.25)	ROR 1.00 (95% CI: 0.52-1.94)	IC: 0.00 (IC025 - 1.12 - IC075 1.12)
